# Signal Quality Evaluation of Emerging EEG Devices

**DOI:** 10.3389/fphys.2018.00098

**Published:** 2018-02-14

**Authors:** Thea Radüntz

**Affiliations:** Mental Health and Cognitive Capacity, Work and Health, Federal Institute for Occupational Safety and Health, Berlin, Germany

**Keywords:** signal quality, electroencephalogram (EEG), mobile EEG, dry electrodes, wearables

## Abstract

Electroencephalogram (EEG) registration as a direct measure of brain activity has unique potentials. It is one of the most reliable and predicative indicators when studying human cognition, evaluating a subject's health condition, or monitoring their mental state. Unfortunately, standard signal acquisition procedures limit the usability of EEG devices and narrow their application outside the lab. Emerging sensor technology allows gel-free EEG registration and wireless signal transmission. Thus, it enables quick and easy application of EEG devices by users themselves. Although a main requirement for the interpretation of an EEG is good signal quality, there is a lack of research on this topic in relation to new devices. In our work, we compared the signal quality of six very different EEG devices. On six consecutive days, 24 subjects wore each device for 60 min and completed tasks and games on the computer. The registered signals were evaluated in the time and frequency domains. In the time domain, we examined the percentage of artifact-contaminated EEG segments and the signal-to-noise ratios. In the frequency domain, we focused on the band power variation in relation to task demands. The results indicated that the signal quality of a mobile, gel-based EEG system could not be surpassed by that of a gel-free system. However, some of the mobile dry-electrode devices offered signals that were almost comparable and were very promising. This study provided a differentiated view of the signal quality of emerging mobile and gel-free EEG recording technology and allowed an assessment of the functionality of the new devices. Hence, it provided a crucial prerequisite for their general application, while simultaneously supporting their further development.

## 1. Introduction

Electroencephalogram (EEG) registration as a direct measurement of brain activity has unique potentials. The fact that all physical and mental processes are controlled by our brain suggests that such information is also reflected in the registered signal. Hence, an EEG is one of the most reliable and predicative indicators when studying human cognition, evaluating a subject's health condition, or monitoring their mental state.

A main requirement for the interpretation of the registered brain activity is good signal quality. A common way to achieve this is the registration of the EEG in a shielded lab and preparation of the subject's skin before the electrodes are placed to reduce the impedance. Unfortunately, these standard procedures limit the usability of an EEG device and narrow its application outside the lab. An additional challenge when it comes to real-life applications involves the wired connections from the electrode cap to an amplifier and computer. These severely restrict a subject's mobility and decrease user acceptance of the measuring technique.

Over the last few years, research engineers and EEG system manufacturers have been working on overcoming these issues and allowing easy and reliable EEG registration outside the lab. By means of wireless signal transmission, they have developed mobile devices that allow subjects to move more freely. Furthermore, emerging sensor technology allows gel-free EEG registration and enables quick and easy application of EEG devices by the users themselves. However, the signal quality of these new devices remains unclear.

There have only been a few articles dealing with this issue. Among these, there were studies that focused primarily on the evaluation of mobile vs. non-mobile devices, which neglected the emerging dry-electrode systems (Forney et al., 2013; Ries et al., 2014). Other investigations concentrated only on a single dry-electrode device and considered its general performance (Callan et al., 2015; Rogers et al., 2016). Finally, there were studies on one dry-electrode and one gel-based device (Zander et al., 2011; Johnstone et al., 2012; Duvinage et al., 2013). However, the majority of the articles described self-developed dry sensors and compared their signal quality to that of a traditional gel-based system (Sullivan et al., 2007; Nikulin et al., 2010; Grozea et al., 2011; Saab et al., 2011; Debener et al., 2012; Guger et al., 2012). An interesting study that examined more than two devices included a wireless gel-based device, wireless saline-based device, wired dry-electrode device, and wired gel-electrode device (Grummett et al., 2015). To the best of our knowledge, no signal comparison studies of several wireless dry-electrode systems are available.

In our work, we compared the signal quality of various mobile and gel-free EEG devices. Hence, our study offers a differentiated look at nascent EEG recording technology and enables functionality assessments of the new devices. The obtained results build a crucial prerequisite for the general application of the emerging devices outside the lab and simultaneously support their further development.

## 2. Materials and experiments

### 2.1. EEG systems

**Figure 1:**
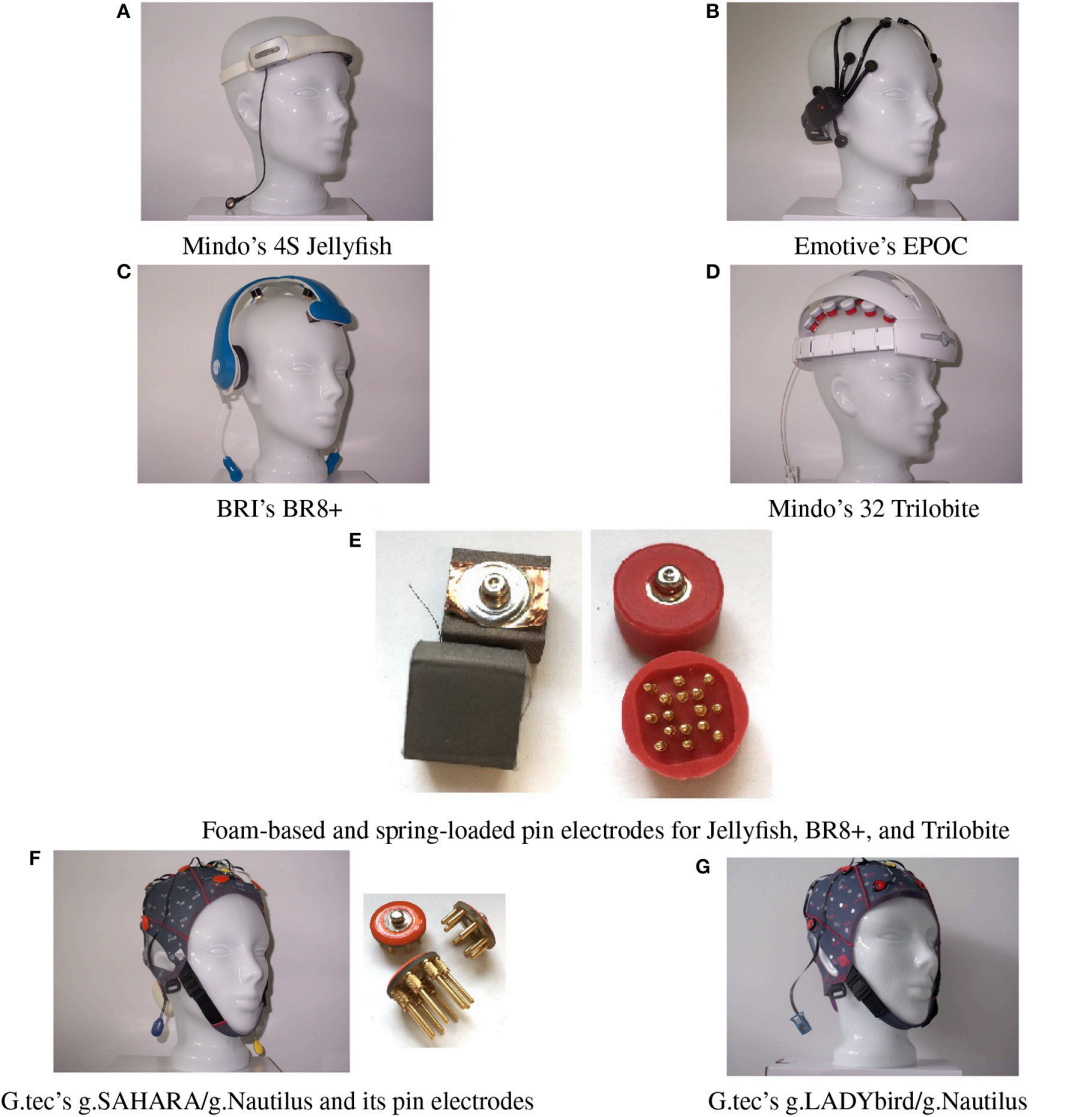
Six mobile EEG devices tested in our study.

The investigation focused on six mobile EEG devices. They are illustrated in Figure 1, and their specifications are summarized in Table 1.

**Table 1:** Technical data of tested EEG devices (n.s.: not specified).

**Device**	**EPOC**	**Trilobite**	**Jellyfish**	**BR8+**	**g.SAHARA**	**g.LADYbird**
Electrode type	Wet	Dry	Dry	Dry	Dry	Gel
	(saline)	(spring, foam)	(spring, foam)	(spring, foam)	(pins)	(active)
No. of channels	14	32	4	8	16	16
Battery life [hours]	12	10	10	11	10	10
Resolution [bit]	14	24	24	24	24	24
Max. sample rate [Hz]	128	500	500	1000	500	500
Bandwidth [Hz]	0.2–45	0.23–n.s.	0.23–n.s.	0.12–125	0.1–40	0.1–40
Weight [g]	116	524	95	269	233	165

The EPOC is the only device in our study that works with saline-based, wet felt sensors. It has two reference electrodes that are mounted at the parietal sides (P3/P4 locations).

The Jellyfish is also an easy-to-apply device. It consists of a headband with four dry electrodes and an adhesive reference electrode at the mastoid. The four electrodes can be applied at either frontal or parietal sites. The manufacturer recommends the use of foam-based electrodes for the frontal sites and spring-loaded electrodes for the parietal sites (Figure 1E). In our study, we registered the frontal EEG and thus attached foam-based electrodes to the headband.

The Trilobite device comes from the same manufacturer as the Jellyfish. It includes three foam-based frontal electrodes and 29 spring-loaded pin electrodes. Additionally, the device has a ground electrode and reference ear-clip electrode.

The BR8+ device comprises two frontal foam-based electrodes and six spring-loaded pin electrodes. Ground and reference electrodes are applied with ear-clips. The ear pads of the device do not have any technical functionality.

The pin electrodes of g.tec's g.SAHARA/g.Nautilus device are mounted on a traditional EEG cap. Adhesive ground and reference electrodes are applied at the mastoids. The cap of the device comes in small, medium, and large sizes. We only employed the medium-size cap in order to reduce the financial cost.

Finally, we also included a traditional, gel-based but mobile EEG system, the g.LADYbird/g.Nautilus device by g.tec. It includes 16 active electrodes and an ear-clip electrode as a reference. Although the cap size can vary, just as with the g.SAHARA/g.Nautilus device, we only used the medium-size cap in our study to reduce the cost. The g.LADYbird/g.Nautilus device was primarily developed for research and medical use. We included it to our study as a state-of-the-art reference for EEG registration in relation to the signal quality.

It was not possible to use the same sample rate for every device. In order to maintain comparable conditions for the later evaluation, we attempted to operate the devices with sample rates that were as similar as possible. Hence, for the Jellyfish and Trilobite devices, the EEG was registered at 256 Hz, and the g.SAHARA and g.LADYbird devices used 250 Hz. For both of the remaining devices, manual adjustment of the sample rate to 250 Hz was not possible. Thus, we had to run the EPOC device at 128 Hz and the BR8+ device at 1000 Hz. Furthermore, we applied a digital notch filter at 50 Hz during all of the recordings. All of the EEG devices utilized wireless signal transmission to a computer.

### 2.2. Procedure and subjects

Our study was conducted in a non-shielded office setting. Twenty-four subjects (11 females and 13 males, 26–66 years of age, with a mean age of 42.8) participated in the study. They tested one device per day for 60 min. During this time, the participants played computer games and performed one easy and one more demanding cognitive task for 5 min each. The 0-back task represented the easy task, where subjects were instructed to press the mouse button if the letter “X” appeared on the screen (Kirchner, 1958; Gazzaniga et al., 2013). The stop signal task was a more demanding inhibition task (Logan, 1994; Dimoska, 2005). During this task, the subjects were instructed to press the green mouse button as fast as possible if a horizontal left arrow was presented on the screen and the red mouse button if a horizontal right arrow appeared. If a horizontal arrow was quickly followed by a vertical arrow, they were instructed to inhibit their response and not press either button. They had to respond as quickly as possible and remember that their main aim was to keep the frame around the arrow green. A red frame meant that they were too slow. Hence, if it was red, they had to speed up their response while still paying attention to the vertical arrow.

Finally, we conducted two rest measurements, where we instructed the subjects to sit quietly for a minute, first with their eyes open and subsequently with their eyes closed. The devices were selected in random order over the participants and days, while the sequence of the performed tasks remained constant for all.

All of the investigations conducted were approved by the local review board of our institution, and the experiments were conducted in accordance with the Declaration of Helsinki. All of the procedures were carried out with the adequate understanding and written consent of the subjects.

## 3. Methods

To evaluate the signal quality, we examined the proportion of artifacts and signal-to-noise ratio of the devices in the time domain and considered the signal properties in the frequency domain.

### 3.1. Evaluation in time domain

Two hypotheses were postulated based on our expectations for the signal quality in regard to the time domain. In order to test both hypotheses, we employed EEG data from all of the computer tasks.

#### 3.1.1. Proportion of artifacts

*Hypothesis 1: The gel-based device has a significantly lower proportion of artifacts than the gel-free devices*.

The evaluation of the EEG in the time domain with regard to hypothesis 1 was conducted manually. The visual inspection and discarding of contaminated EEG segments by an expert is a widely applied and well-accepted method in research and clinical settings. Therefore, we asked for assistance from a medical technical assistant (MTA) with specialization in EEG analysis and years of experience in that field.

The MTA visually inspected the EEGs of each subject from all of the devices and manually marked artifact segments using a skill-based state-of-the-art procedure. Thereby, she did not mark physiological artifacts (e.g., eye blinks, eye movements) because these were not related to the device properties.

We then computed the percentage of denoted artifacts compared to the entire recording time for each channel. Finally, we calculated the means over the channels and subjects for each device.

#### 3.1.2. Signal-to-noise ratio

*Hypothesis 2: The gel-based device has a significantly higher signal-to-noise ratio than the gel-free devices*.

We computed the signal-to-noise ratio (SNR) as a standard method to assess the signal quality. The SNR values were calculated using the following relation:

(1) $$SNR=10\cdot \log_{10}\left(\frac{\sigma_{x}^{2}}{\sigma_{e}^{2}}\right)[\text{dB}]$$

where $\sigma_{x}^{2}$ is the variance of the signal, and $\sigma_{e}^{2}$ is the variance of the noise. For zero mean signals, as found here, this results in the following:

(2) $$SNR=10\cdot \log_{10}\frac{\sum_{\left(i=1\right)}^{N}x_{i}^{2}}{\sum_{\left(i=1\right)}^{N}{\left(s_{i}-x_{i}\right)}^{2}}$$

where *N* is the number of sample points, *x*_*i*_ is the noise reduced signal at time *i*, and *s*_*i*_ is the band-pass filtered signal at time *i*.

First, we filtered the original raw signals using a Hamming band-pass filter (order 100) between 1 and 40 Hz and obtained the filtered signal *s*_*i*_. Subsequently, we applied the artifact subspace reconstruction (ASR) algorithm to calculate the noise-reduced signal amplitudes *x*_*i*_ (Mullen et al., 2013). This algorithm is particularly suitable for cleaning continuous, non-triggered data from artifacts. Furthermore, the approach is well established within the scientific community (e.g., Bulea et al., 2015; Luu et al., 2017) and recommended for wireless, dry-electrode systems (Mullen et al., 2015). In the following, we give a brief description of how the algorithm works.

The algorithm identifies a clean signal segment from the given EEG and computes its statistics. Next, the ASR runs with a sliding window over the EEG and conducts a principal component analysis for each window. It removes high-variance components with three standard deviations above the mean and reconstructs their content using a mixing matrix calculated from the previously identified clean segment. For a more detailed explanation of the mathematical background and functionality of the algorithm, we advise the interested reader to consult the appropriate articles by the developers.

For the residual noise signal in the denominator, we used the difference between band-pass filtered signal *s*_*i*_ and the noise-reduced signal from the ASR algorithm, *x*_*i*_. The signal quality of the devices could be compared under this assumption. For each device, the SNR values were computed for all of the electrodes and subjects.

### 3.2. Evaluation in frequency domain

To evaluate the signal quality in the frequency domain, we formulated three more hypotheses. We expected that if a device had good signal quality, we would be able to measure significant differences in the signal's frequency band power values for the various tasks.

*Hypothesis 3: For devices with good signal quality, a significant Berger effect can be obtained between measurements with the eyes open and those with the eyes closed*.

Our third hypothesis was based on the so-called Berger effect (Berger, 1929). This states that the parietal alpha band power is supposed to be smaller with the eyes open than closed. This is also known as the “alpha block.”

**Figure 2:**
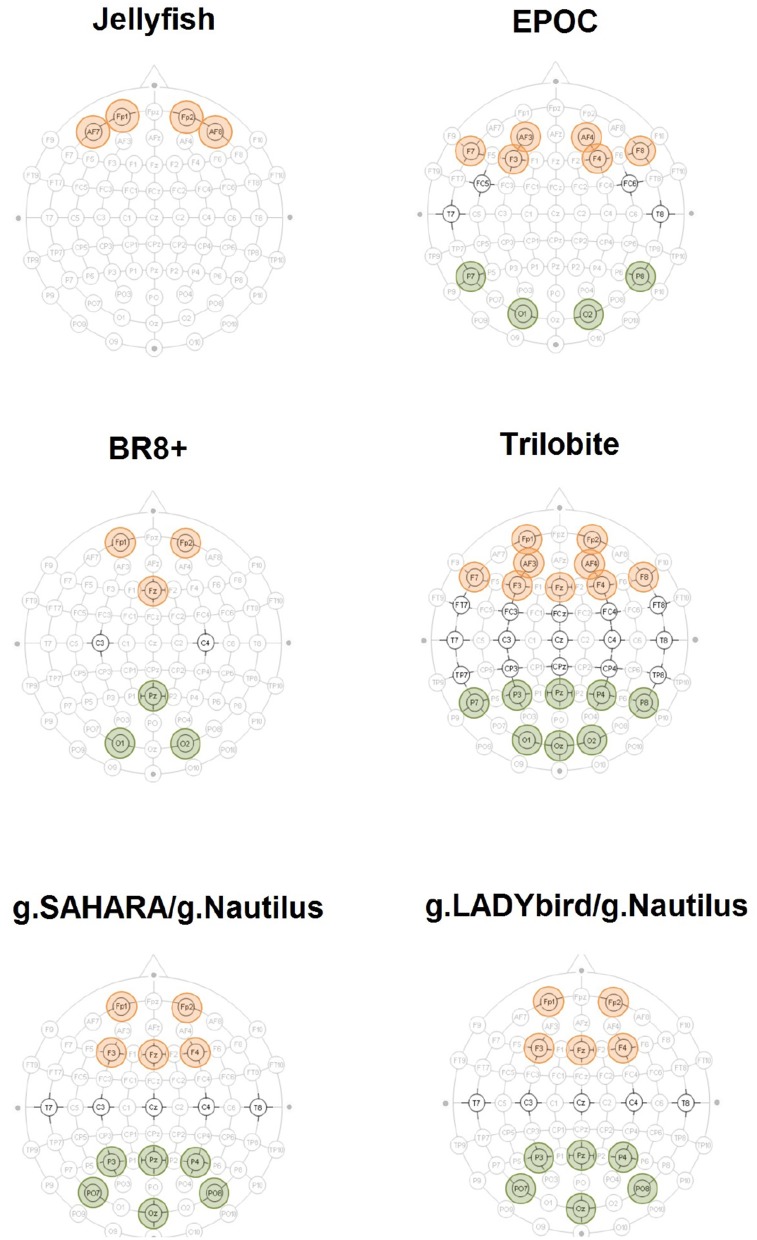
Accentuated positions constitute EEG devices' layout. The aggregated electrodes for the frontal theta-band power evaluation are highlighted in red. The electrodes used for the parietal alpha-band power calculation are highlighted in green.

For each device, we considered the two rest measurements with the eyes open and closed. We removed all of the segments previously marked as artifacts. We subsequently applied a Hamming band-pass filter for the alpha frequency band (8–12 Hz) to the artifact-free signals of the parietal electrodes (Figure 2). The relative band power values were averaged over the electrodes for the rest measurements with the eyes open and closed.

*Hypothesis 4: For devices with good signal quality, a significant increase in the frontal theta power can be obtained when comparing the easy and more demanding cognitive tasks*.

The fourth hypothesis was based on the dependency of the frontal theta band power on the experienced workload. Based on the results from numerous previous investigations (e.g., Gevins et al., 1998; Radüntz, 2016), we expected a significant increase in the frontal theta power when comparing the easy and more demanding cognitive tasks.

To this end, we focused on the EEGs from the 0-back and stop signal tasks of each device. First, we removed all of the previously marked artifact segments. We subsequently applied a Hamming band-pass filter for the theta frequency band (4–8 Hz) to the artifact-free signals of the frontal electrodes (Figure 2). The relative band power values were averaged over the electrodes for both the 0-back and stop signal tasks.

*Hypothesis 5: For devices with good signal quality, a significant decrease in the parietal alpha band power can be obtained when comparing the easy and more demanding cognitive tasks*.

Our last hypothesis was also based on findings regarding the experienced workload, but now with respect to the parietal alpha band power, which is expected to significantly decrease when comparing the easy and more demanding cognitive tasks (Gevins et al., 1998; Radüntz, 2016).

For each device, we considered the EEGs from the 0-back and stop signal tasks. We removed all of the previously marked artifact segments and applied a Hamming band-pass filter for the alpha frequency band (8–12 Hz) to the artifact-free signals of the parietal electrodes (Figure 2). Next, the relative band power values were averaged over the electrodes for both the 0-back and stop signal tasks.

## 4. Results

Digital signal processing was performed with MATLAB. All of the statistical calculations were carried out using SPSS. Furthermore, we provide Supplementary Material with the subjects' values for each analysis and system.

### 4.1. Evaluation in time domain

#### 4.1.1. Proportion of artifacts

To statistically evaluate the proportion of artifacts for the various devices, we conducted an analysis of variance (ANOVA) with a repeated measures design. The six devices constituted the levels used for testing each subject at each level of the within-subject variable. Bonferroni's corrected *post-hoc* tests were conducted to determine the differences between the levels.

**Figure 3:**
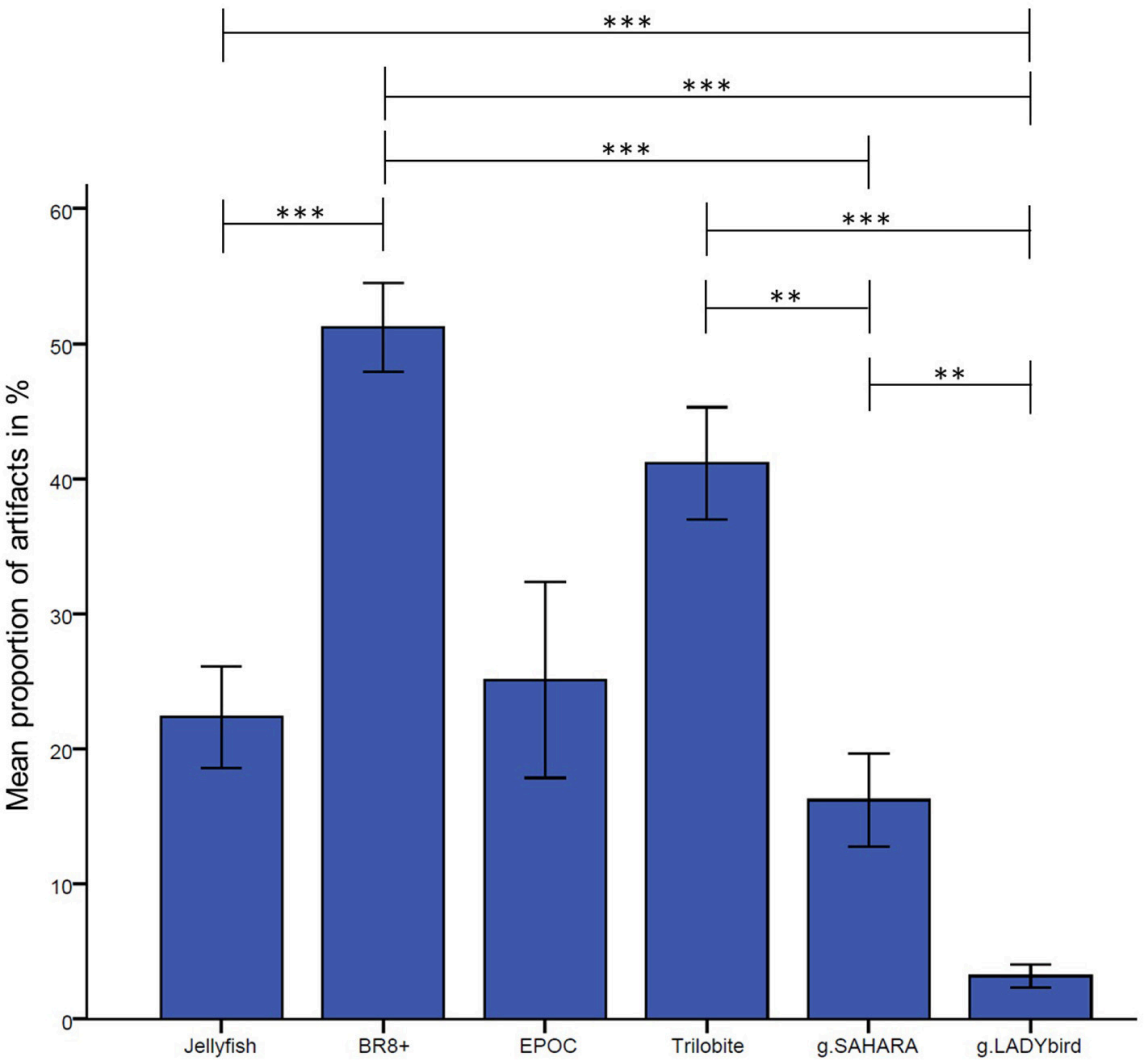
Proportion of manually tagged artifacts in EEG averaged over channels and subjects for each device (calculation of analysis of variance with repeated measures design and Bonferonni-corrected *post-hoc* tests: ^***^: *p* ≤ 0.001; ^**^: 0.001 < *p* ≤ 0.01; ^*^: 0.01 < *p* ≤ 0.05; error bars indicate ± one standard deviation).

The results are presented in Figure 3. They indicate significant differences among the devices in relation to their proportions of artifact-contaminated signal segments [Greenhouse-Geisser: *F*_(2.72; 62.61)_, = 15.88, *p* < 0.001]. The *post-hoc* tests showed that the traditional gel-based g.LADYbird device had significantly fewer artifacts than almost all of the other devices, and that the BR8+ device had significantly more artifacts than most of the others. The dry pin-electrode device (g.SAHARA) yielded a significantly lower artifact proportion than the remaining pin-electrode devices. However, it had a higher proportion than the gel-based device. Finally, no significant differences compared to any other device could be obtained for the EPOC device.

#### 4.1.2. Signal-to-noise ratio

Before going into detail about the SNR results, it should be noted that the ASR algorithm failed when examining the EEGs of four subjects that were recorded with the Trilobite device. This was because no segment of the needed length could be found as a reference for the algorithm, where all of the electrodes' signals were concurrently clean. Hence, these four subjects had to be excluded from the subsequent statistical computations for all the devices.

**Figure 4:**
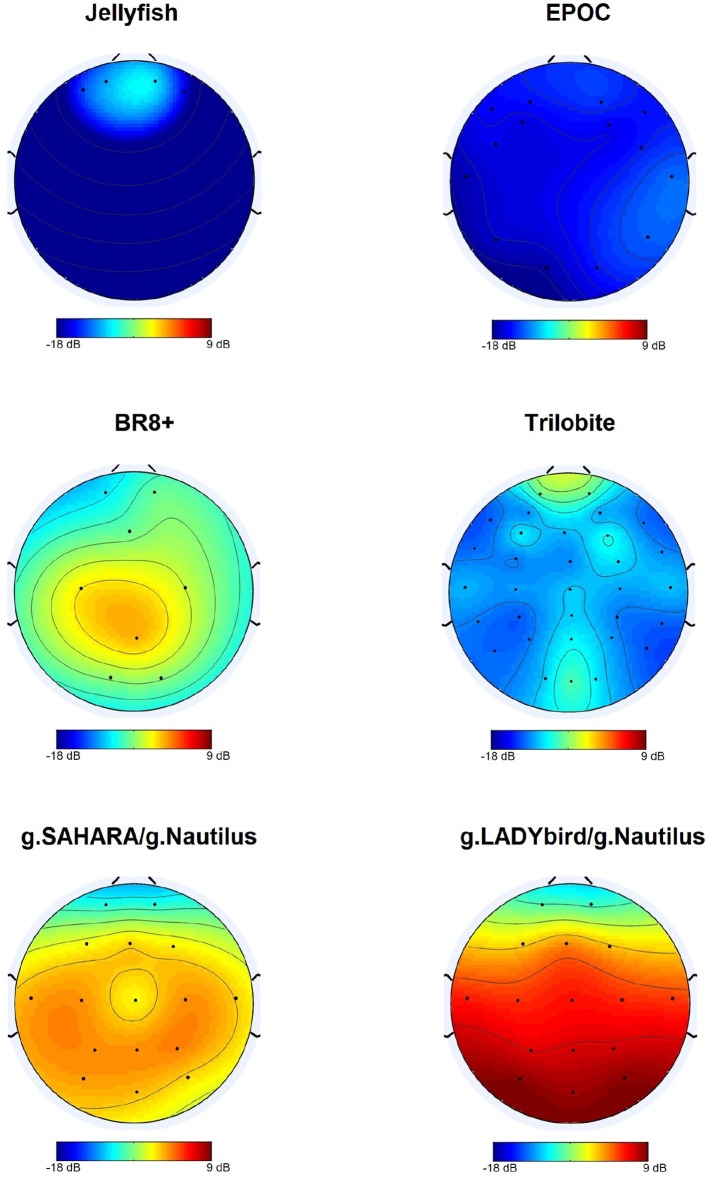
Median SNR values obtained over subjects for each channel.

For each device, we calculated the median of the SNR values for each electrode over all the subjects and tasks. At the first site, we found obvious differences among the devices and noticed that g.LADYbird and g.SAHARA had the highest SNR values (Figure 4). In order to statistically evaluate these observations, we calculated the median of the SNR values over all the channels for each subject and device. We then conducted a non-parametric Friedman test of the differences among the six devices.

**Figure 5:**
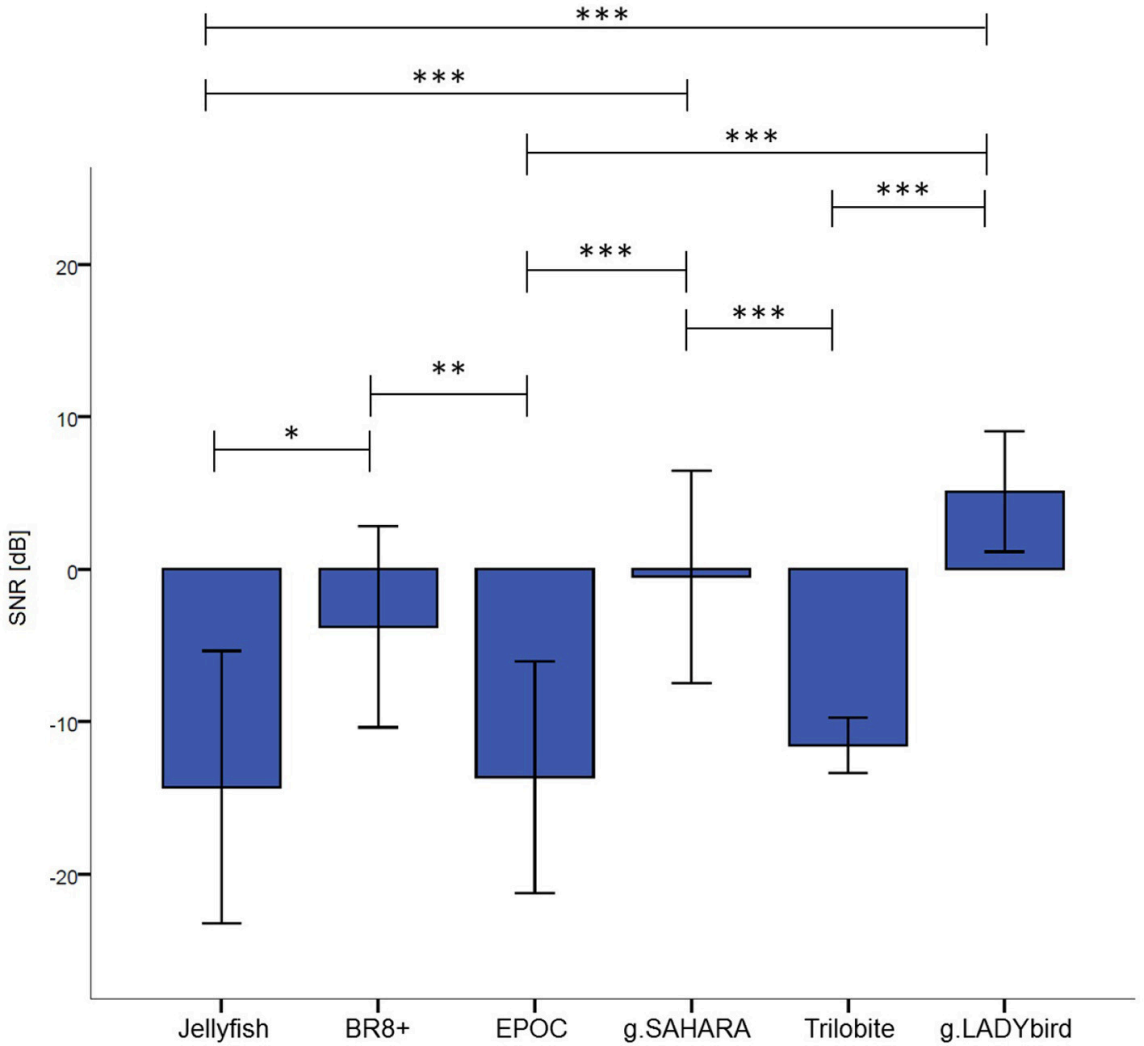
Median SNR values over channels and subjects for each device (calculation of Friedman test of differences and Bonferonni corrected *post-hoc* tests: ^***^: *p* ≤ 0.001; ^**^: 0.001 < *p* ≤ 0.01; ^*^: 0.01 < *p* ≤ 0.05; error bars indicate ± one standard deviation).

The results indicated significant differences in the devices' SNR values (χ^2^ = 71.34, *df* = 5, *n* = 20, *p* < 0.001). Dunn-Bonferroni *post-hoc* tests were conducted to determine the differences between the devices. The results are presented in Figure 5. The g.LADYbird device yielded significantly higher SNR values than the Trilobite (*z* = −5.409, *p* < 0.001, *r* = 1.2), EPOC (*z* = −6.339, *p* < 0.001, *r* = 1.4), and Jellyfish devices (*z* = −5.832, *p* < 0.001, *r* = 1.3). The g.SAHARA showed results that were similar to those of g.LADYbird for these three devices (Trilobite: *z* = 4.226, *p* < 0.001, *r* = 0.9; EPOC: *z* = −5.155, *p* < 0.001, *r* = 1.2; Jellyfish: *z* = −4.648, *p* < 0.001, *r* = 1.04). Furthermore, the BR8+ device showed significantly higher SNR values than the EPOC (*z* = 3.803, *p* < 0.01, *r* = 0.9) and Jellyfish devices (*z* = −3.296, *p* < 0.05, *r* = 0.7). All of the obtained effect sizes for the previously mentioned correlation coefficients for device pairs could be interpreted as large according to the guidelines of Cohen (1992).

### 4.2. Evaluation in frequency domain

To evaluate the signal quality in the frequency domain, we conducted a statistical test for each hypothesis. A separate statistical inference evaluation was performed for each device because of the substantial differences between the devices. These arose from the different numbers of electrodes, different electrode layouts, different reference electrodes, and different electrode types. Although those differences did not allow for a statistical inference analysis among the devices, determining a separate inferential statistic for each device seemed to be appropriate to test the hypotheses. The results for the devices could only be compared descriptively. Furthermore, it should be mentioned that evaluations of the third and fifth hypotheses were not possible for the Jellyfish device because of its electrode configuration.

**Figure 6:**
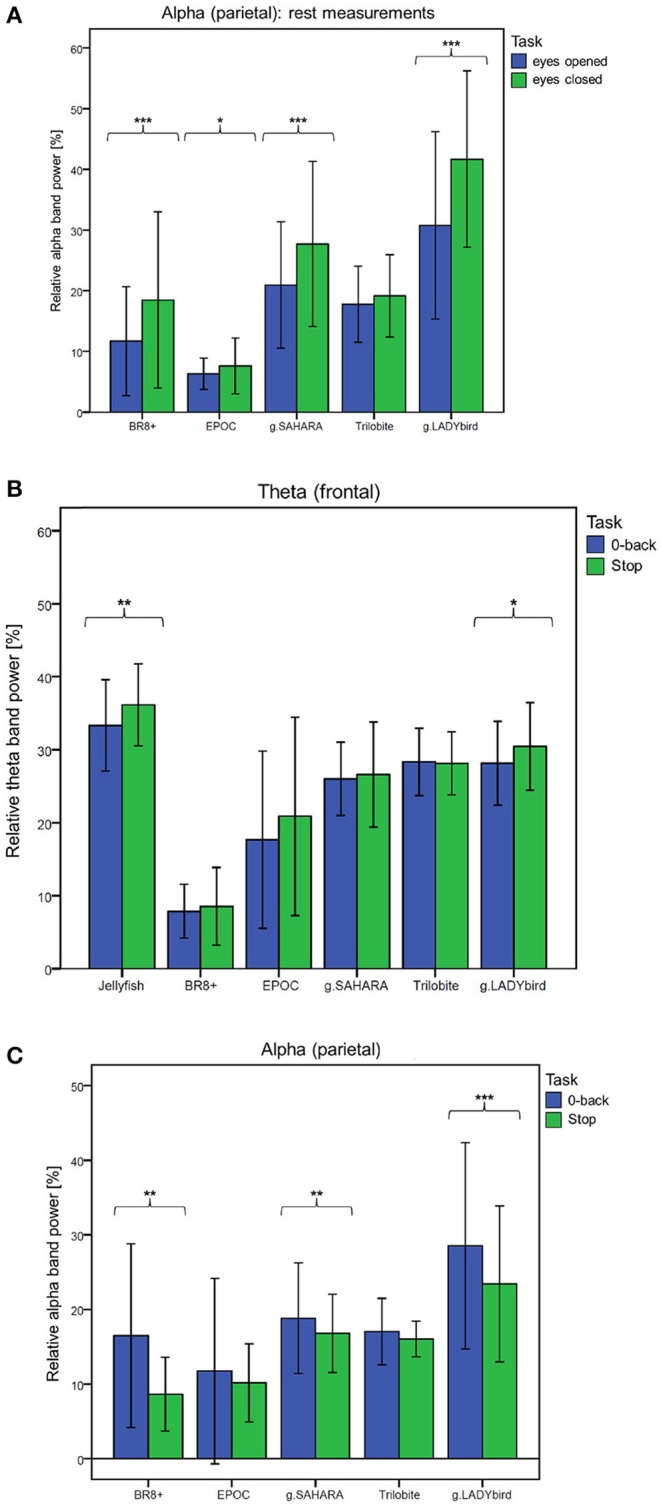
Frequency band differences in respect to different conditions averaged over channels and subjects and considered for each device separately (calculation of Wilcoxon paired difference test for not-normally distributed data; ^***^: *p* ≤ 0.001; ^**^: 0.001 < *p* ≤ 0.01; ^*^: 0.01 < *p* ≤ 0.05; error bars indicate ± one standard deviation). **(A)** Hypothesis 1: Behavior of parietal alpha band power during rest measurements with eyes open and eyes closed. **(B)** Hypothesis 2: Behavior of frontal theta band power during easy and more demanding cognitive tasks. **(C)** Hypothesis 3: Behavior of parietal alpha band power during easy and more demanding cognitive tasks.

For the third hypothesis, we considered the parietal alpha band power values of the rest measurements with the eyes open and closed. We used the Shapiro-Wilk test to assess whether the alpha band power values of these two rest measurements were normally distributed for each device. This was not the case for the eyes-open parietal alpha band power values of all the devices (*p* < 0.05). Similarly, the alpha band power with the eyes closed was not normally distributed for most of the devices, with the exception of g.SAHARA and g.LADYbird (*p* > 0.05). Hence, for comparison purposes, we conducted a Wilcoxon paired difference test for each EEG system. The results are presented in Figure 6A. They show significant differences in the alpha frequency band power values between the eyes open and eyes closed for all of the devices except the Trilobite device (*p* = 0.19).

We used a similar procedure for the fourth hypothesis. Hereby, the theta band power values of the 0-back and stop signal tasks were considered. For all of the devices, the theta band power of the 0-back task was approximately normally distributed, whereas that of the stop signal task was not, as assessed by the Shapiro-Wilk test (Jellyfish and BR8+ with *p* < 0.05). Hence, a Wilcoxon test was conducted. Figure 6B shows the results. A significant increase in the frontal theta band power between the easy and more demanding tasks could only be obtained for the Jellyfish and g.LADYbird devices.

Finally, in order to prove our last hypothesis, we examined the alpha band power values of the two cognitive tasks. The Shapiro-Wilk test indicated that during the 0-back task, the alpha band power was not normally distributed for any device (*p* < 0.05). During the stop signal task, the alpha band power was normally distributed for almost all of the devices except the EPOC and g.LADYbird (*p* < 0.05). Thus, a Wilcoxon test had to be applied. The paired difference test between the easy and demanding tasks yielded significant decreases in the parietal alpha band power values for the BR8+, g.SAHARA, and g.LADYbird devices (Figure 6C).

## 5. Discussion and conclusion

A visual examination of the signals in the time domain and statistical analysis of their proportions of artifacts showed that the gel-based g.LADYbird device had the fewest disturbances, as postulated by hypothesis 1. Among the gel-free devices, the g.SAHARA device had the best performance, with only a small percentage of artifact-contaminated segments. We also want to remind the reader that no significant differences at all could be identified for the EPOC device. This was probably due to the high variance among the subjects and requires a discussion to provide useful information for the use of this device. It is a fact that the headset did not provide a good fit for the various head sizes of the subjects. In these cases, the electrodes did not make good contact with the skin, and the recorded signals included noise interference at 23 and 28 Hz. We assumed that in the case of loose electrode contact, the device caused aliasing artifacts from the electrical mains. Thus, we contacted the manufacturer for a detailed explanation. Their technical support stated that “the problem arises because the common mode sense active electrode and driven right leg passive electrode pair cannot cancel the ambient noise, either because the headset is not on a human, or because the connections at the reference locations (behind and 30° above the ears, or directly behind each ear) are not making good contact.” We concluded that the variance in the artifact proportions among the subjects was large because of the difficulty of adapting the device to the different head sizes. However, the EPOC device is only manufactured in one size, which leads to bad outcomes regarding the signal quality.

For our second hypothesis, we used the signal-to-noise ratio as a criterion to characterize the signal quality of the devices. For all of the devices, the obtained SNR range was quite low, from −18 to 9 dB, and within the range found in the literature. As expected, the SNRs were lower in the frontal areas, which were contaminated by eye artifacts (Goldenholz et al., 2009; Mishra and Singla, 2013; Radüntz et al., 2015). The gel-based g.LADYbird device yielded the best SNR value. A statistical analysis showed that it was significantly higher than the three poorest SNRs of the Trilobite, EPOC, and Jellyfish devices. Among the gel-free devices, we obtained the best SNR value for g.SAHARA. Similar to the values of the g.LADYbird device, g.SAHARA's SNR was significantly higher than the SNR values of the Trilobite, EPOC, and Jellyfish devices. However, remarkably, and in contrast to the g.LADYbird device, none of the gel-free devices could yield SNR values greater than 0 dB (Figure 5). This indicated that the ratio between the signal and noise was smaller than one. The noise was superimposed on the signal, which could prove to be particularly problematic in clinical practice, where precise measurements are required.

Our first two hypotheses concentrated on evaluating the EEGs in the time domain. While this evaluation aimed at the first instance to identify the very obvious differences regarding the devices' artifact susceptibility, our evaluation in the frequency domain went a step further. After removing all of the artifact-contaminated segments, we wanted to look deeper at the signal and determine whether it reflected the actual brain activity. For this, we postulated three additional hypotheses based on the well-studied behavior of the EEG. If the devices effectively recorded a brain signal, the Berger effect had to be clearly noticeable. Furthermore, as task demands became greater, we expected an increase in the frontal theta frequency band power and a decrease in the parietal alpha frequency band power.

Significantly, for the gel-based g.LADYbird device, all three frequency-domain hypotheses were proven to be true. For the g.SAHARA and BR8+ devices, significant differences could be obtained regarding the Berger effect and decrease in the parietal alpha band power during the demanding cognitive task. The EPOC device yielded significant differences only for the Berger effect. The Jellyfish device was included only in the examination of the frontal theta band power behavior. It was the only device among the gel-free devices that was able to register a significant increase in the theta band power as task demands increased. Only one device did not show any significant changes in the signal's band power in reference to any of our last three hypotheses: the Trilobite device.

To conclude, all of the devices tested are mobile and do not limit a subject's mobility. All of the devices, except the g.LADYbird device, are easily applicable by the subjects themselves because of their gel-free electrodes. The signal quality results yielded by this study are summarized in Table 2. In order to provide useful information to practical users of EEG devices, in the following, we indicate which system could be used under which condition.

**Table 2:** Signal quality results of tested EEG devices (^***^: *p* ≤ 0.001; ^**^: 0.001 < *p* ≤ 0.01; ^*^: 0.01 < *p* ≤ 0.05).

**Device**	**EPOC**	**Trilobite**	**Jellyfish**	**BR8+**	**g.SAHARA**	**g.LADYbird**
Proportion of artifacts [%]	25.11	41.14	22.36	51.22	16.21	3.19
SNR [dB]	−13.66	−11.55	−14.31	−3.78	−0.50	5.09
Berger effect	^*^		−	^***^	^***^	^***^
Increase in frontal theta			^**^			^*^
Decrease in parietal alpha				^**^	^**^	^***^

Outstanding performances were obtained for the traditional gel-based but mobile g.LADYbird/g.Nautilus device. None of the other emerging devices could reach its signal quality. This device can be recommended for neuroscience research where precise measurements are required.

The signal quality of the g.SAHARA/g.Nautilus device was the best among the gel-free devices and could be considered quite satisfactory. The g.SAHARA/g.Nautilus seems to be a good solution for conducting field experiments. A potential issue could be user acceptance because of the not very flattering cap design and its comfort. A long wearing time for the pin electrodes could be a major problem. Within the framework of our study, we used several questionnaires regarding user experience. The obtained results will be presented in a following paper.

The remaining devices did not meet our requirement of an appropriate signal quality, although some readers could decide to use them for mobile applications.

The EPOC and BR8+ devices suffered from a large proportion of artifacts caused by a poor fit, depending on the subject's head size and form. Hence, they can only be recommended for use if they are guaranteed to perfectly fit the subject's head, e.g., personalized brain-computer applications.

Potential users of the Jellyfish device should be aware that the device only measures the frontal brain activity. In addition, the signal of the frontal electrodes is contaminated by a large number of artifacts. Furthermore, the small number of electrodes does not facilitate the application of artifact-correction algorithms that employ ambient information. However, potential applications suitable for this device could be located in the gaming or bio-feedback sector.

Finally, the results of the Trilobite device were unsatisfactory. This was because of the negative evaluations in both the time domain and frequency domain. A recommendation for the use of the Trilobite device cannot be given based on the obtained results.

It has to be mentioned that the EEG equipment market shows rapid development. During this study, new devices appeared on the market that could not be tested, e.g., the actiCAP Xpress Twist/LiveAmp device by BrainProducts. Furthermore, there is a new highly innovative approach using in-ear EEG technology (Looney et al., 2012; Goverdovsky et al., 2017).

For triggered data from event-related potentials, Oliveira et al. (2016) have already proposed metrics for evaluating new EEG technologies. However, our study design and the proposed method for evaluating the signal quality of devices could easily be used in subsequent studies of new devices and continuous data without triggers. Such a benchmark would allow for the evaluation of further emerging EEG technology and the integration of the test results from new devices into the findings already in existence. This would make it possible to compare emerging EEG devices.

## Author contributions

TR initiated the project and was responsible for the overall conception of the investigation and the data analysis. Data interpretation was performed by TR. The manuscript was written by TR.

### Conflict of interest statement

The author declares that the research was conducted in the absence of any commercial or financial relationships that could be construed as a potential conflict of interest.
