# Supplementary material for: Signal Quality Evaluation of Emerging EEG Devices
Source: Front Physiol. 2018 Feb 14;9:98. doi: 10.3389/fphys.2018.00098 (PMC5817086; doi:10.3389/fphys.2018.00098)
Supplement: Supplementary file 1 [file DataSheet1.ZIP › SNR_Trilobite.pdf]

Trilobite (all tasks)

SNR [dB]

Vp	Fp2	Fp1	AF4	AF3	F8	F4	Fz	F3	F7	FT8	FC4
11	-7.83365202	-6.47225523	-16.4812546	-15.1507664	-10.3962421	-10.0218477	-16.3330536	-8.22752857	-14.5082445	-17.4669628	-9.28985596
12	-3.51140714	-4.81096745	-9.75910091	-10.862958	-11.1728716	-8.14314461	-16.3819561	-5.43800068	-9.63697815	-11.5208464	-7.88668633
13	-7.9099865	-6.97402573	-7.1533823	-5.13695288	-10.8667049	-8.17712975	-6.79062033	-10.7717781	-15.2648964	-8.08274651	-9.7855463
14	0.09573147	-3.10043979	-13.3958464	-16.7952671	-13.0666008	-8.16183567	-13.1346054	-13.5934248	-12.9697962	-14.7307987	-11.0635309
15	-6.638659	-7.35663605	-17.0025177	-14.7821579	-17.2758045	-7.70612335	-11.51929	-19.6923332	-12.7742271	-18.274725	-8.96222878
17	-7.94554853	-11.1239061	-12.031744	-12.8126879	-19.0449238	-6.01007795	-11.0177164	-5.94672489	-17.5899181	-18.9581013	-9.06015778
18	-14.524785	-10.3674431	-11.6683826	-13.7807121	-10.4853868	-30.5548611	-13.1869459	-30.4999733	-6.74642467	-15.3336649	-18.759613
19	-9.81309795	-7.6278019	-10.4850054	-7.91546059	-4.48432302	-6.7920084	-11.2554989	-6.13866472	-14.0854864	-8.01048088	-9.01411819
20	-6.89356947	-7.24871302	-10.5123167	-10.6045904	-10.4826975	-8.95375156	-8.50650024	-6.90064573	-13.5615807	-8.04177284	-8.90895748
21	-6.07002258	-8.36874294	-13.099782	-11.9477978	-14.9500093	-10.647728	-10.0028057	-9.2281723	-14.8306475	-14.445775	-12.5239973
22	-5.06424284	-4.30541945	-11.616086	-17.3911705	-13.5752563	-11.558589	-16.8308487	-16.4642048	-12.6190481	-10.0252266	-8.62376595
23	-2.78704691	-3.33189201	-8.93942165	-5.29657412	-12.8994341	-6.62537336	-8.60368252	-13.4308653	-8.44043922	-13.1446018	-11.0629196
24	-4.63518381	-5.2424736	-13.7224035	-14.8418989	-14.1140337	-7.26363754	-10.475688	-15.3298254	-15.7494936	-11.0247726	-10.0094051
25	-4.86068773	-5.25521326	-11.5807123	-8.00333214	-11.5048618	-9.69296551	-8.46864033	-8.96677399	-12.3444052	-11.4451904	-9.86599827
26	-4.43788147	-3.6148479	-6.56944466	-14.9289646	-11.2826271	-11.9035034	-13.8418627	-14.8699408	-17.0395622	-15.4525528	-13.1955652
28	-12.1540956	-11.0182924	-10.5197515	-12.7944088	-14.0817461	-11.240942	-11.9561691	-13.4786491	-11.3345308	-15.4809341	-9.11260223
30	-9.78335667	-15.3133287	-16.2734947	-17.3226852	-10.1045237	-9.64521694	-18.0828438	-7.93822289	-9.06477642	-18.5110455	-9.04518986
32	-14.2163153	-3.08326173	-4.80448818	-1.45810354	-14.2720184	-6.35865974	-6.01377821	-4.73741007	-13.2097473	-6.17568302	-11.7317877
33	-1.73365331	-2.10548282	-16.2890778	-2.44048762	-27.6831722	-1.72955739	-2.57707453	-18.0096989	-16.5110073	-11.9936714	-13.8199415
34	-9.76325989	-9.08705711	-11.4396324	-13.7656822	-14.3396368	-4.34294033	-10.8189001	-6.76898861	-16.027483	-9.20085239	-10.1209459

Vp	FCz	FC3	FT7	T8	C4	Cz	C3	T7	TP8	CP4	CPz	CP3
11	-19.3391075	-5.54660892	-12.4407091	-19.8857822	-12.7686396	-19.4346027	-11.3173265	-12.7047863	-4.05980349	-15.4794245	-10.3203278	-19.9942913
12	-16.8871517	-5.08948231	-15.6753502	-18.4437561	-14.0530062	-8.96837997	-15.6715603	-14.5457973	-10.0625954	-17.2115822	-7.67919397	-13.8643026
13	-10.3127174	-13.4233294	-15.3841972	-9.62210655	-7.79877949	-13.7165737	-12.5202065	-12.7959337	-16.2297096	-13.5687971	-12.4204702	-14.0799084
14	-12.0426588	-13.8453922	-11.4275703	-13.9102392	-12.8333511	-12.6697721	-10.9669352	-5.61155367	-14.4186792	-11.5215082	-11.6199026	-8.80284691
15	-11.5675821	-6.12149715	-16.1065712	-18.307476	-8.82346725	-10.7230301	-11.271553	-9.59088993	-12.4086456	-7.51484108	-8.98310661	-15.6352692
17	-18.6736984	-5.85658979	-7.05196428	-21.8672314	-16.5797195	-13.9709721	-13.9787197	-13.4304028	-20.753973	-7.66436863	-9.53992271	-19.8598537
18	-10.8586559	-4.43816376	-7.83460426	-12.4282446	-12.2382154	-7.28449535	-11.5309801	-4.38349533	-13.9371614	-8.25388813	-7.35344076	-13.992363
19	-10.8872976	-13.5668669	-13.8749447	-12.9340315	-14.6306114	-9.9601202	-10.4523048	-14.8275461	-14.7316904	-12.1116505	-9.93283463	-12.8916311
20	-10.7169123	-13.8050508	-11.9667902	-8.24196625	-6.32644415	-8.33427525	-12.2775974	-11.976491	-14.2778816	-13.8615198	-11.8131733	-13.9119549
21	-14.409831	-15.4676771	-9.98948002	-9.28853035	-15.572258	-15.231595	-16.1657658	-17.7307873	-17.7206783	-30.3922291	-14.7489042	-16.3189735
22	-7.39950323	-12.6888199	-14.1641483	-11.9894323	-13.4612856	-6.00464058	-11.1719227	-12.585887	-7.32189703	-11.4461012	-8.78564167	-11.2531633
23	-12.7756319	-13.7601442	-8.84579754	-13.4929962	-14.2375698	-10.0047913	-13.8508091	-9.27089024	-15.2175531	-13.0613518	-11.7068529	-13.5063581
24	-13.601512	-15.0090008	-14.4295883	-10.7013655	-8.29561138	-12.4935303	-8.13219261	-14.239028	-15.2985754	-3.57548022	-11.643342	-11.4857893
25	-9.48409653	-13.7924232	-13.8401937	-6.48486948	-13.1715336	-7.75517988	-13.221488	-14.7393951	-14.302742	-13.7340841	-11.3427839	-13.9676371
26	-15.5638437	-14.3760567	-18.7262802	-10.2766228	-11.6440201	-9.07699299	-17.808897	-14.3998032	-10.6592636	-8.55755424	-11.9114304	-15.9431047
28	-14.7118616	-6.88556528	-12.7794104	-10.2629337	-8.3506155	-12.5774975	-9.52590752	-10.4546232	-14.0223989	-12.3209915	-11.9092569	-9.67386341
30	-16.493309	-9.10536671	-8.05531597	-16.207552	-9.1407423	-16.0371532	-16.4852142	-6.40364361	-9.66732788	-16.2201614	-11.011941	-16.6475258
32	-20.3514824	-7.58375216	-8.97799301	-6.56191111	-11.6819077	-6.78774261	-4.81367636	-8.3013792	-12.9743996	-14.7011766	-5.53624725	-4.78941298
33	-3.65491199	-29.9337006	-16.4573517	-7.38677359	-13.8511362	-3.65045428	-8.30626965	-2.59814715	-9.83742523	-20.6177597	-2.75333405	-8.51809883
34	-12.5623674	-12.6161489	-14.4435663	-9.5209856	-11.8797636	-15.5026827	-11.0557356	-4.50192165	-13.7307329	-4.81929684	-10.7155647	-8.22753048

Vp	TP7	P8	P4	Pz	P3	P7	O2	Oz	O1	mean	median	std
11	-13.1803379	-13.0619516	-14.293767	-7.6045928	-10.8339128	-14.2484293	-16.9373569	-17.7966824	-18.1969585	-13.1758457	-13.1211448	4.41110257
12	-8.29639912	-17.1176891	-10.8620796	-6.84088755	-9.98054695	-15.8374243	-13.6261406	-8.58032227	-15.3176746	-11.3667575	-10.8625188	4.1846058
13	-14.1215611	-12.5499144	-14.2265348	-11.3463621	-13.7440405	-14.4573736	-13.8411074	-13.0305061	-14.8135777	-11.5911711	-12.5350604	3.01959191
14	-9.39030552	-20.9722939	-4.31603527	-8.92726707	-9.42819786	-16.9507122	-8.2571497	-5.93065643	-11.1327019	-11.0903795	-11.5707054	4.24384211
15	-12.4194212	-15.6738148	-10.788147	-8.6585083	-14.0139208	-15.7707748	-12.480176	-9.64655113	-15.9537067	-12.3263641	-11.9881139	3.82085431
17	-16.6316681	-11.64711	-7.18102932	-8.04586697	-10.8398533	-16.7471428	-17.4133701	-5.96149254	-19.3346291	-12.9553464	-12.4222159	5.08688562
18	-11.3315849	-2.52184606	-20.2077904	-6.44224453	-25.9089394	-10.3108931	-11.8338556	-9.80943489	-12.5445499	-12.5422825	-11.5996814	6.64154027
19	-14.2220297	-14.3861799	-12.1388025	-9.0369072	-13.0300531	-9.84506989	-9.29810047	-4.24812269	-9.30353451	-10.6853836	-10.4686551	2.99012836
20	-13.9591093	-14.1398897	-12.658021	-9.991436	-13.3492918	-10.7303038	-7.20288515	-7.23187113	-6.773633	-10.4425498	-10.5584536	2.65850542
21	-17.8540058	-18.7022114	-17.7373028	-13.7280455	-15.3180809	-17.3185825	-9.36907578	-12.2980766	-8.81415939	-14.0716166	-14.5973396	4.47245745
22	-11.8054676	-10.5595875	-8.24568558	-8.72515011	-10.8591175	-8.84495068	-7.8062849	-6.00917578	-6.5256381	-10.49148	-11.0155201	3.3383123
23	-14.4859991	-14.0460186	-11.2756023	-11.3366833	-12.6935463	-13.008215	-6.73421907	-5.40986967	-9.76841164	-10.7203613	-11.5217681	3.40111463
24	-11.6597271	-16.0434914	-12.4474945	-10.7697897	-14.4120483	-10.0057983	-9.02800083	-7.42856455	-11.8165894	-11.4039167	-11.6515346	3.36966657
25	-14.2608089	-14.4938307	-12.74016	-9.80264759	-13.7183161	-11.2703533	-5.94203091	-6.12247133	-7.03158855	-10.7252317	-11.3939872	3.07590024
26	-10.4155445	-18.6380978	-13.0204973	-11.5019894	-16.6175556	-12.7189817	-13.2348518	-3.81633353	-14.2915926	-12.5105021	-13.1080313	3.99804128
28	-12.956708	-8.2958765	-6.9473772	-9.97782803	-10.2249832	-6.32687521	-7.10518932	-5.12582016	-13.1157484	-10.8351079	-11.1296172	2.59862625
30	-9.48532295	-16.2012157	-17.1739502	-8.16856575	-13.8443432	-16.4662495	-15.75424	-6.90654612	-14.8734293	-12.8573063	-14.3588862	3.94306173
32	-8.54975891	-6.9118886	-2.5093441	-2.66076374	-7.25777149	-8.83680916	-6.83325005	-3.78391171	-2.70243216	-7.78650825	-6.81049633	4.36640978
33	-2.31508756	-5.61258411	-9.11425018	-5.6955452	-7.87169266	-21.4406738	-5.54073811	-9.17924404	-5.72765255	-9.84236425	-8.08898115	7.67512997
34	-13.0034056	-14.873291	-13.5553675	-10.7310247	-13.9225082	-12.9036875	-11.8656559	-10.2190866	-9.66162682	-11.124604	-11.247684	3.06887353
