# Supplementary material for: Signal Quality Evaluation of Emerging EEG Devices
Source: Front Physiol. 2018 Feb 14;9:98. doi: 10.3389/fphys.2018.00098 (PMC5817086; doi:10.3389/fphys.2018.00098)
Supplement: Supplementary file 1 [file DataSheet1.ZIP › A-proportion_BR8+.pdf]

BR8+ (all tasks)
Proportion of artifacts [%]

Vp	Fp1	Fp2	Fz	C3	C4	Pz	O1	O2	mean	median	std
11	84.0673895	15.8002789	18.9794466	99.9806114	99.9756666	99.9806114	49.2679027	52.5859994	65.0797383	68.3266944	35.805443
12	9.16584849	9.79448858	64.4694964	12.7005191	18.6248931	68.443449	9.83170713	12.1933696	25.6529714	12.4469443	25.3819325
13	99.9823785	99.9836246	5.58803264	99.9823785	99.9823785	80.5520944	26.2441552	27.8674761	67.5228148	90.2672365	40.5264059
14	65.0611247	11.0070271	14.843504	25.9159865	99.9939404	99.9949313	28.6975044	51.1985747	49.5890741	39.9480395	35.8888713
15	99.3500419	4.13918739	3.77186806	99.8920958	74.6705134	95.3090633	37.8344683	32.4997572	55.9333744	56.2524909	41.4023237
16	59.9205724	2.50381911	4.07938711	99.9914104	99.9910557	99.9910557	99.9910557	99.9910557	70.8074265	99.9910557	43.9073365
17	36.4240748	25.5989793	24.2708305	35.1571785	99.9908934	36.9694661	26.0772196	23.9224091	38.5513814	30.6171991	25.452119
18	84.1848613	9.79429349	7.39765787	28.0453772	17.5149665	99.9938256	9.19856976	9.04238775	33.1464924	13.65463	37.2380097
19	47.1328623	17.5447689	14.7895993	25.9104824	23.287637	99.9936652	13.9250407	14.5442328	32.1410361	20.416203	29.5236708
20	76.9546635	18.406145	12.4952609	79.9752931	79.9752931	10.8305584	7.89584966	23.267127	38.7250238	20.836636	33.6643471
21	79.9895888	79.9134089	4.07628643	79.9895888	4.25941614	5.07454843	2.92186676	3.16315623	32.4234826	4.66698229	39.3729719
22	10.6152567	10.6725721	10.2320021	79.9782966	79.9782966	10.0552443	10.6265784	10.7008565	27.8573879	10.6495753	32.1705392
23	13.7109053	8.63715464	6.93870428	6.74096364	99.9791521	99.9791521	99.9791521	99.9791521	54.493042	56.8450287	48.6732046
24	29.0134254	11.9926049	8.80509553	99.8169404	11.5439157	99.9928588	99.9928588	99.9928588	57.6438198	64.4151829	45.6274517
25	76.8162623	43.3582582	36.2414809	99.9940876	99.9940876	99.9940876	99.9940876	99.9940876	82.0483049	99.9940876	27.3412684
26	93.3762454	12.9780564	30.2992672	99.9955025	99.9955025	64.5621422	30.4953875	30.8213846	57.815436	47.6917634	36.0772965
27	14.0245463	19.1966415	13.7654385	71.4971348	36.3206268	99.9863662	49.5873959	48.7572714	44.1419277	42.5389491	30.3105144
28	4.33710646	4.6086857	4.18616109	99.8922481	99.994601	79.6579965	4.57016677	4.98304486	37.7787513	4.79586528	46.3036516
29	81.6182947	22.6734485	17.8144221	99.9813191	99.9813191	81.7796122	22.6385944	16.2417712	55.3410977	52.1458716	38.6349631
30	87.4030832	20.0375214	40.3934606	39.0787815	57.5509405	99.9739322	85.659763	82.2517273	64.0436512	69.9013339	28.7767502
31	3.54138696	3.56459953	99.9944233	99.9944233	99.9794901	3.82243641	4.0855158	99.9944233	51.8720873	52.0325029	51.441222
32	16.8643676	10.0494349	8.38957669	99.8016635	99.9838039	99.9559889	99.9838039	99.9838039	66.8765554	99.8788262	45.6975282
33	9.02105041	15.7645622	47.8592234	83.6002571	99.9898521	8.03520382	15.0376901	53.096511	41.5505438	31.8118928	35.6685819
34	99.9841201	99.9841201	1.52268209	99.9841201	99.9841201	99.9841201	24.3922416	99.9841201	78.2274555	99.9841201	40.7465463
