# Supplementary material for: Signal Quality Evaluation of Emerging EEG Devices
Source: Front Physiol. 2018 Feb 14;9:98. doi: 10.3389/fphys.2018.00098 (PMC5817086; doi:10.3389/fphys.2018.00098)
Supplement: Supplementary file 1 [file DataSheet1.ZIP › A-proportion_EPOC.pdf]

EPOC (all tasks)
Proportion of artifacts [%]

Vp	AF3	F7	F3	FC5	T7	P7	O1	O2	P8	T8	FC6	F4	F8	AF4	mean	median	std
11	9.87426821	10.0196538	7.3964334	12.5734641	8.48137751	6.67063009	7.20776731	6.53364642	6.94066395	9.91097321	7.54467495	7.18530888	9.88299338	10.7556899	8.64125322	8.01302623	1.8538357
12	0.5217834	1.16545564	0.60438125	0.7507853	0.5133823	0.65136379	1.2111952	0.74245379	0.59059074	0.70807374	0.58166308	0.57442422	2.4828974	0.59233281	0.83505591	0.62787252	0.52162536
13	88.2993663	68.5406525	67.6168587	69.072999	67.9693806	65.1546876	61.0510692	60.2846218	59.9743148	67.9238762	62.3997534	65.4682841	68.0949288	67.7747534	67.1161105	67.695806	6.91084992
14	0	2.42320102	99.9953026	0.23204985	0.2218192	0	0	0.28723404	0	0	0.70107937	0.1048951	0.96693318	0.84545988	7.55556959	0.22693452	26.6141337
15	0.4719407	2.8672494	0.94749101	6.72082368	1.16853943	0.18270767	0.2959897	0	3.86759856	3.91736508	0.24261182	0.17122604	0.33329822	0.22713658	1.52956985	0.40261946	2.03428846
16	80.0814588	80.2856182	80.0988804	80.1555006	80.1097689	80.145701	80.2496861	80.1212018	80.10868	80.1565895	80.2654745	80.0988804	80.2926957	80.2627523	80.1737777	80.1506008	0.07893303
17	99.9710928	99.9710928	99.9710928	99.9710928	99.9710928	99.9710928	99.9710928	99.9710928	99.9710928	99.9710928	99.9710928	99.9710928	99.9710928	99.9710928	99.9710928	99.9710928	2.9495E-14
18	3.69534355	1.95695509	1.07731328	1.3578657	0.933608	0.13354239	0.6704444	0.92540472	0.9208301	1.00597371	1.04802141	0.97726591	1.34687919	0.90822726	1.21126248	0.99161981	0.81792614
19	99.9230485	99.9230485	99.9230485	99.9230485	99.9230485	99.9230485	99.9230485	99.9230485	99.9230485	99.9230485	99.9230485	99.9230485	99.9230485	99.9230485	99.9230485	99.9230485	1.4747E-14
20	35.2137769	34.9991124	53.7367678	29.5802377	28.2396366	41.3456257	34.7052669	33.7098175	99.9058052	99.9317244	41.3789825	29.4614297	34.0486026	33.979011	45.0168426	34.8521897	24.1241885
21	0.97020629	5.80082601	0.51423343	1.84964579	0.5094697	0.62259606	0.77731353	0.81464768	2.04597455	1.49104215	2.00980272	1.27722429	4.3552647	1.03698454	1.71965939	1.15710442	1.54207298
22	22.8975152	23.6586476	10.7844293	24.4272619	9.82107185	46.024106	14.3573627	13.1882007	25.2211744	23.7689218	21.8776968	23.5907513	30.6774265	23.9123043	22.4433479	23.6246994	9.15268887
23	3.49674328	48.5445832	7.9108192	0.24934669	2.96338318	9.52963778	0.12957317	0	0	0.47103054	0	0	3.67099318	1.11400413	5.57715102	0.79251733	12.7416154
24	7.13691336	7.18961094	6.91919667	7.26341236	7.44582354	7.30569576	8.87402734	7.4541894	6.4689994	7.41754594	14.0476707	7.86904495	8.66859757	7.69170545	7.98231667	7.43168474	1.85587892
25	15.0177537	18.9976642	17.4851827	16.5286232	15.7518639	15.6697597	15.6392394	14.8827837	15.0114316	15.2266977	22.4269053	16.3957059	17.9665846	15.2507754	16.5893551	15.7108118	2.08636524
26	0.48248635	1.34076265	0.4740913	1.73096455	0.90542107	0.62162728	0.36980969	0.59755897	0.58802233	1.32324215	0.29778288	0.84496881	2.25580975	0.32597356	0.86846581	0.60959313	0.58746819
27	99.9287895	99.9287895	99.9287895	99.9287895	99.9287895	99.9287895	99.9287895	99.9287895	99.9287895	99.9287895	99.9287895	99.9287895	99.9287895	99.9287895	99.9287895	99.9287895	1.4747E-14
28	6.14077904	6.17844066	3.58763936	3.44722518	12.9494079	2.57073163	1.83979348	1.52791461	5.51772875	5.36606868	6.24003493	77.9737832	10.8325953	8.31138122	10.8916803	5.82925389	19.5809234
29	4.25639991	37.642444	0.26426698	9.4245092	0.64842102	0.73737734	0.68066656	0.53617043	0.62361076	0.59069923	7.55235301	0.42787074	7.8504379	5.79886638	5.50243525	0.70902195	9.82546435
30	27.7378144	12.6613922	8.5002895	9.255742	12.1201227	4.05950609	7.82723767	7.08953523	4.97559288	12.6571104	8.81841889	11.7304286	11.5450714	10.384829	10.6687922	9.82028549	5.61906784
31	4.67776215	5.52445113	1.1795829	4.24584357	1.75999954	1.04328688	0.71400631	0.41523667	0.48613699	1.98856932	2.81440172	1.39069209	2.75024399	3.46514153	2.31823963	1.87428443	1.64235459
32	1.09433943	1.86389444	0.22894022	3.91638062	1.04415625	0.24817264	0.91513171	0.303432	0.15557065	0.37625019	0.62960752	0.19299139	2.74682601	1.09704772	1.05805291	0.77236962	1.10310199
33	8.77446388	11.2757143	5.01341187	5.18877295	6.17647789	4.37101012	0.48006225	0.18229167	0.16601563	4.49843561	3.61975139	0.19314236	7.28485063	7.6630065	4.63481479	4.75592374	3.48802239
34	0.43818707	0.46419696	0	0.03135813	1.65912881	0.54262495	0	0.60340471	2.14847314	0.16258446	0	0.18581081	1.07530483	0.75740719	0.57632008	0.45119201	0.65495345
