# Supplementary material for: Signal Quality Evaluation of Emerging EEG Devices
Source: Front Physiol. 2018 Feb 14;9:98. doi: 10.3389/fphys.2018.00098 (PMC5817086; doi:10.3389/fphys.2018.00098)
Supplement: Supplementary file 1 [file DataSheet1.ZIP › A-proportion_gLADYbird.pdf]

g.LADYbird (all tasks)
Proportion of artifacts [%]

Vp	Fp1	Fp2	F3	Fz	F4	T7	C3	Cz	C4	T8	P3	Pz	P4	PO7	PO8	Oz	mean	median	std
11	7.54748191	7.35879289	2.90145242	2.83722393	2.84924055	3.10505153	3.63016527	2.76343228	2.79365224	5.62827173	2.73072837	2.37582954	2.3564237	2.35994003	2.43516594	2.58795699	3.51630058	2.81543808	1.72553031
12	0.43229436	0.45686756	0.47788412	0.43229436	0.47432747	0.46721417	0.47432747	0.47077082	0.48467408	0.48823073	0.49534402	0.45686756	0.47077082	0.49534402	0.46721417	0.50213399	0.47165998	0.47254915	0.0202081
13	1.72381891	1.87146238	0.51428854	0.50979558	0.55386783	0.71856712	0.75751424	0.550399	0.53368204	0.45981129	0.43564034	0.36164903	0.39303708	0.08830475	66.0639227	78.6154175	9.63444865	0.54204052	24.5890781
14	0	1.16880316	0.38905341	0	0	0	0	0.29872655	0	0.0635356	0	0	0	0.39879383	0	0	0.14493203	0	0.30915902
15	0.16045549	0.14751553	0.14973381	0.18559598	0.18559598	0.15269151	0.16156463	0.15084295	0.16748004	0.15638864	0.15454008	0.14086069	0.14566696	0.14566696	0.14270926	0.14160012	0.15555679	0.15176723	0.0139263
16	0	0	0	0	0	95.518463	0	0	0	0	0	0	0	0	0	0	5.96990394	0	23.8796157
17	0	0	0	0	0	0	0.21575553	0	0	0.14806314	0	0	0	0	0	0.16009852	0.03274482	0	0.0716236
18	0	0	0	0	0	0	0	0	0	0	0	0	0	0	0	0	0	0	0
19	0	0	0	0	0	0.02117464	0	0	0	0	0	0	0	0	0	0	0.00132341	0	0.00529366
20	0.18874874	0.1886516	0.07371076	0.17078993	0.66793652	0.08856502	0.42199438	0.23091751	0.36086047	0.2352115	0.07118834	0.22113688	2.61057239	0	99.7267288	99.9077115	12.8227953	0.22602719	33.9647188
21	0.09471813	0.107161	0.1234129	0.11528695	0.12772981	0.41401612	0.28821737	0.3539868	0.12747588	99.8652777	0.59294058	0.11097004	0.20542753	0.97484538	0.32469413	0.11300152	6.49619761	0.16657867	24.8995318
22	0.90063131	0.97600071	0.74542929	0.5469646	0.57102853	0.68571165	0.83391993	0.59267543	0.52476136	0.63102723	23.1352787	0.59449868	0.58524264	0.66871108	2.59661094	1.54161293	2.25813156	0.67721137	5.59196889
23	0	0.18600388	0	0	0	0.45483476	0	0	0	0	0	99.9316576	0	0.34721444	0	0	6.30748192	0	24.9668433
24	0.90535188	0.88859551	99.8030368	0.88631055	0.88859551	0.90255916	0.91525338	0.90281304	0.89341932	1.19004602	0.90332081	0.90535188	1.10153431	0.8855489	0.16756372	20.4242821	8.28522393	0.90306692	24.887756
25	0	1.94262033	0	0	0	0	0	0	0	0.12015582	0	0	0	0	0	0	0.12892351	0	0.48458029
26	0.84833911	1.3952578	0.11184431	0.10261716	0.10569288	0.12079186	0.25332737	0.14483838	0.13868695	0.17531596	0.12386758	0.15714126	0.10848898	0.11771614	0.12666368	0.1297394	0.26002055	0.12820154	0.35280928
27	1.43332754	1.442151	1.44244154	1.48006608	1.46212131	1.27867904	1.58337895	1.32145948	1.30769002	1.26520173	1.01000873	0.99387784	3.66525727	1.07077508	0.67753952	0.78999627	1.38899821	1.31457475	0.66055988
28	0.99349963	2.36612727	1.11045834	0.97771364	1.09581559	1.39120022	0.8331366	0.70035914	0.75611862	0.80384536	0.67385368	0.62539724	0.64703052	0.56682506	0.51149041	0.29813699	0.89693802	0.77998199	0.47448754
29	0.21705167	0.22402634	0.15372168	0.16488115	0.15595358	0.15372168	0.15595358	0.147026	0.25507966	0.28735632	0.12442808	0	0	0	0	0	0.12744998	0.15372168	0.09839752
30	0	0	0	0	0	0	0	0	0	0	0	0	0	0	0	0	0	0	0
31	0.10036352	0.12170065	0.12387387	0.13019599	0.12170065	0.13454244	0.26058954	0.13236921	0.13671566	0.24853992	0.14955745	0.14303777	0.1539039	0.13869132	0.12387387	0.11537854	0.14593964	0.13345582	0.04443415
32	0	0	62.1164582	0	0	0	0	0	0	0	0.1824939	0	0	0	0	0	3.89368451	0	15.5261397
33	0.1508717	0.31223796	0.95339687	0.81098304	1.13655962	2.06045937	1.62224316	1.07197754	79.9340698	0.90118273	35.0600761	34.3896668	0.98193505	1.31663527	0.27679264	57.1260697	13.6315723	1.10426858	24.638962
34	0	0	0	0	0	0	0	0	0	0	0	0	0	0	0	0	0	0	0
