# Supplementary material for: Signal Quality Evaluation of Emerging EEG Devices
Source: Front Physiol. 2018 Feb 14;9:98. doi: 10.3389/fphys.2018.00098 (PMC5817086; doi:10.3389/fphys.2018.00098)
Supplement: Supplementary file 1 [file DataSheet1.ZIP › A-proportion_gSAHARA.pdf]

g.SAHARA (all tasks)
Proportion of artifacts [%]

Vp	Fp1	Fp2	F3	Fz	F4	T7	C3	Cz	C4	T8	P3	Pz	P4	PO7	PO8	Oz	mean	median	std
11	18.0972718	15.9827716	56.5924567	39.960554	26.8637087	60.74941	60.1649019	59.4822867	45.4082151	44.6562548	55.4120191	47.9189843	41.672868	75.3950081	66.3874944	84.5381801	49.9551491	51.6655017	19.0126196
12	13.4215159	13.2720315	14.962589	20.603485	15.3968073	22.0479739	22.4665269	21.909348	27.4602384	17.2628019	19.2026917	33.1671421	17.9675485	18.2230794	23.2998814	33.2844754	20.8717585	19.9030884	6.15925757
13	37.3253066	38.8597112	53.5934538	56.3304322	51.5958721	79.6000621	70.7975082	40.7541014	58.2790138	68.2352665	51.2032301	46.5620511	54.842433	51.7817794	98.5537311	98.9490377	59.8289369	54.2179434	18.9440895
14	0.22414748	0.96414459	1.83125667	0.87811686	1.6095433	1.2195323	1.29671131	0.95110049	0.8058599	0.79394457	0.75392406	0.63939474	0.2429469	0.26969289	0.18386476	0.95205946	0.85101502	0.84198838	0.48371406
15	5.68130766	8.4962526	6.5362737	5.49470177	13.6517228	13.1906513	7.14487288	5.63898848	10.718954	12.0807835	11.9709014	16.5438741	9.87807417	35.9901483	18.028564	26.6548297	12.9813063	11.3449277	8.28886462
16	1.5874795	1.27576812	1.42642297	1.43764586	1.47279931	1.80855663	0.92190009	1.23861608	1.83225126	3.07615343	1.30324671	1.18716633	5.39641136	1.53955533	4.24563904	2.24310192	1.99954462	1.50617732	1.2268804
17	40.0428444	23.3899994	1.0746751	2.24252925	2.4230486	4.7524527	2.69946907	2.37305582	3.67137358	51.600183	18.84275	21.7423812	8.09191161	44.9864175	54.1539157	49.0110867	20.6936309	13.4673308	20.4520371
18	2.15397945	1.86126212	2.39544369	2.10032618	3.58400509	5.2206821	3.55326117	3.37317697	2.16565004	2.71483167	2.21473056	2.23199051	1.98365395	2.16177916	2.54404274	3.6016353	2.74127817	2.3137171	0.89902233
19	7.53556775	8.72154462	3.06407262	2.9514736	3.73244039	4.07966543	1.93377201	3.87552029	2.99359263	10.1846743	1.94444366	3.00124663	3.04447894	3.12626191	4.81654948	3.3967723	4.27512978	3.26151711	2.40988414
20	4.2004487	2.25164412	1.01326198	0.95877643	1.36308212	13.0163939	1.03959784	0.99482182	0.26739213	4.59053656	0.47035037	0.21902956	0.44565296	3.09644998	4.69771575	4.89977186	2.72030788	1.20133998	3.23408921
21	13.2825242	12.0522423	6.40107653	6.85692765	8.14713188	22.9134469	72.6382097	71.6757371	22.9624673	60.1808058	20.3480235	23.396467	40.6791298	30.5551713	45.4567005	43.2331139	31.2986985	23.1794671	22.1740453
22	2.21566951	3.37290359	1.71077959	1.09786628	1.25357996	1.62587088	2.75109458	2.99542768	1.28867402	1.44085225	2.85903283	1.2350733	2.75966151	0.98969201	0.98929362	2.06228681	1.9154849	1.66832523	0.80546538
23	2.8319664	10.5026103	1.81345263	0.36654149	0.50681336	0.37963203	0.23722167	0	0.43239458	0.25131241	0.67860507	0	4.09607492	4.48462549	0.73604423	0.90060319	1.76361861	0.59270922	2.72510104
24	12.4475076	14.2812754	10.8186217	9.73140754	14.3273244	13.4243563	9.53069053	10.4806359	2.9850818	6.38269981	11.087085	14.8761705	9.81359242	13.8510903	12.5490406	13.4567565	11.2527085	11.7672963	3.17722941
25	6.03869558	7.92659544	29.8761522	10.2819766	9.68437236	35.126194	41.4345677	40.6996875	32.7929488	27.1462958	47.5944226	3.96704119	58.566267	33.6156818	57.9246539	3.08154799	27.8598188	31.3345505	18.9699082
26	10.9499171	4.56339378	5.53278967	6.54522511	7.2773884	17.1352952	14.396296	13.6091986	5.92851511	15.2303152	5.70445813	3.74718281	17.3336455	13.8095304	9.33465366	11.5812476	10.1674408	10.1422854	4.65690288
27	1.42373991	1.18204114	3.30680676	4.04972189	3.16415784	6.8946185	2.83818309	3.81396027	1.95795799	9.45318317	5.61948242	10.7259708	1.05076658	5.43687014	5.11000736	5.1801449	4.4504758	3.93184108	2.79799929
28	11.5861363	17.0571439	15.6704475	12.9513937	12.2408573	25.7725131	17.4701983	15.6975359	14.49912	16.6790353	22.1579116	23.4923682	20.3827607	27.2984477	62.056113	41.0354141	22.2529623	17.2636711	12.9302387
29	6.16608972	8.33536899	3.0261991	2.70547293	3.95642406	6.14900237	4.65933437	2.4724366	4.26068028	3.1964991	2.6098027	4.10931008	2.83993933	3.81274967	3.92473868	9.00867332	4.45204508	3.94058137	1.98933263
30	21.7825862	12.9589591	28.0164522	19.6763305	20.2696832	27.2197639	23.096228	20.5273595	23.9290353	16.6171776	20.5731229	28.0238913	22.4830062	22.3058944	31.3838488	20.7327151	22.4747534	22.0442403	4.57819168
31	4.47627958	5.09336125	24.3724665	4.85791054	24.2250483	4.65796582	10.3314624	9.89870804	6.61996759	8.60103803	10.0971247	11.8484316	7.24880362	18.6159361	22.1786516	9.40510526	11.4080163	9.65190665	6.99604413
32	4.77590013	3.86999644	8.20701225	5.94310465	22.6714117	4.98968713	25.1702655	7.25703631	5.18082675	4.40613088	4.33515762	7.84908471	6.88874757	7.11122437	6.75839756	10.9270172	8.52131255	6.82357257	6.29026105
33	42.1849828	45.9791388	34.6693641	43.0395741	38.7664613	35.2092894	41.1742246	40.4025451	40.2518795	47.660873	81.9714809	67.1614972	83.2729381	81.7220204	68.0297956	11.5633024	50.1912105	42.6122784	20.3438998
34	9.48968803	5.79767398	2.50321279	2.10208359	2.12097674	2.99239738	3.33907673	3.07842257	2.02653908	3.49390473	2.58663222	6.89597791	5.22453224	4.56639893	3.85111067	7.07410389	4.19642072	3.41649073	2.16414926
