# Supplementary material for: Signal Quality Evaluation of Emerging EEG Devices
Source: Front Physiol. 2018 Feb 14;9:98. doi: 10.3389/fphys.2018.00098 (PMC5817086; doi:10.3389/fphys.2018.00098)
Supplement: Supplementary file 1 [file DataSheet1.ZIP › A-proportion_Jellyfish.pdf]

Jellyfish (all tasks)

Proportion of artifacts [%]

Vp	AF8	Fp2	Fp1	AF7	mean	median	std
11	15.9286865	34.1534207	34.3546153	34.2285456	29.666317	34.1909831	9.15879656
12	9.32884476	9.74882055	9.29804964	9.38536021	9.44026879	9.35710249	0.20885461
13	52.9494078	54.5120718	54.7183777	53.600339	53.9450491	54.0562054	0.82253234
14	0.20769684	1.99588456	0.30399807	0.1900384	0.67440447	0.25584745	0.88240902
15	1.50173716	22.82393	2.16608129	1.47128814	6.99075914	1.83390922	10.5603147
16	14.3139146	14.5049819	15.0383096	25.8232231	17.4201073	14.7716458	5.61045762
17	37.7798746	36.3511378	43.478204	37.2035574	38.7031934	37.491716	3.23699024
18	0	0	22.2563798	0	5.56409495	0	11.1281899
19	0.49326774	0.48695496	19.778382	1.64545028	5.60101375	1.06935901	9.46725795
20	2.73969466	0	2.72863191	3.40212668	2.21761331	2.73416329	1.5115764
21	38.6958886	15.9209646	35.2545062	27.3253835	29.2991857	31.2899448	10.1100044
22	55.7837543	6.74584131	2.50079647	21.858685	21.7222693	14.3022632	24.179618
23	41.0519221	41.0986813	43.0034513	41.1142991	41.5670884	41.1064902	0.95794192
24	53.6237912	54.2687117	53.778155	75.4323246	59.2757456	54.0234333	10.7745615
25	66.3890531	12.5852206	20.1097928	84.7211036	45.9512925	43.2494229	35.1277581
26	5.5387744	4.91712863	50.7083165	12.6441508	18.4520926	9.09146259	21.787957
27	1.44415569	1.351712	1.4158146	1.41665454	1.40708421	1.41623457	0.03919263
28	24.6619887	59.6002484	0	20.6378862	26.2250308	22.6499375	24.7340297
29	1.72724128	4.30944772	2.12461352	1.73718714	2.47462241	1.93090033	1.23713102
30	2.75474984	2.87626973	33.9095441	5.78605539	11.3316548	4.33116256	15.1170058
31	79.0333851	48.3623057	42.3392843	43.7972965	53.3830679	46.0798011	17.2916068
32	37.5022144	25.4697917	10.1938145	29.0573184	25.5557847	27.2635551	11.416004
33	41.6217028	37.9530413	0.03765473	24.1444373	25.939209	31.0487393	18.8359983
34	7.96552814	0	6.88028396	0.03186361	3.71941893	3.45607379	4.29932756
