# Supplementary material for: Signal Quality Evaluation of Emerging EEG Devices
Source: Front Physiol. 2018 Feb 14;9:98. doi: 10.3389/fphys.2018.00098 (PMC5817086; doi:10.3389/fphys.2018.00098)
Supplement: Supplementary file 1 [file DataSheet1.ZIP › A-proportion_Trilobite.pdf]

Trilobite (all tasks)
Proportion of artifacts [%]

Vp	Fp2	Fp1	AF4	AF3	F8	F4	Fz	F3	F7	FT8	FC4	FCz	FC3	FT7	T8	C4	Cz	C3	T7	TP8	CP4	CPz	CP3	TP7	P8	P4	Pz	P3	P7	O2	Oz	O1	mean	median	std
11	38.337885	39.0817637	35.5101341	32.0761528	67.9598996	18.659651	23.1096835	19.1850124	49.9517377	35.6951721	18.2300366	35.3553712	18.6917177	61.2011343	34.1974548	39.3589694	38.337885	39.0817637	35.5101341	32.0761528	67.9598996	18.659651	23.1096835	19.1850124	49.9517377	35.6951721	18.2300366	35.3553712	18.6917177	61.2011343	34.1974548	39.3589694	35.412611	35.4327527	14.4941429
12	83.8350349	83.4163382	80.5226763	80.3768751	80.4476316	80.5652895	87.7064322	80.4004569	79.3287073	80.8260232	80.4290128	80.4962903	80.3444475	80.4445546	80.5978422	80.9235325	83.8350349	83.4163382	80.5226763	80.3768751	80.4476316	80.5652895	87.7064322	80.4004569	79.3287073	80.8260232	80.4290128	80.4962903	80.3444475	80.4445546	80.5978422	80.9235325	81.2913216	80.5094833	2.02043108
13	68.1791811	69.0642049	66.3517345	67.3963098	6.13228309	6.06785493	13.244892	11.593324	6.99784514	67.7838312	5.29933003	5.33461991	7.39733428	5.38847979	75.0025904	5.63056191	68.1791811	69.0642049	66.3517345	67.3963098	6.13228309	6.06785493	13.244892	11.593324	6.99784514	67.7838312	5.29933003	5.33461991	7.39733428	5.38847979	75.0025904	5.63056191	30.4290236	9.49532915	30.4520094
14	62.8546959	63.2581834	7.18190736	8.23704193	5.7549288	5.83988324	6.27873848	5.95279303	9.22529733	13.1210065	5.98841439	5.88266136	6.88329	10.3447288	7.01009526	6.15079448	62.8546959	63.2581834	7.18190736	8.23704193	5.7549288	5.83988324	6.27873848	5.95279303	9.22529733	13.1210065	5.98841439	5.88266136	6.88329	10.3447288	7.01009526	6.15079448	14.3727788	6.94669263	18.7989244
15	54.7442676	54.716562	53.9051785	55.5659641	55.6465292	53.3830803	59.5253679	53.5877596	57.1204017	55.1118855	53.7124376	53.6002084	53.7455956	54.8585558	54.8067448	54.7589948	54.7442676	54.716562	53.9051785	55.5659641	55.6465292	53.3830803	59.5253679	53.5877596	57.1204017	55.1118855	53.7124376	53.6002084	53.7455956	54.8585558	54.8067448	54.7589948	54.9243458	54.7516312	1.54280303
16	71.8638697	71.7666815	27.9516604	27.4756009	99.71939	59.6471044	65.6043524	27.9168643	26.6885131	29.0832057	26.5171248	26.5500641	26.4512109	26.5025038	26.7406536	26.5392777	71.8638697	71.7666815	27.9516604	27.4756009	99.71939	59.6471044	65.6043524	27.9168643	26.6885131	29.0832057	26.5171248	26.5500641	26.4512109	26.5025038	26.7406536	26.5392777	41.6886298	27.6962326	23.3025559
17	39.96187	30.1167635	29.1218277	28.9773604	29.2375835	28.85725	42.3286268	28.8500289	29.3517639	34.9124951	29.0421498	29.3260178	29.1534656	31.5698915	29.2992472	29.4792279	39.96187	30.1167635	29.1218277	28.9773604	29.2375835	28.85725	42.3286268	28.8500289	29.3517639	34.9124951	29.0421498	29.3260178	29.1534656	31.5698915	29.2992472	29.4792279	31.2240981	29.3126325	4.11266667
18	84.7098555	84.4573596	84.7249047	84.3849662	85.5567274	99.8537721	133.472601	98.3929426	85.0247626	85.6178445	98.8279443	85.5380051	90.3604102	85.9883597	83.796729	84.7202706	84.7098555	84.4573596	84.7249047	84.3849662	85.5567274	99.8537721	133.472601	98.3929426	85.0247626	85.6178445	98.8279443	85.5380051	90.3604102	85.9883597	83.796729	84.7202706	90.9642159	85.5473662	12.4556293
19	30.9768177	31.0693763	30.0888883	28.8702903	73.5174645	28.0756578	31.0559278	27.9576147	28.1706154	29.3842704	27.9468201	31.6135716	28.7108836	28.0150243	28.0262173	27.9978429	30.9768177	31.0693763	30.0888883	28.8702903	73.5174645	28.0756578	31.0559278	27.9576147	28.1706154	29.3842704	27.9468201	31.6135716	28.7108836	28.0150243	28.0262173	27.9978429	31.9673302	28.7905869	10.9787658
20	38.1063692	37.9446428	36.9413217	36.923679	37.7731006	36.9012552	40.8929757	36.8823408	38.2249488	44.1926246	36.9763778	37.9819014	37.0450164	37.0345078	39.0323111	36.7959922	38.1063692	37.9446428	36.9413217	36.923679	37.7731006	36.9012552	40.8929757	36.8823408	38.2249488	44.1926246	36.9763778	37.9819014	37.0450164	37.0345078	39.0323111	36.7959922	38.1030853	37.4090585	1.91442091
21	25.2557253	38.5458812	20.139227	36.0896899	18.8069865	18.5616776	19.6506358	18.8916821	32.4259339	19.0433	19.9874687	19.782373	25.6338185	41.2089163	46.0360721	27.3632025	25.2557253	38.5458812	20.139227	36.0896899	18.8069865	18.5616776	19.6506358	18.8916821	32.4259339	19.0433	19.9874687	19.782373	25.6338185	41.2089163	46.0360721	27.3632025	26.7139119	22.6974762	9.09840468
22	52.4318702	52.3625319	48.4158447	46.3981925	46.9806802	46.1751542	46.8493563	46.0484659	52.0530841	52.6427368	46.3485922	54.77235	46.860456	46.4833523	47.1815097	46.5375882	52.4318702	52.3625319	48.4158447	46.3981925	46.9806802	46.1751542	46.8493563	46.0484659	52.0530841	52.6427368	46.3485922	54.77235	46.860456	46.4833523	47.1815097	46.5375882	48.6588603	46.9205681	2.97244969
23	46.21588	46.1955855	7.04334672	32.1586975	16.839766	17.2366633	22.7733191	22.0934408	12.630202	16.9370685	16.2523685	15.9184181	17.456181	9.56464049	16.2248103	16.281273	46.21588	46.1955855	7.04334672	32.1586975	16.839766	17.2366633	22.7733191	22.0934408	12.630202	16.9370685	16.2523685	15.9184181	17.456181	9.56464049	16.2248103	16.281273	20.7388538	16.8884172	11.2105956
24	53.5684342	53.7150466	5.04601212	5.75147618	6.07984503	6.36691273	10.5396039	9.80408039	5.26309809	8.37245043	4.94487378	5.83124185	6.28700082	4.71590497	10.8340024	6.88587085	53.5684342	53.7150466	5.04601212	5.75147618	6.07984503	6.36691273	10.5396039	9.80408039	5.26309809	8.37245043	4.94487378	5.83124185	6.28700082	4.71590497	10.8340024	6.88587085	12.7503659	6.32695678	15.8211606
25	24.0552842	48.8173203	17.1913215	17.6217408	14.1744288	13.856298	20.9941113	13.9869679	12.4447118	12.4494099	12.6300168	21.2587889	13.1482452	12.4600105	22.6558215	14.3419225	24.0552842	48.8173203	17.1913215	17.6217408	14.1744288	13.856298	20.9941113	13.9869679	12.4447118	12.4494099	12.6300168	21.2587889	13.1482452	12.4600105	22.6558215	14.3419225	18.2554	14.2581757	8.92049968
26	80.302723	88.7296332	84.870121	76.3192576	82.5929319	73.3195743	90.2709523	73.2967725	74.8242868	75.2523496	74.6244874	74.5739108	75.4726695	73.8653314	82.8556084	74.0453059	80.302723	88.7296332	84.870121	76.3192576	82.5929319	73.3195743	90.2709523	73.2967725	74.8242868	75.2523496	74.6244874	74.5739108	75.4726695	73.8653314	82.8556084	74.0453059	78.4509947	75.3625095	5.60161865
27	58.1689801	57.0196336	41.7053488	42.6183018	99.9912275	56.8907388	61.9834811	43.0453353	40.1020373	50.6360843	40.1244345	41.6705306	99.9912275	39.9135924	50.0396216	40.1278151	58.1689801	57.0196336	41.7053488	42.6183018	99.9912275	56.8907388	61.9834811	43.0453353	40.1020373	50.6360843	40.1244345	41.6705306	99.9912275	39.9135924	50.0396216	40.1278151	54.0017744	46.5424785	19.1517682
28	27.7884631	27.8047176	23.856141	23.4483501	31.3000944	23.3891032	30.2359885	23.4032136	23.4024876	31.7767006	23.3907246	31.5109217	23.8867269	44.1226257	39.3980132	23.6915994	27.7884631	27.8047176	23.856141	23.4483501	31.3000944	23.3891032	30.2359885	23.4032136	23.4024876	31.7767006	23.3907246	31.5109217	23.8867269	44.1226257	39.3980132	23.6915994	28.2753669	25.837595	6.16623215
29	50.0175115	50.8517387	50.9084193	48.6441232	99.8487257	50.6143296	51.7181912	51.0222701	44.3045896	47.5569957	44.5436781	44.9513773	97.8575041	44.3215827	45.0353915	44.3782325	50.0175115	50.8517387	50.9084193	48.6441232	99.8487257	50.6143296	51.7181912	51.0222701	44.3045896	47.5569957	44.5436781	44.9513773	97.8575041	44.3215827	45.0353915	44.3782325	54.1609163	49.3308173	17.3914154
30	36.8078693	47.1165057	50.9319761	47.023564	50.9280729	37.6546968	39.2405785	37.1367923	36.9922662	44.1091706	37.9906911	47.9527308	36.9185101	36.91771	47.9148577	43.3701086	36.8078693	47.1165057	50.9319761	47.023564	50.9280729	37.6546968	39.2405785	37.1367923	36.9922662	44.1091706	37.9906911	47.9527308	36.9185101	36.91771	47.9148577	43.3701086	42.4378813	41.3053435	5.41498672
31	41.5293216	41.3194974	29.7345134	28.4485478	97.9313941	41.9621005	29.9250935	29.3148033	28.6695396	29.1110122	31.2788589	38.2878913	97.253651	30.0409959	28.8351259	32.3775846	41.5293216	41.3194974	29.7345134	28.4485478	97.9313941	41.9621005	29.9250935	29.3148033	28.6695396	29.1110122	31.2788589	38.2878913	97.253651	30.0409959	28.8351259	32.3775846	41.0012457	30.6599274	22.2714727
32	23.3540749	23.331294	22.4796577	22.7822545	99.3068042	24.7761941	22.9668074	22.2573801	22.3636076	22.3209899	22.2842462	21.8687044	99.2849241	22.1356959	24.7160035	22.3192676	23.3540749	23.331294	22.4796577	22.7822545	99.3068042	24.7761941	22.9668074	22.2573801	22.3636076	22.3209899	22.2842462	21.8687044	99.2849241	22.1356959	24.7160035	22.3192676	32.4092441	22.6309561	25.6988139
33	15.7683229	27.7981809	14.8952882	24.6458488	99.997977	18.875102	15.8849644	15.1639741	15.155306	15.0655503	15.2784665	25.2533618	99.9965632	14.9647658	15.3889574	15.8258127	15.7683229	27.7981809	14.8952882	24.6458488	99.997977	18.875102	15.8849644	15.1639741	15.155306	15.0655503	15.2784665	25.2533618	99.9965632	14.9647658	15.3889574	15.8258127	28.1224026	15.7970678	27.9091245
34	53.6711418	52.8919421	50.3283415	49.8677178	49.8688672	49.8394825	52.0430062	49.9569314	50.2977108	51.6225642	49.8677013	49.869733	50.8266068	49.8701785	52.4708266	51.454941	53.6711418	52.8919421	50.3283415	49.8677178	49.8688672	49.8394825	52.0430062	49.9569314	50.2977108	51.6225642	49.8677013	49.869733	50.8266068	49.8701785	52.4708266	51.454941	50.9217308	50.3130261	1.25129598
