# Supplementary material for: Signal Quality Evaluation of Emerging EEG Devices
Source: Front Physiol. 2018 Feb 14;9:98. doi: 10.3389/fphys.2018.00098 (PMC5817086; doi:10.3389/fphys.2018.00098)
Supplement: Supplementary file 1 [file DataSheet1.ZIP › F-Band_BR8+_alpha.pdf]

BR8+ (tasks: 0-back, stop, rest measurements)
parietal alpha

Vp	Task	Pz	O1	O2	mean	median	std
11	0-back	7.533984	4.368188	5.45433	5.785501	5.45433	1.608671
12	0-back	0	2.020098	2.012321	1.344139	2.012321	1.164065
13	0-back	0.366556	2.56953	1.808111	1.581399	1.808111	1.118848
14	0-back	0	3.794569	2.931919	2.242163	2.931919	1.989098
15	0-back	0.63294	3.167427	1.968936	1.923101	1.968936	1.267865
16	0-back	0	0	0	0	0	0
17	0-back	6.22633	6.489813	5.225336	5.980493	6.22633	0.667123
18	0-back	0	4.009288	3.721108	2.576799	3.721108	2.23622
19	0-back	0	7.694441	7.299031	4.997824	7.299031	4.332756
20	0-back	9.05228	7.866715	6.784051	7.901015	7.866715	1.134503
21	0-back	4.048635	5.126642	5.587437	4.920905	5.126642	0.789762
22	0-back	10.35598	8.556667	6.966168	8.626273	8.556667	1.695979
23	0-back	7.919462	8.832739	9.381913	8.711371	8.832739	0.738741
24	0-back	0	0	0	0	0	0
25	0-back	0	0	0	0	0	0
26	0-back	2.313644	3.043202	3.540807	2.965885	3.043202	0.617224
27	0-back	1.944997	0	0	0.648332	0	1.122944
28	0-back	0	6.494338	5.940756	4.145031	5.940756	3.600358
29	0-back	16.02567	18.47928	9.178197	14.56105	16.02567	4.820412
30	0-back	20.02958	5.685773	5.25497	10.32344	5.685773	8.408525
31	0-back	10.64987	14.06105	8.670753	11.12722	10.64987	2.726669
32	0-back	11.25945	16.87335	12.74551	13.62611	12.74551	2.908702
33	0-back	15.37767	13.66931	4.255882	11.10095	13.66931	5.989232
34	0-back	0.012168	2.343295	0.083523	0.812995	0.083523	1.325758
11	stop	2.161916	4.213079	3.61843	3.331142	3.61843	1.055329
12	stop	8.949679	1.951455	1.663623	4.188252	1.951455	4.126027
13	stop	0	2.580193	2.35194	1.644045	2.35194	1.428351
14	stop	0	4.339996	0	1.446665	0	2.505698
15	stop	0	3.166293	2.196263	1.787519	2.196263	1.622238

16	stop	0	0	0	0	0	0
17	stop	11.63868	12.32023	3.079771	9.012896	11.63868	5.149525
18	stop	0.576256	4.266437	4.091068	2.977921	4.091068	2.08175
19	stop	11.02898	5.618286	5.418176	7.355146	5.618286	3.183203
20	stop	7.683901	6.698493	7.721481	7.367958	7.683901	0.580078
21	stop	3.946515	3.972473	5.296154	4.405047	3.972473	0.77183
22	stop	14.51493	14.78083	9.278776	12.85818	14.51493	3.102704
23	stop	0	0	0	0	0	0
24	stop	8.257983	8.959879	9.074533	8.764132	8.959879	0.442071
25	stop	0	0	0	0	0	0
26	stop	2.681558	3.001948	2.705736	2.796414	2.705736	0.178408
27	stop	0	1.604582	2.469652	1.358078	1.604582	1.253143
28	stop	2.450158	5.104098	4.20704	3.920432	4.20704	1.349984
29	stop	0	16.52146	8.790138	8.4372	8.790138	8.266383
30	stop	9.954562	8.980838	8.996175	9.310525	8.996175	0.557805
31	stop	19.98952	7.962556	0	9.31736	7.962556	10.06339
32	stop	0	0	0	0	0	0
33	stop	13.38261	17.86881	0.402791	10.55141	13.38261	9.070683
34	stop	0.321756	0	5.286524	1.869427	0.321756	2.963663
11	eyes opened	0.710338	4.215512	2.951474	2.625775	2.951474	1.77514
12	eyes opened	2.205459	2.74379	1.888063	2.279104	2.205459	0.432591
13	eyes opened	1.239718	0.723839	1.319875	1.094477	1.239718	0.323475
14	eyes opened	0	3.250486	2.4154	1.888629	2.4154	1.688055
15	eyes opened	0.693017	3.097574	2.306542	2.032378	2.306542	1.225499
16	eyes opened	0	0	0	0	0	0
17	eyes opened	0	1.192856	2.435655	1.209503	1.192856	1.217913
18	eyes opened	0	3.220171	4.54506	2.58841	3.220171	2.337463
19	eyes opened	0	4.931306	5.575199	3.502169	4.931306	3.050006
20	eyes opened	14.56406	9.219556	9.311878	11.03183	9.311878	3.059346
21	eyes opened	4.078873	4.468655	4.082552	4.210027	4.082552	0.223986
22	eyes opened	9.915216	2.783431	2.306561	5.001736	2.783431	4.261874
23	eyes opened	0.968676	0.693079	1.013237	0.891664	0.968676	0.173417
24	eyes opened	0	1.846663	1.886947	1.244537	1.846663	1.077989

25	eyes opened	0	2.631746	2.790147	1.807298	2.631746	1.567168
26	eyes opened	8.579395	3.717617	4.95452	5.750511	4.95452	2.526741
27	eyes opened	0	1.567661	1.79565	1.121104	1.567661	0.977574
28	eyes opened	2.094442	3.68887	1.836848	2.540053	2.094442	1.003207
29	eyes opened	4.889597	6.622716	5.645745	5.719352	5.645745	0.868901
30	eyes opened	6.392334	7.980219	2.347206	5.573253	6.392334	2.904458
31	eyes opened	9.302623	8.563915	1.643948	6.503495	8.563915	4.224668
32	eyes opened	2.436192	1.202285	3.333215	2.323897	2.436192	1.069894
33	eyes opened	18.68859	13.5114	4.044884	12.08162	13.5114	7.425813
34	eyes opened	11.54408	1.936146	8.928187	7.46947	8.928187	4.96729
11	eyes closed	0.44819	2.589007	1.965662	1.66762	1.965662	1.101089
12	eyes closed	2.139495	3.123921	2.387694	2.55037	2.387694	0.511978
13	eyes closed	1.770364	0.715223	1.492079	1.325889	1.492079	0.54685
14	eyes closed	1.420251	2.190396	2.548971	2.053206	2.190396	0.576731
15	eyes closed	3.495946	4.868221	4.808992	4.391053	4.808992	0.775751
16	eyes closed	0	0	0	0	0	0
17	eyes closed	5.504809	4.251536	4.174405	4.643583	4.251536	0.746839
18	eyes closed	0	3.174843	2.181381	1.785408	2.181381	1.624039
19	eyes closed	0	4.629465	4.875305	3.168256	4.629465	2.746543
20	eyes closed	6.461252	5.591016	5.49206	5.84811	5.591016	0.533297
21	eyes closed	6.353014	6.336035	8.536756	7.075268	6.353014	1.265714
22	eyes closed	3.961919	4.930517	3.625865	4.172767	3.961919	0.677401
23	eyes closed	0	0	0	0	0	0
24	eyes closed	0	0	0	0	0	0
25	eyes closed	0	1.445816	1.82176	1.089192	1.445816	0.961815
26	eyes closed	3.974537	3.809717	3.785057	3.856437	3.809717	0.103018
27	eyes closed	0	0.999817	1.105372	0.701729	0.999817	0.610003
28	eyes closed	2.531732	2.931465	1.896751	2.453316	2.531732	0.521795
29	eyes closed	0	3.843437	5.53805	3.127162	3.843437	2.837655
30	eyes closed	0	0	0	0	0	0
31	eyes closed	11.5593	11.63917	0	7.732822	11.5593	6.69694
32	eyes closed	1.013371	1.902009	2.00979	1.641723	1.902009	0.546831
33	eyes closed	4.475255	4.121362	2.933262	3.843293	4.121362	0.80773

34	eyes closed	0	2.810962	0	0.936987	0	1.622909
