# Supplementary material for: Signal Quality Evaluation of Emerging EEG Devices
Source: Front Physiol. 2018 Feb 14;9:98. doi: 10.3389/fphys.2018.00098 (PMC5817086; doi:10.3389/fphys.2018.00098)
Supplement: Supplementary file 1 [file DataSheet1.ZIP › F-Band_BR8+_theta.pdf]

BR8+ (tasks: 0-back, stop)
frontal theta

Vp	Task	Fp1	Fp2	Fz	mean	median	std
11	0-back	8.661286	6.15209	6.144833	6.98607	6.15209	1.450785
12	0-back	11.15922	11.00413	0	7.387784	11.00413	6.398478
13	0-back	0.84871	0.006428	7.56282	2.805986	0.84871	4.14101
14	0-back	7.09405	7.285775	6.424192	6.934672	7.09405	0.452363
15	0-back	1.565311	24.51579	11.77116	12.61742	11.77116	11.49862
16	0-back	4.108807	11.5605	4.975465	6.881592	4.975465	4.075161
17	0-back	6.281383	6.45643	5.222031	5.986615	6.281383	0.667908
18	0-back	7.571061	11.06575	10.95603	9.864278	10.95603	1.986742
19	0-back	1.768671	6.285734	7.453459	5.169288	6.285734	3.002339
20	0-back	6.000016	16.37353	11.64218	11.33857	11.64218	5.193415
21	0-back	0	0	6.208661	2.069554	0	3.584572
22	0-back	10.43521	11.33435	7.22021	9.663257	10.43521	2.162978
23	0-back	6.289776	5.683516	2.911953	4.961748	5.683516	1.80087
24	0-back	17.99704	3.182381	5.834904	9.004775	5.834904	7.899658
25	0-back	6.372045	5.778161	4.741572	5.630593	5.778161	0.825193
26	0-back	0	5.649269	3.76347	3.13758	3.76347	2.876172
27	0-back	10.11379	9.77454	8.50263	9.463654	9.77454	0.849381
28	0-back	10.76428	8.292395	8.167906	9.074861	8.292395	1.464405
29	0-back	15.79977	17.81514	9.125572	14.24683	15.79977	4.548171
30	0-back	22.71754	5.54397	5.502812	11.25477	5.54397	9.927068
31	0-back	19.22481	22.02338	8.670753	16.63965	19.22481	7.041694
32	0-back	7.582947	7.231241	6.782363	7.198851	7.231241	0.401273
33	0-back	8.836687	9.846512	4.18463	7.62261	8.836687	3.019887
34	0-back	2.233743	0.547749	5.670915	2.817469	2.233743	2.610988
11	stop	7.098184	6.584348	4.838451	6.173661	6.584348	1.184524
12	stop	26.63998	23.74798	8.574155	19.65404	23.74798	9.703802
13	stop	0	0.941956	6.261826	2.401261	0.941956	3.376358
14	stop	27.04712	21.08226	12.58494	20.23811	21.08226	7.267951
15	stop	0	36.31665	20.03773	18.78479	20.03773	18.19072

16	stop	6.537101	9.580838	7.336664	7.818201	7.336664	1.577971
17	stop	5.14868	6.948124	3.125524	5.074109	5.14868	1.912391
18	stop	8.696586	36.01934	17.33327	20.68307	17.33327	13.966
19	stop	2.933752	6.288687	5.43752	4.886653	5.43752	1.743986
20	stop	22.95014	16.06399	10.67545	16.56319	16.06399	6.15255
21	stop	0	0.313751	7.5568	2.623517	0.313751	4.275227
22	stop	14.82254	14.3825	7.244868	12.14997	14.3825	4.253638
23	stop	11.76399	11.50242	8.062431	10.44295	11.50242	2.065731
24	stop	26.35101	7.650401	9.401173	14.46753	9.401173	10.32856
25	stop	10.23258	19.49934	8.837587	12.8565	10.23258	5.794995
26	stop	0	10.61787	6.683014	5.766962	6.683014	5.367882
27	stop	8.579897	17.62093	7.574686	11.2585	8.579897	5.532897
28	stop	23.80553	18.67832	11.22912	17.90432	18.67832	6.323833
29	stop	0	16.34756	9.373195	8.573586	9.373195	8.203063
30	stop	2.355161	1.411279	3.882243	2.549561	2.355161	1.2469
31	stop	14.44615	24.97351	0	13.13989	14.44615	12.53789
32	stop	4.428242	8.452015	6.291191	6.390483	6.291191	2.013723
33	stop	26.04329	15.67861	0.402791	14.04156	15.67861	12.8984
34	stop	0.061005	0.10298	5.475959	1.879981	0.10298	3.114279
