# Supplementary material for: Signal Quality Evaluation of Emerging EEG Devices
Source: Front Physiol. 2018 Feb 14;9:98. doi: 10.3389/fphys.2018.00098 (PMC5817086; doi:10.3389/fphys.2018.00098)
Supplement: Supplementary file 1 [file DataSheet1.ZIP › F-Band_EPOC_alpha.pdf]

EPOC (tasks: 0-back, stop, rest measurements)
parietal alpha

Vp	Task	P7	O1	O2	P8	mean	median	std
11	0-back	0.54799	0.600014	1.172411	1.421836	0.935563	0.886213	0.430257
12	0-back	11.54377	6.976012	15.91375	7.147813	10.39534	9.345791	4.243031
13	0-back	6.143188	6.096014	6.101984	6.126778	6.116991	6.114381	0.021964
14	0-back	7.053414	7.250727	7.402495	7.620887	7.331881	7.326611	0.239889
15	0-back	7.074202	29.96232	8.021922	3.643929	12.17559	7.548062	12.00603
16	0-back	0.091033	0.237178	0.143594	0.056134	0.131984	0.117313	0.078805
17	0-back	12.75585	12.75293	12.75554	12.75594	12.75507	12.7557	0.001435
18	0-back	7.231271	8.52391	10.50977	8.457833	8.680695	8.490871	1.356537
19	0-back	0.03813	0.046822	0.047146	0.050199	0.045574	0.046984	0.005191
20	0-back	6.781847	0.014987	0.914389	13.7915	5.37568	3.848118	6.36248
21	0-back	10.65053	19.38369	24.15439	12.19035	16.59474	15.78702	6.315556
22	0-back	6.958584	8.797583	17.77568	5.442814	9.743666	7.878084	5.527579
23	0-back	7.038682	10.8686	6.966337	6.964099	7.95943	7.002509	1.939759
24	0-back	4.087273	5.98444	7.553023	8.732232	6.589242	6.768731	2.012206
25	0-back	0.259053	0.024969	0.061922	0.43131	0.194313	0.160487	0.188471
26	0-back	14.19309	18.43024	29.34291	29.48096	22.8618	23.88657	7.758936
27	0-back	6.640902	6.545705	6.764776	6.597881	6.637316	6.619391	0.093464
28	0-back	29.40685	31.6429	40.15453	4.262645	26.36673	30.52488	15.4464
29	0-back	42.76994	62.95621	68.57094	63.70244	59.49988	63.32933	11.42779
30	0-back	14.1694	18.74003	32.12931	29.8372	23.71899	24.28862	8.643895
31	0-back	9.384393	13.38217	14.53595	13.4238	12.68158	13.40298	2.26214
32	0-back	7.065883	26.35551	7.390466	7.40787	12.05493	7.399168	9.535015
33	0-back	2.181981	6.984497	6.976791	6.969126	5.778099	6.972958	2.39742
34	0-back	6.968613	7.015483	7.143466	6.971636	7.0248	6.99356	0.081959
11	stop	0.213242	0.115782	0.244193	0.671375	0.311148	0.228717	0.246306
12	stop	7.064067	7.046727	7.084954	7.176647	7.093099	7.074511	0.05785
13	stop	6.176761	6.239226	6.256489	6.206616	6.219773	6.222921	0.035354
14	stop	7.069293	7.231175	40.57243	7.540583	15.60337	7.385879	16.64719
15	stop	7.011797	7.028945	7.216591	7.082599	7.084983	7.055772	0.092777

16	stop	23.27143	23.64376	23.71876	23.51793	23.53797	23.58084	0.196062
17	stop	26.64984	26.6579	26.64127	26.65352	26.65063	26.65168	0.007058
18	stop	7.082796	7.898897	65.03434	39.27547	29.82288	23.58718	27.85062
19	stop	25.56883	25.55778	25.56008	25.55706	25.56094	25.55893	0.005416
20	stop	3.297779	18.23123	19.87691	3.589718	11.24891	10.91047	9.038416
21	stop	9.765755	28.49587	24.7969	10.2716	18.33253	17.53425	9.720251
22	stop	9.97263	7.144883	7.30193	7.220558	7.91	7.261244	1.376581
23	stop	10.13872	6.973548	6.968576	6.974938	7.763946	6.974243	1.583188
24	stop	4.78823	5.922961	8.026522	7.82777	6.641371	6.875365	1.557394
25	stop	7.027458	5.692013	5.000525	7.140804	6.2152	6.359735	1.043339
26	stop	8.506127	6.962298	22.6401	26.30476	16.10332	15.57311	9.799226
27	stop	14.21851	14.17116	14.11394	14.16125	14.16621	14.1662	0.042882
28	stop	23.16759	7.210329	37.52165	7.547063	18.86166	15.35732	14.49724
29	stop	7.305144	8.462676	10.51788	10.48896	9.193664	9.475818	1.584528
30	stop	14.61638	10.90204	21.36109	21.05471	16.98355	17.83554	5.109646
31	stop	6.973009	7.009598	6.995603	7.016804	6.998753	7.0026	0.019289
32	stop	18.73329	23.65575	28.24474	26.99146	24.40631	25.32361	4.249044
33	stop	6.785411	10.16698	6.976309	6.980592	7.727324	6.978451	1.628984
34	stop	9.939048	7.009278	7.102439	8.692579	8.185836	7.897509	1.401021
11	eyes opened	7.058138	11.37368	7.194468	6.131629	7.939478	7.126303	2.337652
12	eyes opened	7.045125	7.006297	7.000603	7.005445	7.014367	7.005871	0.020658
13	eyes opened	0.652076	0.664729	0.10481	0.18246	0.401018	0.417268	0.298932
14	eyes opened	6.927528	6.955278	7.052939	7.108845	7.011147	7.004109	0.084469
15	eyes opened	7.073837	7.117208	7.504818	7.321838	7.254425	7.219523	0.198898
16	eyes opened	3.144828	3.141435	3.076755	3.128851	3.122967	3.135143	0.031566
17	eyes opened	12.75752	12.75754	12.75669	12.75747	12.75731	12.75749	0.000409
18	eyes opened	7.083046	7.40149	7.799433	7.376796	7.415191	7.389143	0.294179
19	eyes opened	6.930062	4.152697	3.940396	4.356625	4.844945	4.254661	1.400427
20	eyes opened	6.972361	6.981413	7.135202	7.74919	7.209542	7.058308	0.367443
21	eyes opened	6.927523	6.968317	6.974798	6.921706	6.948086	6.94792	0.027335
22	eyes opened	6.968359	7.02984	7.075531	7.045793	7.029881	7.037816	0.045174
23	eyes opened	6.967689	6.973444	6.967006	6.965018	6.968289	6.967347	0.003619
24	eyes opened	6.965312	6.970195	6.942997	6.956482	6.958746	6.960897	0.011935

25	eyes opened	6.944394	0.481058	6.957607	6.993464	5.344131	6.951001	3.242115
26	eyes opened	7.023804	6.966052	7.229422	7.269007	7.122071	7.126613	0.149569
27	eyes opened	2.576716	3.139247	2.955373	2.684415	2.838938	2.819894	0.255852
28	eyes opened	6.98971	7.004841	7.058283	7.047912	7.025187	7.026377	0.033087
29	eyes opened	6.998556	7.204839	7.506453	7.536605	7.311613	7.355646	0.256898
30	eyes opened	0.126816	0.018226	0.033621	29.83861	7.504319	0.080219	14.88961
31	eyes opened	0.607443	0.315297	0.372637	0.592836	0.472053	0.482736	0.149861
32	eyes opened	6.998696	7.005222	7.076849	7.11307	7.048459	7.041035	0.055756
33	eyes opened	6.99642	6.956211	6.968835	6.969261	6.972682	6.969048	0.016944
34	eyes opened	6.961178	6.950507	7.032276	6.969354	6.978329	6.965266	0.036783
11	eyes closeed	7.147138	7.243051	7.337162	7.328326	7.263919	7.285688	0.088668
12	eyes closeed	6.993449	6.97219	7.015549	7.05455	7.008935	7.004499	0.035188
13	eyes closeed	0.634592	6.932461	3.326192	2.274024	3.291817	2.800108	2.667847
14	eyes closeed	7.009566	7.072274	7.123683	7.17588	7.095351	7.097979	0.071132
15	eyes closeed	6.933935	7.151073	7.269208	7.052191	7.101602	7.101632	0.142704
16	eyes closeed	3.151488	3.173909	3.187637	3.136923	3.162489	3.162698	0.022638
17	eyes closeed	6.838281	7.007828	6.288957	6.908822	6.760972	6.873552	0.322269
18	eyes closeed	7.182864	7.627881	8.222436	7.649085	7.670567	7.638483	0.426105
19	eyes closeed	15.08701	15.00284	14.99642	15.00977	15.02401	15.00631	0.042354
20	eyes closeed	6.978004	6.978641	7.230408	9.97263	7.789921	7.104525	1.459984
21	eyes closeed	7.057668	7.16584	7.200434	7.02224	7.111545	7.111754	0.085103
22	eyes closeed	6.991342	7.059434	44.26172	40.88138	24.79847	23.97041	20.56895
23	eyes closeed	6.959829	6.972937	6.977579	6.977583	6.971982	6.975258	0.008393
24	eyes closeed	7.023108	7.019302	7.062417	7.025015	7.03246	7.024062	0.020112
25	eyes closeed	6.996095	2.461101	3.070111	7.107948	4.908813	5.033103	2.48764
26	eyes closeed	7.011121	12.75754	7.289838	7.405384	8.615972	7.347611	2.766003
27	eyes closeed	2.962468	3.380028	3.338665	3.020946	3.175527	3.179805	0.214262
28	eyes closeed	7.219506	7.144851	7.296024	7.186028	7.211602	7.202767	0.06403
29	eyes closeed	7.252616	7.675092	8.753274	9.292145	8.243282	8.214183	0.94242
30	eyes closeed	0.57183	12.75754	7.348012	23.02881	10.92655	10.05278	9.48408
31	eyes closeed	0.077917	0.108522	0.128125	0.041376	0.088985	0.093219	0.037872
32	eyes closeed	7.071873	7.260251	7.271775	7.307631	7.227882	7.266013	0.105945
33	eyes closeed	6.988852	6.973841	6.969329	6.968914	6.975234	6.971585	0.009349

34	eyes closeed	6.994516	7.161861	7.359484	6.964104	7.119991	7.078188	0.181801
