# Supplementary material for: Signal Quality Evaluation of Emerging EEG Devices
Source: Front Physiol. 2018 Feb 14;9:98. doi: 10.3389/fphys.2018.00098 (PMC5817086; doi:10.3389/fphys.2018.00098)
Supplement: Supplementary file 1 [file DataSheet1.ZIP › F-Band_EPOC_theta.pdf]

EPOC (tasks: 0-back, stop)
frontal theta

Vp	Task	AF3	F7	F3	F4	F8	AF4	mean	median	std
11	0-back	0.816242	0.251902	0.426415	0.577619	0.410873	0.364858	0.474651	0.418644	0.197663
12	0-back	18.00623	18.81789	20.39962	20.35345	15.10845	20.11433	18.8	19.46611	2.047311
13	0-back	47.05219	47.13997	47.05471	47.16796	47.1852	46.95082	47.09181	47.09734	0.089065
14	0-back	20.26465	23.0977	20.45203	23.52309	20.33198	17.97236	20.9403	20.392	2.059068
15	0-back	20.23653	19.39565	20.38887	20.3319	20.24827	20.0845	20.11429	20.2424	0.36687
16	0-back	0.250032	14.9458	0.058097	0.135617	14.84767	17.27353	7.918458	7.548849	8.556606
17	0-back	13.24051	13.24012	13.23858	13.23976	13.23768	13.24098	13.23961	13.23994	0.001244
18	0-back	13.20207	19.96745	20.48033	20.60532	20.14034	19.84562	19.04019	20.0539	2.87493
19	0-back	5.784466	5.47656	4.560986	5.762642	5.638501	5.411644	5.439133	5.55753	0.455403
20	0-back	1.013888	0.351944	4.060818	1.287166	1.842833	3.291666	1.974719	1.564999	1.423578
21	0-back	20.22529	9.097874	20.22848	20.25655	2.966931	20.18069	15.49264	20.20299	7.580042
22	0-back	19.40072	1.655493	17.5668	37.29463	0.549291	1.457417	12.98739	9.611145	14.62192
23	0-back	7.492115	18.35746	13.06186	20.45286	17.27827	11.82022	14.7438	15.17006	4.817439
24	0-back	16.43262	15.98233	14.56166	15.82756	16.33855	15.96376	15.85108	15.97304	0.673631
25	0-back	1.162035	0.914994	0.495969	0.563115	0.457252	0.736648	0.721669	0.649881	0.274779
26	0-back	20.70946	28.23402	29.40225	30.14126	27.69507	21.23918	26.23687	27.96455	4.169127
27	0-back	52.61388	52.42456	52.59457	53.06755	52.3457	53.22481	52.71184	52.60423	0.354853
28	0-back	21.44119	25.32426	19.55403	0.074161	3.458322	6.432697	12.71411	12.99336	10.64759
29	0-back	25.79729	24.75259	17.74802	18.76848	21.57069	24.33667	22.16229	22.95368	3.346774
30	0-back	48.23524	1.791955	17.20954	17.54005	6.239585	16.19532	17.86862	16.70243	16.24295
31	0-back	20.61823	20.12342	19.71238	21.20222	21.75342	19.39197	20.46694	20.37082	0.901275
32	0-back	21.36961	28.78558	20.4248	21.40966	19.41872	20.76649	22.02914	21.06805	3.389712
33	0-back	14.17373	15.77083	11.53426	20.42913	14.81562	15.56172	15.38088	15.18867	2.906643
34	0-back	19.17565	18.68816	20.42286	19.88598	19.92354	18.9956	19.5153	19.53081	0.662923
11	stop	0.744215	0.44216	0.422687	0.568358	0.406465	0.724975	0.551477	0.505259	0.153036
12	stop	20.09347	20.39261	20.23116	20.27324	17.55076	20.10309	19.77406	20.16713	1.094902
13	stop	47.50257	47.51502	47.51119	47.51789	47.53746	47.51297	47.51618	47.514	0.01164
14	stop	20.32765	20.34379	20.4562	20.40736	16.84622	20.27563	19.77614	20.33572	1.436748
15	stop	22.54691	22.05167	22.70824	20.89782	21.11769	22.46046	21.9638	22.25607	0.774704

16	stop	38.13107	37.5634	37.50271	37.69856	37.52671	37.58483	37.66788	37.57412	0.236858
17	stop	26.16535	26.12967	26.2523	26.2101	25.98494	26.25967	26.16701	26.18772	0.102237
18	stop	4.595172	12.36659	17.30882	18.97908	14.86081	13.39111	13.5836	14.12596	5.038447
19	stop	35.8355	35.79705	35.82332	35.82824	35.81644	35.83093	35.82191	35.82578	0.013827
20	stop	9.870545	3.425832	1.335657	23.06973	20.05672	19.86704	12.93759	14.86879	9.33643
21	stop	14.9416	15.36042	14.45832	14.61849	12.91475	13.85522	14.35814	14.53841	0.867076
22	stop	20.15408	20.33933	20.37649	20.40327	20.37884	20.38274	20.33912	20.37767	0.092986
23	stop	0.462678	0.970852	20.43605	20.43694	11.97028	10.22612	10.75049	11.0982	8.841726
24	stop	14.8349	15.02687	13.97323	13.39489	14.66946	13.80476	14.28402	14.32135	0.651261
25	stop	50.63864	11.05961	67.30606	61.17088	20.08679	20.76934	38.50522	35.70399	24.07281
26	stop	24.60919	23.42734	20.09604	22.80551	24.3697	27.72641	23.83903	23.89852	2.499364
27	stop	46.83874	46.85515	46.82097	46.8533	46.84763	46.8846	46.85007	46.85047	0.021021
28	stop	4.441057	14.88932	12.88077	0.671376	11.34629	4.469141	8.116325	7.907715	5.678783
29	stop	30.98666	8.60649	20.4952	20.59101	23.78437	29.21626	22.28	22.18769	7.989791
30	stop	13.92086	2.69214	15.0622	2.333894	4.158854	5.346315	7.252377	4.752585	5.721052
31	stop	25.46541	25.08252	20.51381	20.43423	20.13779	22.5839	22.36961	21.54886	2.414975
32	stop	22.67461	16.67345	7.413828	22.21552	20.58544	23.16767	18.78842	21.40048	6.051596
33	stop	19.60756	17.44306	18.38443	20.49608	18.33378	18.67874	18.82394	18.53158	1.074381
34	stop	5.437559	4.056694	20.4538	2.56674	5.342055	7.320978	7.529638	5.389807	6.52582
