# Supplementary material for: Signal Quality Evaluation of Emerging EEG Devices
Source: Front Physiol. 2018 Feb 14;9:98. doi: 10.3389/fphys.2018.00098 (PMC5817086; doi:10.3389/fphys.2018.00098)
Supplement: Supplementary file 1 [file DataSheet1.ZIP › F-Band_gLADYbird_alpha.pdf]

g.LADYbird (tasks: 0-back, stop, rest measurements)
parietal alpha

Vp	Task	P3	Pz	P4	PO7	PO8	Oz	mean	median	std
11	0-back	26.9451	26.68218	23.28173	27.29979	17.7986	18.23402	23.37357	24.98195	4.395943
12	0-back	24.03861	24.12477	20.61838	24.23205	13.8141	23.79228	21.77003	23.91544	4.134376
13	0-back	12.81757	13.22736	14.64368	11.92051	1.834783	0.009576	9.075578	12.36904	6.402478
14	0-back	47.55994	48.05445	44.52651	42.36274	42.91661	39.16667	44.09782	43.72156	3.362946
15	0-back	40.65202	45.78243	36.31546	31.26523	20.82559	25.94579	33.46442	33.79034	9.301933
16	0-back	16.80407	16.4147	17.09786	16.43812	13.4151	15.90149	16.01189	16.42641	1.33464
17	0-back	23.01851	23.90916	28.59531	18.94296	18.94629	16.05725	21.57825	20.9824	4.496665
18	0-back	63.33964	70.53973	71.0022	46.24441	61.06928	46.09767	59.71549	62.20446	11.19318
19	0-back	24.67012	24.1028	20.67981	17.44252	16.26941	16.2331	19.89963	19.06116	3.839068
20	0-back	39.75246	41.59019	16.69876	36.21728	28.2401	28.23981	31.78977	32.22869	9.295123
21	0-back	62.14893	65.63774	66.96304	29.48439	30.24736	21.6179	46.01656	46.19814	20.98225
22	0-back	17.24774	38.68013	32.6966	22.59499	17.08855	17.73541	24.34057	20.1652	9.219777
23	0-back	13.87047	29.68925	13.72621	15.1754	14.3171	13.38756	16.69433	14.09379	6.396015
24	0-back	16.80539	18.17584	16.43871	17.8633	15.58956	12.97834	16.30852	16.62205	1.885474
25	0-back	38.73099	38.20251	41.55489	43.01389	34.43394	29.9258	37.64367	38.46675	4.806233
26	0-back	29.98773	28.81418	29.23191	28.52316	27.19258	25.28128	28.17181	28.66867	1.689466
27	0-back	22.07622	22.57826	20.93913	18.60322	17.3011	15.71867	19.5361	19.77117	2.76093
28	0-back	39.84353	33.61769	34.19138	37.81529	37.75867	29.37731	35.43398	35.97502	3.795923
29	0-back	55.23442	58.08878	58.58144	67.00434	74.81422	64.62456	63.05796	61.603	7.241234
30	0-back	42.51489	44.31109	44.65575	28.52642	22.25041	22.59226	34.1418	35.52066	10.86642
31	0-back	24.69527	25.1509	23.72193	14.86685	16.08154	15.09664	19.93552	19.90174	5.06264
32	0-back	30.95086	30.78145	31.97306	29.46542	29.13565	25.97277	29.7132	30.12343	2.105505
33	0-back	13.45199	13.85396	19.56263	20.03113	15.36751	14.39777	16.11083	14.88264	2.930315
34	0-back	21.74586	21.47882	20.74065	11.89564	11.39347	11.93998	16.5324	16.34032	5.260325
11	stop	36.24186	33.86555	32.47311	29.19552	23.45673	18.36755	28.93339	30.83431	6.8113
12	stop	11.9606	12.00211	10.26473	10.72663	6.848763	9.541574	10.22407	10.49568	1.912574
13	stop	17.68767	17.91304	16.82865	12.84103	13.09859	13.08907	15.24301	14.96362	2.474964
14	stop	31.66587	33.00367	30.585	28.16226	28.39593	25.90058	29.61889	29.49047	2.60858
15	stop	32.06403	40.15038	31.03143	20.14767	15.75523	20.52938	26.61302	25.7804	9.264978

16 stop	15.57117	16.13431	16.95472	15.4913	13.65297	13.20699	15.16858	15.53124	1.451484
17 stop	16.63978	16.91297	19.01419	16.34065	15.56357	14.67306	16.52404	16.49022	1.466135
18 stop	56.63885	62.86874	63.05192	43.93748	44.23864	39.64904	51.73078	50.43875	10.3841
19 stop	21.94072	21.88765	20.85595	14.03464	14.56069	15.33045	18.10168	18.0932	3.83189
20 stop	50.34292	51.22493	15.5776	44.42322	0.007099	11.61754	28.86555	30.00041	22.40595
21 stop	47.39821	47.33836	45.27607	15.75628	17.3203	13.03088	31.02002	31.29818	17.21651
22 stop	16.60028	36.85123	35.68198	26.52416	24.08863	22.56778	27.05234	25.3064	7.860134
23 stop	14.96758	16.19662	16.09665	16.74224	17.9383	15.15919	16.18343	16.14664	1.088751
24 stop	12.28873	14.35294	12.691	13.68885	12.29009	11.85395	12.86093	12.49054	0.959971
25 stop	30.80723	31.03392	39.40031	40.25382	34.5299	31.01426	34.50657	32.78191	4.357082
26 stop	30.07647	29.66918	29.74121	29.0187	28.54227	27.07401	29.02031	29.34394	1.102071
27 stop	16.8803	18.56899	16.19341	13.54204	15.55079	15.63474	16.06171	15.91408	1.659273
28 stop	37.13387	34.86412	35.47063	34.1813	32.85592	28.76301	33.87814	34.52271	2.877826
29 stop	53.30052	54.42541	56.84124	62.69524	68.25437	61.07753	59.43239	58.95939	5.666811
30 stop	33.36469	34.80533	32.35794	21.28469	15.89951	17.01645	25.7881	26.82131	8.681991
31 stop	18.14485	18.81833	17.962	14.29412	14.97819	14.67964	16.47952	16.4701	2.035221
32 stop	29.45035	28.74835	28.89986	30.01421	27.54527	26.26672	28.48746	28.82411	1.364968
33 stop	13.31688	14.7367	17.20581	16.49106	17.54929	14.63198	15.65529	15.61388	1.676246
34 stop	19.87593	19.42957	20.02487	10.03776	10.57553	11.01304	15.15945	15.22131	5.071255
11 eyes opened	36.82474	37.98279	36.81911	21.6415	19.88627	16.50431	28.27645	29.23031	9.932415
12 eyes opened	23.77455	23.58665	21.56289	18.27062	13.05712	17.8066	19.6764	19.91676	4.121432
13 eyes opened	21.4077	20.9273	18.63899	11.7651	6.803013	6.803013	14.39085	15.20204	6.812534
14 eyes opened	53.07003	55.22708	51.20573	46.35038	47.69447	42.66953	49.36954	49.4501	4.651608
15 eyes opened	39.31977	44.14567	39.79155	22.51793	28.35262	24.72863	33.14269	33.8362	9.056001
16 eyes opened	12.88264	13.52933	11.03849	12.18051	7.473993	10.54089	11.27431	11.6095	2.169283
17 eyes opened	31.5398	31.27116	35.38817	28.14	24.53765	22.62007	28.91614	29.70558	4.769295
18 eyes opened	66.73754	72.5319	73.72595	62.8315	63.73208	58.39883	66.3263	65.23481	5.919828
19 eyes opened	28.48118	26.7605	21.83174	18.25893	14.92835	15.4899	20.95843	20.04533	5.737114
20 eyes opened	50.45058	51.32604	20.22062	45.48096	7.6888	7.688799	30.47597	32.85079	20.98829
21 eyes opened	65.91637	65.57383	69.86873	11.91231	30.60403	36.44	46.71921	51.00691	23.81936
22 eyes opened	29.79589	40.78132	39.53078	26.71113	15.70488	24.40612	29.48835	28.25351	9.507914
23 eyes opened	16.2169	10.84845	15.73826	15.87162	15.47842	17.25315	15.23447	15.80494	2.235921
24 eyes opened	18.32664	18.13668	15.79093	16.47942	14.5778	15.66381	16.49588	16.13517	1.477331

25	eyes opened	57.64729	56.51617	51.67059	56.08235	45.51575	47.17165	52.43397	53.87647	5.163147
26	eyes opened	35.64493	34.9162	35.21076	32.20155	33.23344	31.72927	33.82269	34.07482	1.66137
27	eyes opened	21.96808	23.75918	22.63516	20.5241	19.44001	17.15044	20.91283	21.24609	2.39406
28	eyes opened	40.58305	36.70162	37.13716	32.52566	32.63061	25.93451	34.2521	34.66612	5.083243
29	eyes opened	63.40863	60.8193	63.63084	73.29092	67.75085	56.45195	64.22541	63.51973	5.790701
30	eyes opened	52.13607	54.77295	54.16117	30.82372	24.70396	23.30958	39.98457	41.47989	15.24995
31	eyes opened	22.46689	25.14907	23.96269	11.13521	12.18218	12.21844	17.85241	17.34266	6.646567
32	eyes opened	28.50538	26.98707	27.47695	32.13695	28.04563	25.25236	28.06739	27.76129	2.28883
33	eyes opened	16.18004	16.22208	20.34561	19.68363	18.91303	15.24479	17.76486	17.56756	2.140224
34	eyes opened	24.94128	26.1229	24.20117	12.96135	9.895231	12.93256	18.50908	18.58126	7.318697
11	eyes closed	51.60507	54.91107	52.99913	55.71915	47.64658	42.83196	50.95216	52.3021	4.896491
12	eyes closed	26.11778	23.22685	17.48526	22.93454	14.61008	21.09037	20.91081	22.01245	4.196138
13	eyes closed	53.37911	51.20842	60.97978	47.79822	6.803013	6.803013	37.82859	49.50332	24.41877
14	eyes closed	52.09982	55.85984	53.74646	48.89069	54.26746	45.21171	51.67933	52.92314	3.955543
15	eyes closed	62.39187	28.91862	60.70664	58.94856	53.20988	39.71686	50.64874	56.07922	13.46264
16	eyes closed	26.66381	39.43365	41.98971	27.07541	31.83605	28.31892	32.55293	30.07749	6.626052
17	eyes closed	40.87423	35.94081	30.59329	28.22465	15.4639	13.58	27.44614	29.40897	10.94374
18	eyes closed	71.58347	75.02413	77.6039	75.24202	78.6591	72.4363	75.09149	75.13308	2.77213
19	eyes closed	53.25941	60.1374	51.69625	32.99709	36.05691	29.78278	43.98831	43.87658	12.58308
20	eyes closed	58.61087	49.82015	15.73152	35.87696	16.19664	16.19656	32.07212	26.0368	18.99923
21	eyes closed	64.89203	60.98189	59.87933	16.96939	55.23286	53.07319	51.83811	57.55609	17.59291
22	eyes closed	23.58164	69.06334	42.65645	32.63574	24.80315	29.54195	37.04705	31.08885	17.10862
23	eyes closed	40.61002	7.248993	36.61933	42.12515	32.01623	45.54062	34.02672	38.61467	13.9234
24	eyes closed	29.60145	33.42619	26.01781	28.52138	24.33404	15.04588	26.15779	27.26959	6.278458
25	eyes closed	53.78436	54.56909	59.31627	65.89739	60.3092	60.41757	59.04898	59.81273	4.432131
26	eyes closed	34.36281	32.4625	35.14192	27.67637	28.24211	25.13109	30.5028	30.3523	4.055028
27	eyes closed	23.48886	25.18594	19.7665	22.84193	14.01999	19.30855	20.76863	21.30421	3.997275
28	eyes closed	64.54673	62.23493	68.65919	76.53968	82.44006	12.60187	61.17041	66.60296	24.9645
29	eyes closed	47.07292	50.60498	49.13529	54.29325	67.77819	46.57157	52.57603	49.87013	7.951303
30	eyes closed	72.11824	70.1333	62.28693	49.14149	26.38968	34.40176	52.4119	55.71421	19.03744
31	eyes closed	37.3071	39.4622	36.88579	21.18316	25.01038	21.76759	30.26937	30.94809	8.488949
32	eyes closed	53.74238	51.41492	50.59326	68.91674	71.99871	60.37177	59.5063	57.05707	9.203274
33	eyes closed	14.51959	14.61066	39.81741	47.19571	24.52056	11.91759	25.43025	19.56561	14.83409

34	eyes closed	48.96253	43.12581	60.66173	23.28054	22.87211	20.13948	36.50703	33.20317	16.79967
