# Supplementary material for: Signal Quality Evaluation of Emerging EEG Devices
Source: Front Physiol. 2018 Feb 14;9:98. doi: 10.3389/fphys.2018.00098 (PMC5817086; doi:10.3389/fphys.2018.00098)
Supplement: Supplementary file 1 [file DataSheet1.ZIP › F-Band_gLADYbird_theta.pdf]

g.LADYbird (tasks: 0-back, stop)
frontal theta

Vp	Task	Fp1	Fp2	F3	Fz	F4	mean	median	std
11	0-back	17.68937	18.30141	15.17517	14.70421	11.62771	15.49957	15.17517	2.663391
12	0-back	37.23883	35.66272	27.52551	24.12468	26.4021	30.19077	27.52551	5.870956
13	0-back	25.42738	26.49053	17.80268	19.00671	15.66015	20.87749	19.00671	4.805796
14	0-back	21.75031	20.05205	28.03905	33.10713	29.33894	26.4575	28.03905	5.436197
15	0-back	38.37296	37.72552	35.67485	35.26585	35.18262	36.44436	35.67485	1.494482
16	0-back	25.61881	25.8597	26.50123	26.87947	25.58827	26.0895	25.8597	0.574275
17	0-back	24.94499	24.63484	21.66802	22.08222	21.53317	22.97265	22.08222	1.674816
18	0-back	22.64536	22.71872	30.04846	30.4428	31.0275	27.37657	30.04846	4.299708
19	0-back	27.91228	30.18171	21.36089	22.3824	21.41219	24.64989	22.3824	4.113573
20	0-back	27.80437	24.32193	35.11394	35.33458	33.04494	31.12395	33.04494	4.863812
21	0-back	23.04645	23.22164	28.92373	29.16703	23.73606	25.61898	23.73606	3.139294
22	0-back	18.74029	28.4342	20.393	21.79683	22.14147	22.30116	21.79683	3.681539
23	0-back	24.19005	21.97246	28.15466	30.73015	29.50406	26.91028	28.15466	3.696259
24	0-back	32.83985	25.26378	52.54901	32.00501	29.49112	34.42975	32.00501	10.54893
25	0-back	17.04824	16.64429	21.17324	22.66374	20.99105	19.70411	20.99105	2.692123
26	0-back	43.84347	41.72622	34.08722	32.77192	31.10383	36.70653	34.08722	5.697928
27	0-back	42.99739	42.339	32.50265	34.12219	32.44018	36.88028	34.12219	5.331553
28	0-back	33.62002	28.40525	26.19999	25.89805	21.12933	27.05053	26.19999	4.530609
29	0-back	39.10884	38.18865	26.87061	23.60552	25.34223	30.62317	26.87061	7.423956
30	0-back	24.07825	25.90582	24.51084	25.41638	24.19449	24.82116	24.51084	0.801828
31	0-back	34.18825	26.40982	28.82482	28.7305	24.5032	28.53132	28.7305	3.63501
32	0-back	41.60654	41.08445	25.51866	33.22766	32.38689	34.76484	33.22766	6.712967
33	0-back	28.90449	28.51003	31.0042	32.55483	30.49714	30.29414	30.49714	1.640908
34	0-back	35.41067	35.71764	33.91681	34.51263	35.02083	34.91572	35.02083	0.717531
11	stop	19.82224	19.35155	14.33391	18.05855	14.10609	17.13447	18.05855	2.738965
12	stop	35.79916	34.40842	28.95018	26.48051	27.31587	30.59083	28.95018	4.243008
13	stop	28.03556	28.60827	25.23787	27.69185	22.21858	26.35843	27.69185	2.647952
14	stop	25.89337	25.22469	27.12259	29.75577	26.99228	26.99774	26.99228	1.731017
15	stop	47.74786	47.34632	42.67222	41.62884	39.80214	43.83948	42.67222	3.539878

16	stop	25.23236	24.96567	27.71855	28.41579	26.33981	26.53444	26.33981	1.511285
17	stop	35.93129	35.42223	28.18122	27.93263	25.62936	30.61935	28.18122	4.726185
18	stop	31.37593	31.78072	31.29836	31.2138	30.86486	31.30673	31.29836	0.329167
19	stop	39.14324	40.86986	28.83171	29.32443	27.80319	33.19448	29.32443	6.272485
20	stop	31.27857	26.50655	39.26008	40.00297	37.4414	34.89792	37.4414	5.81107
21	stop	27.52926	27.404	28.77489	30.31722	25.18961	27.843	27.52926	1.892206
22	stop	24.1777	29.36391	24.00946	27.39584	27.42006	26.47339	27.39584	2.315348
23	stop	24.32377	20.10674	26.37607	29.12934	28.12787	25.61276	26.37607	3.579543
24	stop	30.48498	25.93311	13.41161	30.6778	28.51549	25.8046	28.51549	7.186892
25	stop	27.16858	20.49394	27.46257	28.52834	27.21514	26.17371	27.21514	3.222558
26	stop	42.21741	42.38026	35.61758	34.84229	34.54241	37.91999	35.61758	4.016939
27	stop	32.62617	31.32174	30.42506	30.79	30.05517	31.04363	30.79	1.000725
28	stop	24.85548	24.61893	23.16018	23.85133	22.04389	23.70596	23.85133	1.144159
29	stop	40.71993	40.66311	32.54378	30.42788	31.77185	35.22531	32.54378	5.0471
30	stop	27.25821	26.94191	22.5457	22.62372	23.23475	24.52086	23.23475	2.372185
31	stop	35.86692	28.99096	32.41992	31.49751	27.67188	31.28944	31.49751	3.187646
32	stop	36.57776	36.15659	26.30905	31.64515	31.25123	32.38796	31.64515	4.199825
33	stop	24.57566	24.01095	27.86492	29.48961	26.55269	26.49876	26.55269	2.274972
34	stop	33.20451	33.99386	34.34715	35.22794	34.81356	34.3174	34.34715	0.777712
