# Supplementary material for: Signal Quality Evaluation of Emerging EEG Devices
Source: Front Physiol. 2018 Feb 14;9:98. doi: 10.3389/fphys.2018.00098 (PMC5817086; doi:10.3389/fphys.2018.00098)
Supplement: Supplementary file 1 [file DataSheet1.ZIP › F-Band_gSAHARA_alpha.pdf]

g.SAHARA (tasks: 0-back, stop, rest measurements)
parietal alpha

Vp	Task	P3	Pz	P4	PO7	PO8	Oz	mean	median	std
11	0-back	1.078332	21.92892	19.13219	13.09513	14.09005	9.850378	13.19583	13.59259	7.3531315
12	0-back	18.31064	20.01321	16.98708	16.61594	14.42085	14.64247	16.8317	16.80151	2.14405701
13	0-back	10.13287	11.96903	9.13597	10.84672	30.07081	30.19502	17.0584	11.40788	10.1696409
14	0-back	43.78525	43.04372	27.20456	28.43518	22.39193	17.55388	30.40242	27.81987	10.791218
15	0-back	18.1461	20.75796	20.43764	18.22056	18.32964	18.78809	19.11333	18.55887	1.1756799
16	0-back	14.28423	14.31457	14.25117	14.3117	14.1045	14.37131	14.27291	14.29796	0.09152661
17	0-back	14.85485	14.06451	22.91611	15.22686	9.858808	12.66056	14.93028	14.45968	4.37008298
18	0-back	49.16923	56.0271	58.75613	35.51166	34.78053	19.68921	42.32231	42.34045	14.9685843
19	0-back	12.82404	16.68948	14.83394	15.4093	13.57447	14.05511	14.56439	14.44452	1.38352198
20	0-back	34.82232	36.60168	32.44199	15.42622	14.56749	15.42892	24.88144	23.93545	10.7561685
21	0-back	15.15507	15.30084	15.28794	15.08617	2.15375	0.329342	10.55219	15.12062	7.23548183
22	0-back	28.27331	37.70357	31.76273	21.84166	13.35081	15.96649	24.81643	25.05748	9.4330341
23	0-back	14.79812	13.39464	15.36281	14.94978	16.09897	15.09648	14.95013	15.02313	0.88929565
24	0-back	12.72761	14.20642	13.13917	14.10251	13.88719	12.22131	13.3807	13.51318	0.81097574
25	0-back	19.01136	14.42094	15.01525	15.77326	0.111094	14.10829	13.07336	14.71809	6.59132061
26	0-back	20.64606	19.49533	18.59663	24.88314	20.10756	20.09533	20.63734	20.10145	2.19408533
27	0-back	13.90463	14.92862	14.88865	14.66383	13.21249	14.05135	14.27493	14.35759	0.67395789
28	0-back	20.47942	19.05874	16.93703	25.297	3.016823	17.24395	17.00549	18.15135	7.493697
29	0-back	35.76487	22.75396	27.82725	23.17561	21.94337	21.838	25.55051	22.96479	5.47305517
30	0-back	32.88107	42.96699	31.48773	25.4827	21.87792	23.33857	29.6725	28.48522	7.85826926
31	0-back	13.44022	15.0525	15.08097	15.46639	14.50421	14.7005	14.70746	14.8765	0.70452605
32	0-back	19.63152	16.69948	15.86286	17.39113	16.13606	15.98932	16.95173	16.41777	1.42934526
33	0-back	15.26199	16.59494	13.42845	14.5238	16.62342	17.34687	15.62991	15.92846	1.48805709
34	0-back	13.0742	12.88221	12.22246	12.26421	12.26293	12.53441	12.54007	12.39931	0.36225953
11	stop	23.09237	26.04947	24.3076	16.76913	24.89109	8.786147	20.6493	23.69999	6.66778108
12	stop	14.42549	28.2592	11.93884	11.85544	9.228614	10.30103	14.33477	11.89714	7.04456337
13	stop	10.10983	11.65601	8.677052	10.04364	5.464071	5.555633	8.584373	9.360347	2.56162446
14	stop	30.25552	31.85037	24.99378	24.94045	22.41741	15.25345	24.95183	24.96711	5.93637332
15	stop	18.33701	17.47912	16.31185	18.12611	18.20618	19.70359	18.02731	18.16614	1.11273488

16	stop	15.00429	15.00326	13.26948	15.02788	15.01204	15.13935	14.74272	15.00817	0.72358721
17	stop	15.71558	13.59611	16.2581	15.88064	13.15114	14.05254	14.77568	14.88406	1.33081642
18	stop	45.86462	54.03959	62.31482	37.77198	41.72317	26.73026	44.74074	43.79389	12.4789964
19	stop	14.66836	15.57128	12.82176	16.04797	12.94597	13.48187	14.2562	14.07511	1.37721935
20	stop	40.11686	42.93621	39.33517	15.87263	16.35742	16.10204	28.45339	27.84629	13.574574
21	stop	16.54715	14.39606	13.73178	14.14339	13.00445	13.90834	14.28853	14.02586	1.20311065
22	stop	26.02749	28.02061	31.43837	16.79625	15.19571	15.57022	22.17477	21.41187	7.1566398
23	stop	14.90289	12.91863	14.75772	14.77243	15.71639	15.27684	14.72415	14.83766	0.95763668
24	stop	12.26243	15.41996	12.83409	14.96353	14.21472	12.44016	13.68915	13.52441	1.35812894
25	stop	16.44661	13.56686	15.28276	15.44827	13.82585	13.62171	14.69868	14.55431	1.19674663
26	stop	20.23043	19.34223	19.30146	22.6478	20.61546	21.35993	20.58288	20.42294	1.27860986
27	stop	13.89497	14.97573	14.26795	13.35934	12.41332	12.8616	13.62882	13.62716	0.94150891
28	stop	21.38193	21.21222	19.30063	24.33134	13.41831	9.193717	18.13969	20.25643	5.69137421
29	stop	31.58586	19.17631	26.4098	20.21761	19.9602	20.74443	23.0157	20.48102	4.94029187
30	stop	28.75306	39.25603	32.86519	20.94809	19.37947	19.27259	26.74574	24.85057	8.26688562
31	stop	14.6996	14.07507	16.08161	14.42403	14.29944	13.4471	14.50447	14.36173	0.88049929
32	stop	20.08312	19.439	14.41255	17.26514	15.65082	17.06181	17.31874	17.16347	2.16490633
33	stop	4.224272	12.15144	14.71375	14.1004	14.66451	15.38569	12.54001	14.38245	4.22075449
34	stop	16.75253	14.45289	15.2723	14.87513	13.45582	13.71799	14.75444	14.66401	1.19387453
11	eyes opened	12.52769	17.86115	22.33891	14.85271	9.09059	7.659307	14.05506	13.6902	5.5068894
12	eyes opened	19.24737	19.19786	20.03843	15.52825	14.64239	16.80476	17.57651	18.00131	2.23076737
13	eyes opened	12.45579	12.15864	12.65586	11.73054	14.30994	6.803013	11.68563	12.30722	2.54900754
14	eyes opened	46.37888	49.69776	37.59169	38.60008	33.81606	16.1253	37.03496	38.09589	11.8151614
15	eyes opened	17.13546	21.64885	18.78027	18.31192	23.33585	23.70373	20.48602	20.21456	2.78172266
16	eyes opened	16.40792	16.59346	16.5236	16.57496	16.64769	15.69219	16.40664	16.54928	0.3592937
17	eyes opened	13.72316	13.90095	18.68829	23.98879	13.79556	18.18239	17.04652	16.04167	4.09118031
18	eyes opened	61.50712	67.18703	69.42897	56.43938	56.82252	48.19834	59.93056	59.16482	7.80839057
19	eyes opened	15.76944	15.77034	15.13303	15.38937	15.48784	15.75566	15.55095	15.62175	0.26173673
20	eyes opened	34.97333	37.89604	38.75459	17.43601	18.40018	17.10281	27.42716	26.68675	10.7958748
21	eyes opened	41.95836	19.08084	25.66227	17.05922	15.44925	17.26069	22.7451	18.17076	10.0670119
22	eyes opened	21.59808	27.57647	30.48026	17.53815	17.33266	14.49803	21.50394	19.56811	6.31931225
23	eyes opened	13.68059	13.55615	13.85258	14.07419	14.76771	14.58979	14.08683	13.96338	0.49360737
24	eyes opened	14.63192	14.95905	12.33431	13.56934	11.26941	14.39586	13.52665	13.9826	1.4524137
