# Supplementary material for: Signal Quality Evaluation of Emerging EEG Devices
Source: Front Physiol. 2018 Feb 14;9:98. doi: 10.3389/fphys.2018.00098 (PMC5817086; doi:10.3389/fphys.2018.00098)
Supplement: Supplementary file 1 [file DataSheet1.ZIP › F-Band_gSAHARA_theta.pdf]

g.SAHARA (tasks: 0-back, stop)
frontal theta

Vp	Task	Fp1	Fp2	F3	Fz	F4	mean	median	std
11	0-back	15.68666	16.79402	12.01607	15.38409	11.51234	14.27864	15.38409	2.36132
12	0-back	24.64969	29.38335	23.78312	20.3017	26.05564	24.8347	24.64969	3.31169
13	0-back	25.18799	26.5188	18.58765	16.74066	16.35313	20.67765	18.58765	4.82264
14	0-back	26.94188	26.49109	26.39827	28.27927	25.00465	26.62303	26.49109	1.176
15	0-back	37.61615	34.35134	26.21563	26.09918	30.36845	30.93015	30.36845	5.05687
16	0-back	24.29199	25.35577	18.859	18.89456	18.88947	21.25816	18.89456	3.27673
17	0-back	27.94435	24.46345	20.30874	19.19703	20.18394	22.4195	20.30874	3.69253
18	0-back	28.93228	30.04831	25.89684	22.75496	27.33919	26.99432	27.33919	2.84509
19	0-back	20.95317	23.41728	20.48639	23.50015	22.77174	22.22575	22.77174	1.41305
20	0-back	33.46267	33.08421	37.86207	43.33033	39.86685	37.52122	37.86207	4.34519
21	0-back	15.59142	24.7534	29.23296	29.72728	23.45768	24.55255	24.7534	5.70581
22	0-back	29.45101	30.16184	24.71587	22.38117	22.52378	25.84673	24.71587	3.73972
23	0-back	29.32254	25.51857	22.15475	19.47385	27.88433	24.87081	25.51857	4.05657
24	0-back	28.95167	21.75208	23.59691	24.26888	19.66266	23.64644	23.59691	3.4639
25	0-back	26.38309	24.20512	27.44227	26.18177	35.39106	27.92066	26.38309	4.33673
26	0-back	41.14441	41.72723	29.09396	24.62994	30.73786	33.46668	30.73786	7.61313
27	0-back	38.29687	37.89146	27.37989	29.54947	29.51266	32.52607	29.54947	5.16027
28	0-back	34.1337	32.63583	26.41342	18.55748	25.53438	27.45496	26.41342	6.23269
29	0-back	32.14276	29.99834	20.36609	21.74788	20.94392	25.0398	21.74788	5.57888
30	0-back	19.37008	19.04314	20.50652	21.52606	20.99449	20.28806	20.50652	1.05734
31	0-back	37.75778	34.16055	23.84808	22.43856	19.75512	27.59202	23.84808	7.88163
32	0-back	42.10943	39.53136	26.38151	24.43731	26.33885	31.75969	26.38151	8.35828
33	0-back	22.79455	24.56499	22.11746	16.95961	24.54378	22.19608	22.79455	3.11883
34	0-back	29.08026	32.32344	27.44201	28.55042	28.33788	29.1468	28.55042	1.87161
11	stop	11.09356	13.57211	24.26328	14.64387	9.226312	14.55983	13.57211	5.82239
12	stop	32.98811	33.55923	23.55537	23.11473	27.38143	28.11977	27.38143	4.99294
13	stop	26.95353	28.35784	20.23961	22.36639	23.39166	24.26181	23.39166	3.33732
14	stop	27.20787	28.94363	25.79211	25.79921	24.59736	26.46804	25.79921	1.6643
15	stop	52.47572	52.61128	42.98033	44.65654	44.97396	47.53957	44.97396	4.63057

16	stop	23.1694	24.73221	21.20795	20.28748	20.68064	22.01553	21.20795	1.87963
17	stop	30.39768	28.77641	22.12893	22.17936	22.6629	25.22906	22.6629	4.02477
18	stop	27.29068	28.603	27.74433	25.91939	27.71987	27.45545	27.71987	0.98195
19	stop	26.92339	35.06374	22.31957	19.76422	24.31172	25.67653	24.31172	5.8686
20	stop	32.48655	31.30016	37.61032	41.02824	37.62868	36.01079	37.61032	4.02993
21	stop	25.51459	29.24724	29.06993	31.14501	23.14589	27.62453	29.06993	3.22592
22	stop	37.38243	37.81525	30.81499	29.72722	30.02416	33.15281	30.81499	4.08095
23	stop	23.86381	31.63216	21.07701	21.68757	29.58016	25.56814	23.86381	4.76976
24	stop	37.3303	30.17651	34.37256	31.72912	28.14169	32.35004	31.72912	3.59561
25	stop	29.92299	32.2996	23.36019	29.65356	29.85506	29.01828	29.85506	3.34303
26	stop	38.32663	40.02118	31.27571	28.92456	25.40642	32.7909	31.27571	6.21885
27	stop	33.15752	31.11736	28.56463	27.5175	27.85895	29.64319	28.56463	2.41811
28	stop	27.282	27.05186	26.50904	20.30718	21.98564	24.62715	26.50904	3.24456
29	stop	33.77363	33.54174	23.29434	25.67867	23.44347	27.94637	25.67867	5.29918
30	stop	13.85498	14.62414	15.79596	19.9393	17.39805	16.32248	15.79596	2.42324
31	stop	38.10052	37.53535	32.32985	27.91134	30.52706	33.28082	32.32985	4.43422
32	stop	38.85005	36.6294	27.72779	23.63665	29.8536	31.3395	29.8536	6.30432
33	stop	25.4191	25.05975	22.91635	24.226	33.5471	26.23366	25.05975	4.2001
34	stop	27.39301	30.17717	26.29618	26.13152	25.55544	27.11066	26.29618	1.83875
