# Supplementary material for: Signal Quality Evaluation of Emerging EEG Devices
Source: Front Physiol. 2018 Feb 14;9:98. doi: 10.3389/fphys.2018.00098 (PMC5817086; doi:10.3389/fphys.2018.00098)
Supplement: Supplementary file 1 [file DataSheet1.ZIP › F-Band_Jellyfish_theta.pdf]

Jellyfish (tasks: 0-back, stop)<br>frontal theta								
Vp	Task	AF8	Fp2	Fp1	AF7	mean	median	std
11	0-back	32.0411	32.81329	32.43496	31.84996	32.28483	32.23803	0.428299
12	0-back	33.27567	33.53272	28.45086	32.43185	31.92278	32.85376	2.361891
13	0-back	27.94036	32.23408	39.64414	28.471	32.07239	30.35254	5.397569
14	0-back	30.2861	38.28894	31.37775	25.56728	31.38002	30.83193	5.25096
15	0-back	38.21672	33.93098	39.22526	38.87482	37.56195	38.54577	2.456482
16	0-back	27.85275	32.99509	30.89724	30.67796	30.60576	30.7876	2.111725
17	0-back	26.1839	28.77931	47.07084	29.31902	32.83827	29.04916	9.586567
18	0-back	23.0717	23.74282	22.11192	22.70736	22.90845	22.88953	0.682575
19	0-back	26.74654	29.21556	35.58349	28.56736	30.02824	28.89146	3.848158
20	0-back	35.1031	38.05287	34.3726	26.22792	33.43912	34.73785	5.063872
21	0-back	31.87458	40.72317	34.22876	39.10111	36.48191	36.66494	4.129274
22	0-back	35.08952	35.00618	27.24342	59.20418	39.13583	35.04785	13.87557
23	0-back	13.48836	32.99574	32.85582	9.585323	22.23131	23.17209	12.45142
24	0-back	24.81583	32.46479	34.5647	34.90568	31.68775	33.51474	4.706695
25	0-back	21.99099	27.73904	34.13085	22.61047	26.61784	25.17476	5.632326
26	0-back	48.23691	48.04822	42.14523	45.51114	45.98538	46.77968	2.845838
27	0-back	39.45116	39.61144	41.0221	40.08967	40.04359	39.85056	0.70648
28	0-back	20.13065	37.27853	38.86764	42.82463	34.77536	38.07308	10.03778
29	0-back	36.5552	36.63529	36.58984	40.62725	37.60189	36.61256	2.017173
30	0-back	11.47257	20.1197	36.12626	13.41947	20.2845	16.76959	11.19177
31	0-back	33.98608	36.81038	37.70093	37.49053	36.49698	37.15045	1.716535
32	0-back	60.19743	41.6487	35.78387	35.4459	43.26897	38.71628	11.63937
33	0-back	31.65	40.13671	37.46624	41.83658	37.77238	38.80148	4.460382
34	0-back	35.00564	38.45543	34.99545	19.8904	32.08673	35.00055	8.292398
11	stop	37.24966	38.79077	36.87978	36.02218	37.2356	37.06472	1.157258
12	stop	35.74748	36.31137	37.81783	35.37319	36.31247	36.02942	1.075109
13	stop	34.94759	35.44937	33.91905	33.76875	34.52119	34.43332	0.810775
14	stop	37.30075	36.50851	34.36313	31.14759	34.83	35.43582	2.750763
15	stop	50.83673	37.62489	51.57378	51.65064	47.92151	51.20526	6.874211

16 stop	28.84452	29.49261	28.32366	24.42081	27.7704	28.58409	2.28368
17 stop	30.81839	31.73397	36.07894	33.17627	32.95189	32.45512	2.299561
18 stop	28.57701	28.97931	28.84275	28.47324	28.71808	28.70988	0.233544
19 stop	31.3648	33.53528	40.18544	32.96978	34.51383	33.25253	3.891235
20 stop	30.67778	39.09108	32.46626	30.58828	33.20585	31.57202	4.017701
21 stop	35.99428	37.61433	43.00093	38.04291	38.66311	37.82862	3.023461
22 stop	24.11541	34.88397	32.41926	28.05652	29.86879	30.23789	4.762447
23 stop	32.42293	32.69623	35.7481	38.93204	34.94983	34.22216	3.052823
24 stop	32.41241	35.29512	34.77265	31.67749	33.53942	33.59253	1.764488
25 stop	53.45837	29.77495	30.06645	35.23451	37.13357	32.65048	11.16839
26 stop	43.32594	43.24275	40.056	40.34243	41.74178	41.79259	1.785355
27 stop	31.449	31.84215	32.4134	34.05471	32.43982	32.12777	1.1471
28 stop	31.24584	48.14439	38.54622	38.6569	39.14834	38.60156	6.927783
29 stop	39.80511	40.86031	44.93934	43.08662	42.17284	41.97346	2.296183
30 stop	16.80958	18.28006	27.24229	17.71893	20.01272	17.9995	4.857651
31 stop	26.97739	36.17086	35.58727	36.0638	33.69983	35.82553	4.488801
32 stop	31.01117	41.89069	42.15662	42.05598	39.27861	41.97334	5.512722
33 stop	25.76716	28.58512	28.62858	31.01484	28.49893	28.60685	2.146051
34 stop	40.87401	38.20197	39.97591	30.52821	37.39503	39.08894	4.710574
