# Supplementary material for: Signal Quality Evaluation of Emerging EEG Devices
Source: Front Physiol. 2018 Feb 14;9:98. doi: 10.3389/fphys.2018.00098 (PMC5817086; doi:10.3389/fphys.2018.00098)
Supplement: Supplementary file 1 [file DataSheet1.ZIP › F-Band_Trilobite_alpha.pdf]

Trilobite (tasks: 0-back, stop, rest measurements)
parietal alpha

Vp	Task	P8	P4	Pz	P3	P7	O2	Oz	O1	mean	median	std
11	0-back	10.58146	7.466221	8.539721	11.38605	9.377427	18.36175	6.961489	21.07616	11.71879	9.979445	5.201624
12	0-back	18.05047	16.86164	19.58545	18.72954	19.29396	17.21442	14.34172	17.8575	17.74184	17.95398	1.669297
13	0-back	28.05213	17.81215	17.32996	16.86136	16.23485	3.951743	14.05472	16.02855	16.29068	16.54811	6.539693
14	0-back	31.10345	17.20743	16.50594	17.81347	30.05104	20.63776	15.25056	7.986673	19.56954	17.51045	7.698789
15	0-back	28.51006	18.75188	16.33971	17.32987	19.10049	35.50987	18.95558	31.51856	23.252	19.02803	7.416539
16	0-back	15.26323	15.14903	15.27638	15.39642	17.10758	18.56561	14.96057	17.28743	16.12578	15.3364	1.340327
17	0-back	18.79269	16.49292	18.01884	16.55716	10.33737	10.36362	14.16314	15.1422	14.98349	15.81756	3.209494
18	0-back	27.15692	79.62583	11.25342	39.18704	18.64597	44.92914	26.33773	32.68292	34.97737	29.91992	20.97908
19	0-back	18.85022	17.90431	18.3909	17.51047	17.49506	13.46868	16.93183	13.0958	16.70591	17.50276	2.194378
20	0-back	18.35466	17.30034	15.49461	15.91683	17.98982	17.55276	15.3589	22.73026	17.58727	17.42655	2.373064
21	0-back	17.71919	17.03238	16.41903	17.46667	18.17663	17.06763	14.49663	14.74689	16.64063	17.05001	1.35081
22	0-back	16.89475	17.26039	15.8238	15.71169	17.5109	27.19195	23.07732	22.40878	19.48495	17.38565	4.209646
23	0-back	16.28897	15.09095	15.95655	16.77941	16.71291	16.60957	13.75284	12.02365	15.40186	16.12276	1.707041
24	0-back	16.6172	16.31617	15.88641	17.20612	17.32976	14.26588	14.87584	15.79383	16.0364	16.10129	1.070354
25	0-back	17.01144	15.70617	17.03528	16.98485	17.65367	12.68463	12.03543	13.15894	15.2838	16.34551	2.28546
26	0-back	7.633488	18.23589	17.11184	16.77005	23.57501	14.38962	14.63863	18.88087	16.40442	16.94094	4.561411
27	0-back	15.66463	16.08163	14.84318	15.26084	7.835805	15.73593	4.491276	14.84318	13.09456	15.05201	4.391286
28	0-back	17.0748	17.46458	17.91223	16.81814	17.61518	17.54119	12.90515	15.57585	16.61339	17.26969	1.66297
29	0-back	9.565779	15.61534	14.11262	15.49538	11.32456	13.63192	15.50985	14.12806	13.67294	14.12034	2.179588
30	0-back	10.89204	20.6552	15.06875	18.68948	16.81977	14.06344	15.00072	16.82839	16.00222	15.94426	2.975368
31	0-back	15.58832	14.73703	15.65478	14.4155	14.54547	15.58942	14.73799	16.11058	15.17239	15.16316	0.632902
32	0-back	16.79732	15.23982	16.91934	17.10037	11.36697	16.78757	15.20304	16.92979	15.79303	16.79245	1.946124
33	0-back	12.01345	23.42945	12.28277	17.91123	7.413381	12.01385	16.04911	17.8255	14.86734	14.16594	4.946151
34	0-back	17.57479	16.02035	17.99991	16.85216	16.47202	16.18901	15.74255	5.686881	15.31721	16.33052	3.967039
11	stop	10.74297	9.911485	9.571256	11.34452	2.821297	19.45753	15.63086	22.28199	12.72024	11.04374	6.174678
12	stop	5.420646	18.49227	16.53831	16.39297	8.262748	17.00045	14.08929	15.26856	13.93315	15.83076	4.620688
13	stop	25.8824	9.061702	14.53298	18.23826	17.59868	17.25953	10.43024	16.8724	16.23452	17.06596	5.188577
14	stop	27.73533	17.35062	16.90871	18.60417	25.8035	23.2028	14.90382	16.20037	20.08866	17.97739	4.820238
15	stop	19.00419	13.33323	16.81226	14.17662	15.22543	23.89567	16.94139	26.53034	18.23989	16.87683	4.701064

16 stop	15.60516	16.60849	15.46683	15.74115	17.61685	20.74992	15.95596	15.06923	16.6017	15.84855	1.852617
17 stop	19.13714	17.98887	14.45598	18.71705	18.24628	13.88885	14.72393	15.95407	16.63902	16.97147	2.118963
18 stop	30.89952	54.14552	11.26952	8.183329	20.64204	34.48598	15.82404	34.32295	26.22161	25.77078	15.23035
19 stop	17.82143	16.86162	17.35358	17.93986	16.44384	13.78839	15.77174	14.25169	16.27902	16.65273	1.568863
20 stop	19.67777	18.31683	18.41712	18.66369	18.49566	20.66324	15.84562	27.35345	19.67917	18.57967	3.389894
21 stop	14.24784	15.56759	15.14176	15.69892	16.05109	14.9013	10.38656	17.6186	14.95171	15.35468	2.091931
22 stop	7.147505	15.44875	19.0942	16.64917	18.05415	30.01909	25.76585	27.48452	19.9579	18.57417	7.477265
23 stop	15.08106	15.17428	15.46291	15.03683	15.20812	15.38834	16.69239	11.2836	14.91594	15.1912	1.561411
24 stop	12.98174	13.17381	12.58866	12.4818	14.30052	12.70702	13.44985	14.03144	13.21435	13.07777	0.670194
25 stop	13.242	12.57747	13.91033	11.40483	17.14119	14.65909	12.68872	7.63204	12.90696	12.96536	2.734828
26 stop	19.47435	17.29859	16.40174	18.33678	25.78316	17.46627	4.201603	18.85524	17.22722	17.90153	6.006493
27 stop	14.71481	15.41602	14.65817	16.27574	14.09318	14.71481	15.91957	14.65817	15.05631	14.71481	0.740609
28 stop	20.24428	16.31156	15.79251	16.50342	16.45169	16.55463	12.58562	14.78766	16.15392	16.38163	2.133317
29 stop	10.59917	15.52467	16.30764	16.65531	0.033179	10.63946	15.58634	16.25923	12.70063	15.55551	5.69141
30 stop	7.068355	24.25167	17.52458	18.1415	8.994833	4.55614	16.26249	15.58071	14.04753	15.9216	6.596067
31 stop	17.27004	16.94111	16.99082	15.60934	8.680218	17.27275	16.94892	16.97491	15.83601	16.96192	2.939094
32 stop	17.25206	16.84674	17.72713	17.00607	7.413383	17.24694	16.93422	17.74103	16.02095	17.1265	3.49418
33 stop	13.09842	8.940248	17.00167	16.4112	12.19626	13.09361	15.1874	17.1968	14.1407	14.14291	2.848156
34 stop	16.03001	16.64898	16.30042	18.14533	4.860474	17.86605	17.30639	14.35618	15.18923	16.4747	4.339782
11 eyes opened	14.77382	14.50178	14.94045	18.19738	17.40429	20.55635	17.49136	21.5162	17.4227	17.44782	2.642823
12 eyes opened	18.16424	14.05216	13.67448	18.23904	22.64288	17.25246	12.35092	16.89791	16.65926	17.07518	3.278521
13 eyes opened	25.58726	16.92328	16.4847	16.32882	14.37189	15.05686	15.76189	17.62186	17.26707	16.40676	3.515165
14 eyes opened	27.50863	16.86194	15.49977	17.15394	22.78544	19.01818	18.57248	17.41291	19.35166	17.9927	3.940084
15 eyes opened	28.97264	16.75701	19.1173	16.7121	25.9021	38.35948	25.11658	43.16857	26.76322	25.50934	9.794697
16 eyes opened	12.77343	13.2586	13.51452	14.1459	6.509581	18.05268	10.57383	16.94973	13.22229	13.38656	3.59449
17 eyes opened	12.51127	7.017733	14.46257	10.52094	16.9951	12.60507	10.50725	23.43297	13.50661	12.55817	4.977599
18 eyes opened	10.93592	60.47372	33.17569	44.0566	25.98666	65.04576	32.87497	65.0782	42.20344	38.61615	19.96936
19 eyes opened	16.816	17.18728	8.285141	14.86651	15.154	13.29339	16.43248	15.39591	14.67884	15.27496	2.866283
20 eyes opened	17.18311	16.03011	13.59641	16.21814	17.56355	26.07057	15.79844	29.7964	19.03209	16.70063	5.706126
21 eyes opened	15.35114	14.79812	15.80576	14.60688	16.63285	15.85235	14.82462	38.97445	18.35577	15.57845	8.358708
22 eyes opened	12.77543	10.99245	14.52664	15.8426	11.93307	7.134674	7.676346	5.577942	10.80739	11.46276	3.683943
23 eyes opened	18.10751	20.66637	16.16314	18.53795	18.96899	16.9441	16.50321	20.27033	18.2702	18.32273	1.675416
24 eyes opened	16.35733	16.55984	14.74224	15.88636	15.88439	15.45612	15.11999	15.85913	15.73317	15.87176	0.605908

25	eyes opened	11.03137	9.676747	12.35077	10.36515	13.20761	16.12092	4.945146	11.43566	11.14167	11.23351	3.201951
26	eyes opened	26.59244	17.81985	15.79925	15.42468	26.16396	26.49835	12.60261	27.7048	21.07574	21.9919	6.231758
27	eyes opened	12.70689	11.85452	13.79055	12.52155	8.992963	12.72074	11.85452	13.87601	12.28972	12.61422	1.532027
28	eyes opened	14.24278	12.91775	13.0075	13.5176	18.64208	20.01893	9.91235	20.6775	15.36706	13.88019	3.901859
29	eyes opened	20.23438	20.22595	17.37281	17.67743	11.0013	20.32279	20.22663	17.37333	18.05433	18.95169	3.165639
30	eyes opened	21.21714	33.13559	14.68744	18.93402	20.76221	23.89522	15.17139	26.89052	21.83669	20.98968	6.12892
31	eyes opened	17.04618	16.20627	16.77588	18.8243	6.984914	17.04618	16.2032	16.77588	15.73285	16.77588	3.628346
32	eyes opened	15.31258	15.97783	15.77698	16.83123	13.13636	15.20244	15.97783	15.78357	15.49985	15.78028	1.075466
33	eyes opened	13.42741	16.91318	17.04731	17.50516	6.984914	13.42741	16.91318	17.04731	14.90824	16.91318	3.607524
34	eyes opened	17.48627	17.04847	18.09477	16.30246	17.44072	17.3546	16.03401	22.19852	17.74498	17.39766	1.918394
11	eyes closed	7.719789	30.74681	15.87514	18.1328	31.74161	41.36453	39.04978	41.01754	28.206	31.24421	12.80084
12	eyes closed	10.08061	11.82939	11.63884	11.04174	10.87299	9.787739	10.55475	10.49339	10.78743	10.71387	0.709341
13	eyes closed	22.92588	14.69361	16.08347	16.51485	16.16896	16.08065	16.56848	16.8665	16.9878	16.34191	2.485663
14	eyes closed	38.02656	16.0401	14.83815	16.0471	23.79563	16.15043	16.36541	20.49678	20.22002	16.25792	7.793803
15	eyes closed	55.2888	18.09522	17.49234	18.24021	50.82291	60.56007	42.97995	71.55178	41.87891	46.90143	21.41313
16	eyes closed	18.36895	17.33103	16.01484	16.5644	10.30182	50.44987	12.31318	29.17396	21.31476	16.94771	13.0282
17	eyes closed	20.0305	9.381572	14.85853	14.6927	23.5252	15.49069	13.43449	14.7245	15.76727	14.79152	4.271567
18	eyes closed	13.07809	6.121331	13.71825	10.19599	26.33538	71.3734	38.28014	69.49822	31.0751	20.02682	26.34509
19	eyes closed	14.93179	14.9038	13.61552	13.85951	17.13755	22.36248	16.12208	23.08111	17.00173	15.52693	3.713193
20	eyes closed	17.70232	16.55427	15.92561	18.12069	17.0573	55.2736	14.53386	51.51039	25.83475	17.37981	17.07379
21	eyes closed	16.85779	16.84823	16.84019	16.89295	18.11016	9.306819	13.73595	10.14024	14.84154	16.84421	3.400346
22	eyes closed	18.21573	13.54772	14.8848	20.44065	16.75329	12.99457	19.22461	9.459585	15.69012	15.81904	3.66242
23	eyes closed	16.6689	16.09993	13.56635	15.05499	14.55799	20.46692	13.86148	37.14757	18.42802	15.57746	7.873435
24	eyes closed	14.2188	14.18986	14.94432	14.84686	13.78566	14.2439	13.13666	12.85646	14.02781	14.20433	0.74115
25	eyes closed	13.33095	11.35588	11.24534	9.968089	10.9836	34.58468	13.50798	42.13061	18.38839	12.34341	12.54489
26	eyes closed	30.96431	12.36549	12.30087	13.11761	27.77723	26.04748	13.92245	25.09467	20.19876	19.50856	7.971193
27	eyes closed	14.47355	14.10588	15.68689	12.92843	9.800297	14.47355	14.10588	15.68773	13.90778	14.28972	1.885383
28	eyes closed	18.063	14.60769	15.16278	15.62762	14.77373	14.82456	13.46084	50.88307	19.67541	14.99367	12.67802
29	eyes closed	14.20438	13.69832	7.921673	14.03668	6.840679	14.1072	13.69832	7.915922	11.5529	13.69832	3.328408
30	eyes closed	5.449912	17.63696	14.43664	16.63666	22.80856	11.80117	13.73825	19.68112	15.27366	15.53665	5.281581
31	eyes closed	19.26066	19.16986	16.08749	20.28031	6.984914	19.26066	20.8876	16.08749	17.25237	19.21526	4.508105
32	eyes closed	17.10004	17.83151	17.58042	17.80005	8.993957	17.10363	17.83151	17.56598	16.47589	17.5732	3.037857
33	eyes closed	16.29007	17.51763	17.32014	18.05625	12.78182	16.2972	17.49356	17.31778	16.63431	17.31896	1.67126

34	eyes closed	17.1085	17.07048	16.37918	17.15041	18.06348	18.3287	15.99356	23.55651	17.95635	17.12946	2.390608
