# Supplementary material for: Signal Quality Evaluation of Emerging EEG Devices
Source: Front Physiol. 2018 Feb 14;9:98. doi: 10.3389/fphys.2018.00098 (PMC5817086; doi:10.3389/fphys.2018.00098)
Supplement: Supplementary file 1 [file DataSheet1.ZIP › F-Band_Trilobite_theta.pdf]

Trilobite (tasks: 0-back, stop)
frontal theta

Vp	Task	Fp2	Fp1	AF4	AF3	F8	F4	Fz	F3	F7	mean	median	std
11	0-back	30.06671	28.80175	28.1911	27.05284	33.67483	18.97526	38.34513	23.84386	23.2887	28.02669	28.1911	5.766091
12	0-back	34.25471	31.20435	15.03628	15.60454	19.46766	18.49962	32.9414	18.93496	28.59975	23.83814	19.46766	7.787933
13	0-back	43.84667	41.68112	49.58921	54.82889	20.99291	21.88186	32.92449	28.17167	26.70441	35.62458	32.92449	12.31592
14	0-back	34.0652	32.30952	17.22255	26.82628	17.79768	18.70442	17.49612	17.32184	28.01248	23.30623	18.70442	6.977255
15	0-back	39.17156	39.74564	16.78979	38.54069	39.09176	19.23626	17.57338	17.56887	39.45235	29.68559	38.54069	11.30529
16	0-back	36.64346	35.81145	32.8048	26.54977	15.60142	36.55752	35.02839	32.61916	20.39454	30.22339	32.8048	7.680823
17	0-back	33.83715	28.95089	18.25011	18.20912	32.2527	19.31801	20.13709	19.82206	29.58142	24.48428	20.13709	6.512711
18	0-back	36.57644	35.88198	37.67709	37.0638	34.05677	11.35075	11.09148	11.26841	33.64829	27.62389	34.05677	12.35838
19	0-back	45.86747	41.38099	34.14258	9.24748	14.69174	19.63819	38.9412	19.18254	19.0021	26.89937	19.63819	13.2393
20	0-back	41.3182	40.83378	17.44063	17.48874	20.3738	21.37363	43.19302	24.28148	29.84747	28.46119	24.28148	10.67877
21	0-back	31.76632	37.22465	19.30104	36.9971	19.84423	19.90808	35.32486	23.84859	28.18934	28.04491	28.18934	7.572981
22	0-back	34.48775	32.0826	19.69768	20.03624	21.41141	22.00039	20.47021	20.39114	21.34097	23.54649	21.34097	5.600723
23	0-back	30.38491	32.77734	24.45903	34.61244	17.93551	18.04583	27.96021	14.15896	31.86941	25.80041	27.96021	7.484627
24	0-back	35.70723	37.22545	19.586	19.40213	19.89752	20.28407	20.90812	20.48947	20.07796	23.73088	20.28407	7.244379
25	0-back	25.72388	52.1759	26.09157	38.52245	25.86869	27.30427	39.90759	34.14716	28.97474	33.19069	28.97474	8.993636
26	0-back	35.25804	35.83983	39.14667	26.09089	32.80734	18.93955	17.96166	18.32726	4.22011	25.39904	26.09089	11.4355
27	0-back	47.03201	46.71915	44.11129	42.40022	15.60126	47.03201	46.77736	44.21326	24.8583	39.86054	44.21326	11.48253
28	0-back	35.06581	33.4628	18.60364	18.44099	36.34687	19.46249	17.31329	18.21898	19.95619	24.09678	19.46249	8.211687
29	0-back	44.59429	44.12559	36.13453	36.40637	11.76413	44.45678	43.20617	36.37921	20.121	35.24312	36.40637	11.7155
30	0-back	33.84306	32.60276	34.60776	28.45284	41.58513	17.45716	32.40021	25.37142	24.89729	30.13529	32.40021	6.98001
31	0-back	37.90017	35.98498	18.46442	18.65114	18.1373	37.56678	35.86564	18.48583	20.76764	26.86932	20.76764	9.50057
32	0-back	46.89959	49.12013	27.24091	36.86375	12.18674	46.85745	49.11686	27.25003	19.61783	35.01703	36.86375	13.97165
33	0-back	31.54547	38.61099	19.98472	38.03894	11.30375	31.59514	37.12119	20.02191	21.32416	27.72736	31.54547	9.8274
34	0-back	22.99404	34.73536	19.77064	19.53255	19.69046	19.16836	23.31599	23.62254	19.96914	22.53323	19.96914	4.918305
11	stop	32.89235	32.35249	22.49334	24.44005	8.622168	16.96364	39.10201	26.23582	23.16398	25.14065	24.44005	9.078467
12	stop	30.39954	33.88017	19.65075	20.25016	21.13131	20.39262	36.36128	16.60721	30.28803	25.44012	21.13131	7.255345
13	stop	25.27295	24.72851	13.7764	12.31289	22.71253	24.89628	25.91607	36.75435	20.04834	22.93537	24.72851	7.223935
14	stop	13.7764	14.69359	23.58387	33.11901	20.24658	19.95466	20.25825	19.8183	31.08191	21.83695	20.24658	6.561682
15	stop	52.46385	49.46395	16.86187	47.59568	42.99889	19.54651	13.73482	16.43459	44.51666	33.7352	42.99889	16.49878

16	stop	10.84814	25.63252	32.33055	30.68225	26.07823	26.17724	25.63252	32.33057	18.16689	25.31988	26.07823	6.974564
17	stop	33.30764	30.67827	16.32043	16.14778	29.93343	18.11978	31.34284	18.97634	24.35486	24.35349	24.35486	7.069325
18	stop	33.09801	37.36255	36.22564	32.55048	41.0123	0.169405	42.46762	13.98844	19.42954	28.47822	33.09801	14.24301
19	stop	46.00001	43.53079	33.9191	32.52957	34.3111	20.84853	37.25703	19.1597	20.11425	31.96334	33.9191	9.977398
20	stop	37.49906	38.91243	20.20738	20.56658	23.4915	24.5885	41.08417	22.34883	21.14383	27.76025	23.4915	8.709762
21	stop	30.67149	33.81387	14.18052	36.50956	19.50336	19.68688	39.69031	23.02505	26.3767	27.05086	26.3767	8.671579
22	stop	33.56547	30.78504	18.87246	19.45313	3.831578	19.60646	20.19743	19.81647	3.566104	18.85491	19.60646	10.1355
23	stop	29.07799	29.91108	25.59696	28.64446	23.72567	19.03315	28.03634	24.69013	34.24268	26.99538	28.03634	4.337669
24	stop	15.607	15.57635	17.30674	16.91363	17.377	18.38391	29.1617	21.195	14.51459	18.44844	17.30674	4.457112
25	stop	33.80023	39.77747	31.76724	30.26156	22.69848	27.29616	47.11124	24.04751	30.1426	31.87806	30.26156	7.667776
26	stop	11.4076	28.81911	39.80497	29.29168	44.84436	19.81591	18.57917	18.41775	17.67536	25.40621	19.81591	11.14864
27	stop	35.41882	32.88619	36.43801	36.99882	31.63053	35.41882	32.88619	33.12239	14.45752	32.1397	33.12239	6.879232
28	stop	30.75531	27.68713	20.07554	20.05442	32.80752	21.88676	19.19932	21.14875	20.36013	23.77499	21.14875	5.198622
29	stop	44.14941	44.44755	38.80854	35.29971	10.49175	44.11166	44.66975	38.82446	17.9694	35.41914	38.82446	12.58883
30	stop	32.13129	28.87423	33.69559	33.65915	34.88391	19.00127	41.74629	25.65491	24.75893	30.48951	32.13129	6.706793
31	stop	43.16488	41.92743	18.18686	18.56443	7.762866	43.05534	38.76377	18.19349	22.46635	28.00949	22.46635	13.63876
32	stop	37.65571	40.18914	31.17894	32.35716	14.69378	37.74122	40.5943	31.14561	18.29909	31.53944	32.35716	9.305345
33	stop	34.49392	41.09179	21.71702	38.45776	17.48132	34.5285	44.50377	21.71853	20.52895	30.5024	34.49392	10.16711
34	stop	39.81395	40.49823	18.24345	18.2538	19.90227	20.17372	41.97019	23.89106	18.68228	26.82544	20.17372	10.60207
