# Supplementary material for: Signal Quality Evaluation of Emerging EEG Devices
Source: Front Physiol. 2018 Feb 14;9:98. doi: 10.3389/fphys.2018.00098 (PMC5817086; doi:10.3389/fphys.2018.00098)
Supplement: Supplementary file 1 [file DataSheet1.ZIP › SNR_BR8+.pdf]

BR8+ (all tasks)

SNR [dB]

Vp	Fp1	Fp2	Fz	C3	C4	Pz	O1	O2	mean	median	std
11	-11.3020782	-9.56242752	-6.02591658	0.78067601	-0.47385412	-1.34695458	-8.17659283	-5.76838779	-5.23444196	-5.89715219	4.45730299
12	-9.8944273	-11.0369797	-0.10639673	-10.3856258	-6.70644331	1.59623301	-7.51259136	-6.89282799	-6.3673824	-7.20270967	4.70126329
13	10.0468292	9.04009914	-1.01198637	-4.31041718	13.7089119	9.75492859	8.34288406	1.81305575	5.92303814	8.6914916	6.29728648
14	-19.5399361	-10.8130131	-9.87701225	-10.3153028	6.07990599	6.0239706	-4.19906139	-6.0191555	-6.08245057	-7.94808388	8.73283938
15	-12.7124701	-7.11079979	-10.5130243	7.71993828	-2.78116822	1.17401159	-6.97603655	-8.09060097	-4.91126876	-7.04341817	6.68220579
16	-5.16462564	-1.37621152	0.86716539	10.1110554	10.7143135	10.756299	10.4278173	9.43133831	5.72089397	9.77119684	6.523668
17	-0.14180753	-0.66265696	-2.94733334	-1.71982217	-4.44E+01	-0.63431454	-1.03808534	-2.42330885	-6.75004283	-1.37895375	15.2561196
18	-4.85798788	-8.95226955	-6.76457119	-6.16499853	-10.4107265	6.05264473	-8.55083752	-11.9369049	-6.44820642	-7.65770435	5.54847642
19	-10.0450172	-9.25219154	-9.8677454	-6.67406607	-11.0544167	4.47372913	-15.8586035	-16.0684509	-9.29334527	-9.95638132	6.42979277
20	-15.8554955	-2.50243807	-10.1888847	5.60815477	3.9595027	-9.97754955	-8.77288532	-10.4901648	-6.02747005	-9.37521744	7.60184914
21	11.5681801	11.6035852	-2.12095356	11.6369905	-3.7901597	-3.5765295	-1.64902377	-5.0504365	2.32770661	-1.88498867	7.74985061
22	-7.33201647	-5.98705578	-6.66293097	2.8521266	-27.3236122	-11.6339645	-9.63493347	-12.397665	-9.76500648	-8.48347497	8.51681296
23	-7.00567341	-9.72003174	-9.22327518	-11.7664528	4.32997561	10.1716824	5.19780016	7.30372763	-1.33903092	-1.3378489	8.90670724
24	-12.3486691	-2.55316043	-9.18942165	3.48199129	-6.96554756	5.62094736	2.24583983	-1.41318369	-2.64015049	-1.98317206	6.40278532
25	-26.5071011	-7.64252758	-6.26658392	-6.01108599	-1.68123007	-6.57274961	-8.3792696	-6.42759418	-8.68601775	-6.5001719	7.46818324
26	-13.7170324	-3.33505487	-5.84612799	3.36594057	1.71537244	-2.50950956	-3.61618614	-5.70571709	-3.70603938	-3.47562051	5.20687273
27	-13.3861628	-13.4955997	-15.2782173	-5.00713873	-8.8439188	1.0840292	-6.65759659	-10.4437304	-9.00354189	-9.64382458	5.39961522
28	-7.45494604	-7.44954634	-7.8530612	7.18597651	-1.45427418	-2.46036744	-9.37967205	-10.5809832	-4.93085924	-7.45224619	5.83067726
29	8.54581642	-0.42905453	3.15757489	3.23673892	-14.3362236	8.13924885	0.37379628	-0.16363311	1.06553302	1.76568559	7.13662703
30	-23.1676769	-8.57053089	-7.93848658	-9.51631451	-5.99509764	-4.10187197	-8.06021881	-6.48011303	-9.22878879	-7.99935269	5.88140248
31	-7.56227303	-5.27493334	1.10224211	-2.6275785	-35.8000145	-10.8791676	-8.71741486	-1.44485319	-8.90049911	-6.41860318	11.5724993
32	-1.33450389	-1.15080917	-3.00595999	9.0882864	12.2840986	9.18802738	11.4907999	10.1789932	5.84236656	9.13815689	6.46574869
33	-8.45191765	-17.0178642	-1.64020431	0.34076518	-25.9229221	-7.54787159	-16.9366989	-2.71073651	-9.98593127	-7.99989462	9.17501685
34	13.647397	12.7239065	1.66931486	16.7312088	14.4624166	14.5971251	9.8982048	16.7104912	12.5550081	14.0549068	4.91783783
