# Supplementary material for: Signal Quality Evaluation of Emerging EEG Devices
Source: Front Physiol. 2018 Feb 14;9:98. doi: 10.3389/fphys.2018.00098 (PMC5817086; doi:10.3389/fphys.2018.00098)
Supplement: Supplementary file 1 [file DataSheet1.ZIP › SNR_EPOC.pdf]

EPOC (all tasks)

SNR [dB]

Vp	AF3	F7	F3	FC5	T7	P7	O1	O2	P8	T8	FC6	F4	F8	AF4	mean	median	std
11	-16.1016788	-12.9653826	-12.9286718	-11.4568529	-9.33341503	-13.9515848	-15.0451593	-12.7419472	-10.7100515	-12.358675	-11.4573059	-12.1662512	-12.9817486	-13.6327343	-12.7022471	-12.8353095	1.7208299
12	-16.5030212	-15.9054918	-16.356741	-15.2285261	-17.1537285	-15.7019806	-16.3880844	-15.056778	-13.0548763	-13.5245142	-14.9834213	-15.8364687	-15.3335762	-15.8879328	-15.4939387	-15.7692246	1.11554902
13	-8.991436	-9.11595821	-9.15710258	-7.52406073	-8.44445229	-8.29765987	-8.86903477	-8.9949522	-8.16165638	-8.95127201	-8.13293171	-9.12242603	-8.8810873	-9.09167767	-8.6954077	-8.91617966	0.49856365
14	-15.6275606	-17.5226612	-29.8780079	-17.318964	-19.1540451	-20.1666737	-18.2174072	-16.7146893	-15.1635675	-14.9653301	-14.832262	-15.6505089	-15.5763931	-14.560626	-17.5249069	-16.1825991	3.9466169
15	-21.3243828	-20.5588093	-18.8073921	-19.4041882	-18.5918903	-19.1499596	-19.3220444	-12.617959	-13.3811607	-12.3565178	-16.8182983	-17.4905148	-18.0297775	-19.9708405	-17.7016954	-18.6996412	2.91002803
16	0.98570663	3.44664931	-12.2658653	3.79401016	4.22528601	3.14983582	3.69411016	3.26102662	0.84601218	4.62686634	1.6716094	1.26119876	3.13518333	3.97268271	1.84316515	3.20543122	4.24545666
17	10.0097752	9.72275829	9.37034988	-11.3078632	9.73983479	10.2542839	6.70847368	9.3965292	10.2824507	9.85917377	9.85807896	9.67409897	9.42320251	9.81690693	8.05771811	9.73129654	5.6405597
18	-12.6820221	-13.3996973	-12.4084959	-13.8128757	-20.1847878	-15.3897915	-14.0258598	-10.2914867	-10.95751	-11.9795399	-12.2517929	-11.7722521	-12.5719843	-11.8026886	-13.1093418	-12.4902401	2.41345394
19	3.79857302	4.9663105	2.68540692	-13.9565067	-1.81017041	-4.11412096	3.50519228	3.52496672	5.61237288	3.73198962	-9.80327892	-6.15507746	4.10295391	5.5311799	0.11569938	3.5150795	6.27975908
20	-13.7491159	-12.3068972	-8.46158981	-18.8951588	-19.9626923	-25.3439217	-28.0787487	-13.2378979	12.0160465	-10.3094864	-25.9474277	-20.5237122	-13.2977629	-12.9336777	-15.0737173	-13.5234394	9.92991116
21	-15.1705303	-15.1069326	-16.3033791	-13.8571053	-15.4955845	-16.5770187	-16.3558083	-15.1908941	-10.2115965	-12.7568541	-13.6934366	-14.3545351	-14.5628366	-14.8457355	-14.6058748	-14.9763341	1.65894401
22	-14.7048473	-17.7552872	-17.9824657	-18.2199898	-20.1810341	-23.3833885	-18.6193008	-17.1698437	-18.2568798	-15.3736591	-18.1754971	-17.3525581	-17.2314129	-17.3714008	-17.9841118	-17.8688765	2.04136183
23	-17.1997643	-13.4161196	-22.291153	-28.4266834	-22.3519173	-16.7435017	-28.4026756	-30.4451675	-27.559557	-15.3316832	-29.9118271	-29.9776115	-16.391655	-15.1408758	-22.3992994	-22.3215351	6.54020349
24	-15.3920126	-17.6355839	-19.8119812	-19.1107559	-15.1155844	-20.1896038	-18.2713184	-16.9846077	-21.5123806	-15.0421944	-15.4994259	-16.8092899	-18.1902676	-12.0069189	-17.2551375	-17.3100958	2.52086889
25	-17.9193535	-16.2181702	-18.6958084	-19.9981709	-19.1972599	-21.086319	-28.0460587	-29.1215076	-19.4802494	-15.2477398	-21.9727345	-19.1713085	-17.2721977	-17.0721359	-20.0356439	-19.1842842	4.05278369
26	-15.0116301	-13.2839289	-14.1648903	-16.4248962	-19.8518906	-17.2102089	-29.1250534	-13.3489771	-11.4893942	-11.7674055	-29.8859329	-13.2148981	-12.261095	-12.5018978	-16.3958642	-13.7569337	6.01509163
27	0.40850022	0.28888634	0.39301825	0.94289589	0.54418868	1.7111069	0.87575501	1.2626847	1.40202427	0.64988005	0.39496201	0.5910511	0.26501736	0.66268361	0.74233246	0.62046558	0.44437709
28	-12.5164423	-14.6559515	-15.4871807	-15.0418825	-17.5705872	-16.4639759	-17.6176434	-15.1802826	-13.7679911	-12.3499174	-14.7370453	-18.1275978	-14.6558514	-13.5538111	-15.1232971	-14.8894639	1.80916676
29	-16.8266811	-17.6072979	-23.2931118	-15.2353468	-17.1668835	-16.0401249	-12.4878206	-8.66513538	-7.75658321	-11.4867096	-14.1935492	-14.3425999	-15.9652319	-15.9041586	-14.7836596	-15.5697527	3.90435084
30	-12.8154554	-15.262681	-13.5068464	-14.660675	-15.8858204	-17.1424294	-17.7007084	-14.0456734	-13.1852369	-13.4753246	-13.1967258	-12.7131948	-13.5440865	-11.6946459	-14.2021074	-13.5254664	1.73197534
31	-18.2314796	-19.1984806	-19.2363605	-17.3743286	-17.0925751	-18.0621357	-19.68223	-18.5735149	-16.4654331	-15.274128	-16.1889591	-17.4379864	-16.9747734	-17.3443432	-17.6526234	-17.4061575	1.25785776
32	-15.8747053	-17.058939	-29.2508297	-18.5332031	-19.715435	-19.593071	-17.6772003	-15.654583	-15.1217051	-14.0245371	-14.7646561	-18.3882675	-15.5062218	-14.3331318	-17.5354633	-16.4668221	3.87004811
33	-12.9502831	-15.7144089	-18.389431	-14.6845245	-15.775835	-20.7670135	-30.0885334	-28.6398792	-28.3653927	-15.6184282	-28.4032078	-30.0124931	-16.7792492	-14.5165224	-20.7646573	-17.5843401	6.71432881
34	-16.429739	-17.5942841	-29.2999821	-29.7502689	-19.4316368	-25.363039	-19.573267	-16.2886105	-26.4937649	-16.662281	-28.8104992	-16.9093552	-16.657629	-15.5171261	-21.0558202	-18.5129604	5.54201246
