# Supplementary material for: Signal Quality Evaluation of Emerging EEG Devices
Source: Front Physiol. 2018 Feb 14;9:98. doi: 10.3389/fphys.2018.00098 (PMC5817086; doi:10.3389/fphys.2018.00098)
Supplement: Supplementary file 1 [file DataSheet1.ZIP › SNR_gLADYbird.pdf]

g.LADYbird (all tasks) SNR [dB]																			
Vp	Fp1	Fp2	F3	Fz	F4	T7	C3	Cz	C4	T8	P3	Pz	P4	PO7	PO8	Oz	mean	median	std
11	-6.98058367	-2.66200352	5.94594383	5.54431486	5.71597672	10.1090727	7.4326725	10.7486582	8.72081947	3.37599087	9.49768925	10.8049116	10.3127584	10.5125914	9.84177494	9.04825306	6.74805254	8.88453627	5.09230986
12	-11.0448828	-8.19163322	-1.69857669	0.3355352	-1.24662471	3.71208334	4.78342628	5.35373211	4.95845318	4.58612728	7.76459694	7.21162701	6.14263964	9.22157001	4.96081972	8.20072365	2.81560106	4.87093973	5.800413
13	-8.48187637	-9.2681818	-3.82035327	-3.58999634	-4.21338606	0.55353737	-2.47018743	-1.40598774	-2.27846456	1.71236563	-1.088588	-0.05099433	-0.04543006	0.26680359	-3.11605263	-6.70780516	-2.75028732	-2.37432599	3.20962993
14	1.22807097	2.9285326	4.46490908	6.82497835	6.88991833	5.97882128	7.57396936	7.18512344	7.78300142	10.2160234	6.79062462	7.09019518	7.29674053	8.44668102	8.05E+00	8.28E+00	6.689476	7.13765931	2.19338775
15	-17.5231457	-17.3087521	-6.30983496	-4.10408306	-5.31184053	2.84023404	2.11143637	5.08306456	2.49396992	7.47368622	8.4016161	10.5604744	9.53556061	12.4250698	12.9006634	11.6148109	2.18018313	3.9616493	9.81381689
16	-6.96858931	-7.8756156	2.7868793	3.80083799	1.59336925	-19.5673447	6.80799437	7.65399981	6.6238308	6.52769327	9.43646336	9.93862438	9.64909744	13.2265472	12.3690691	12.0704594	4.25458226	6.71591258	8.84900712
17	2.28975654	2.16333246	8.06205559	8.29166031	7.86682415	8.37170696	11.0132532	11.2937984	11.5734043	8.29794312	11.9940176	11.5136375	12.1428165	12.8526115	12.6978168	12.3495178	9.54838456	11.1535258	3.37975979
18	-7.90184402	-9.29549408	2.61906695	3.61953902	1.29223561	7.22854948	9.01501846	11.2925329	9.384758	7.02828693	13.1322241	13.678854	13.4663954	13.651022	14.8494883	13.8678789	7.30803199	9.19988823	7.54788582
19	1.76314545	0.19079609	8.78556728	10.629981	9.41637707	8.61166954	11.9384241	13.0512619	12.7011271	7.63183165	12.8497477	12.9951973	12.7700558	8.73272991	9.32703495	8.94667721	9.3963515	9.37170601	3.79720606
20	-0.70552647	-1.13491035	0.24727173	0.29235134	0.3446348	0.58594316	0.25472212	0.31995398	0.48033699	1.3766278	1.81033862	1.7157886	0.24196605	2.32042408	-24.1808739	-14.4591522	-1.90563148	0.30615266	7.08063368
21	-2.33794999	-3.64371943	4.69648647	6.39752579	5.67954254	7.44153929	7.85955	9.06482506	8.6321888	-34.3720856	11.9177408	12.3057442	11.3867111	7.22193289	10.8561945	10.7854404	4.61822918	7.65054464	11.3650684
22	-2.4467864	-6.5294652	1.68114889	1.99861717	0.82244438	5.19766235	5.80284786	6.0782814	6.37525177	5.70775366	1.48969829	9.0203495	8.33975506	8.67072964	8.84894371	9.05712509	4.38214732	5.75530076	4.53414605
23	-1.03281951	2.09055853	4.99268579	7.236413	7.32090044	8.17522907	8.10282135	9.41739273	9.33873177	9.9381628	10.1396885	-34.4765739	10.381175	12.5009327	12.8079166	11.8692245	5.55015247	8.75698042	11.2872358
24	-10.8745642	-4.92642212	-33.0458069	-2.78086281	-2.62072539	1.59262156	0.26950586	1.47662389	0.82191652	1.75282168	2.87677836	4.19835949	4.01461983	5.48974609	6.28411293	0.88971591	-1.53634745	1.1831699	9.42465693
25	-8.1434164	-19.0746078	0.12318279	0.71863383	-0.83751941	0.0683522	0.95665663	1.58837938	0.73006529	0.61819875	1.57497776	1.90211022	2.92383051	3.8268044	3.96292949	2.45148706	-0.41312096	0.84336096	5.68050826
26	-7.54268122	-7.86850214	-0.2810469	1.07976115	0.52106518	5.03635883	5.10451889	6.09039927	5.0513382	3.35335684	7.85144615	7.97498941	7.72382116	9.38600159	9.24674797	10.0810814	3.92554099	5.07792854	5.53274767
27	-7.22660208	-6.80271769	-0.73326659	0.74187303	-1.80104959	4.96708536	3.03580809	4.16166973	-6.38E-01	3.92111897	5.68777704	5.72241116	-0.68817186	8.28906822	7.0724411	8.45603848	2.13535351	3.47846353	4.84884472
28	-5.94501925	-3.31914997	-0.46831977	2.65561938	1.1353761	2.42563176	2.39800143	3.82356548	3.62546706	2.07799649	5.44870901	5.85338783	7.12855005	7.54684353	9.23519611	8.05148506	3.22958377	3.14054322	4.11169139
29	-11.6391735	-11.467845	-2.47049546	-0.633663	-1.94671953	2.3184948	2.673244	3.4512496	3.04763579	1.74370337	6.32655048	6.2492013	6.74015141	8.85976315	10.0102987	8.1084156	1.96067574	2.8604399	6.43679568
30	5.33015442	4.97068405	5.98477268	6.64483547	6.06561279	6.63350677	6.86791277	7.90421343	7.03346491	9.62469101	7.9050293	7.98662281	8.29415703	7.13281059	8.11514187	7.84281778	7.14602673	7.08313775	1.20812776
31	-6.16440916	-3.39267135	0.61603659	2.29306507	2.31315398	7.66912413	6.20290279	7.27732849	7.95721722	14.1376429	9.15008926	9.42934799	9.53495407	11.9139986	12.5226059	12.7035828	6.51024808	7.81317067	5.88500105
32	-4.10901546	-3.43999314	-1.64037383	4.26431942	3.92690802	7.21110249	5.81016731	5.93044138	6.01291037	5.98778725	7.37739658	7.42626667	7.47156572	9.71772194	9.36962032	9.78699398	5.06898869	6.00034881	4.39882413
33	-8.36482048	-8.05250835	-1.13635778	-0.46863809	-1.56306887	-0.57511592	1.17860603	2.03669047	-51.3708458	-2.0852406	-0.04228516	1.1934644	2.70643282	2.9326396	2.12263513	-7.4145689	-4.30643634	-0.52187701	13.0844784
34	-11.9159002	-11.5606031	-1.32929206	0.11048307	-1.69053829	5.12126827	3.51508212	4.12016296	2.84803391	1.3659898	5.93353224	5.74550152	5.79058552	6.23398066	5.07672691	5.05052233	1.52597097	3.81762254	5.77606583
