# Supplementary material for: Signal Quality Evaluation of Emerging EEG Devices
Source: Front Physiol. 2018 Feb 14;9:98. doi: 10.3389/fphys.2018.00098 (PMC5817086; doi:10.3389/fphys.2018.00098)
Supplement: Supplementary file 1 [file DataSheet1.ZIP › SNR_gSAHARA.pdf]

g.SAHARA (all tasks)

SNR [dB]

Vp	Fp1	Fp2	F3	Fz	F4	T7	C3	Cz	C4	T8	P3	Pz	P4	PO7	PO8	Oz	mean	median	std
11	-10.8216085	-3.22115088	-4.33539724	-12.744545	-17.1493607	-6.32600212	-13.409605	-26.0161915	-10.4395666	-5.73495865	-20.3188019	-9.44858742	-17.0762177	-12.6180067	-12.4434881	-14.8721476	-12.3109772	-12.5307474	6.02967355
12	-6.23094034	-10.2214231	-1.67877567	-3.87228942	-4.66689873	-4.3532629	-3.06073332	-5.82117987	-11.5894928	-5.27933741	-3.60914326	-7.10014868	-2.68804955	-2.99579191	-3.76198959	-9.35144234	-5.39255618	-4.51008081	2.8665157
13	-9.96616459	-9.39800644	-3.74024701	-7.82337236	-3.27668047	-10.4676723	-4.42653036	-5.32738447	-4.51923466	-8.21368217	-5.49343157	-1.37264287	-9.45815659	-8.4666748	-18.6550617	-17.8916302	-8.03103579	-8.01852727	4.81925339
14	4.01521969	2.36237931	5.41315269	7.98435545	8.21506596	10.9367561	10.1824236	8.92007542	10.5141792	12.7677546	13.0218296	13.1809931	13.2559309	16.3222008	14.86199	16.5117893	10.529131	10.7254677	4.19384352
15	-17.35993	-19.0108604	-7.59503937	-6.92729712	-10.2472754	-1.3316443	-0.45799008	-1.26797104	-1.31292188	-1.83308971	-1.38744128	-5.71974802	-9.61073399	-9.28293228	-13.4085579	-10.8189659	-7.34827492	-7.26116824	5.93407201
16	-0.90648729	-2.41301346	5.47593355	6.48618031	3.8136518	7.63868141	9.85467339	9.19016647	11.9748659	1.18714297	12.3019857	13.8662844	10.3501415	10.3886843	-4.04378462	7.93160343	6.44354436	7.78514242	5.48690435
17	-12.2884378	-1.58959067	7.88237381	6.86834192	6.12671995	0.94562554	4.50614786	7.71076965	3.95135546	-2.95711446	-0.55278891	-5.2884984	-0.81037199	-9.33870697	-6.80089426	-14.0357752	-0.97942778	-0.68158045	7.06841353
18	-1.09E+01	-1.28E+01	-2.63E+00	1.17E+00	-4.48E+00	-4.39E+00	3.49718189	3.06E+00	2.94E+00	-1.41143143	7.07687759	7.88E+00	7.77977896	9.25E+00	7.04182863	4.96E+00	1.13047416	2.99866021	6.69969147
19	-1.69155991	-1.59042537	10.3102598	7.11782646	1.88625872	10.3929472	10.626461	5.53173494	8.51604939	7.55559683	9.11724567	6.56565142	10.0673714	9.00568962	9.2027483	10.9007359	7.09466196	8.7608695	4.11431884
20	-5.56421614	-4.5928936	1.99221635	2.38406181	1.79822099	3.03872728	5.49749899	5.97102213	5.83933592	3.41539741	7.328619	8.06094646	7.06503677	6.42636108	5.67017126	6.00251484	3.77081379	5.58383512	3.96980698
21	-2.86276078	-5.21127605	4.51838684	3.73476934	1.84785008	-3.66816235	4.52442694	-22.8321152	-2.06328225	-6.98438597	-1.66141558	-1.45193088	-2.02024031	-0.79145783	-10.0440063	-15.0870419	-3.75329014	-2.04176128	7.27591403
22	-8.0440073	-9.93608856	-1.59830153	0.50441724	-0.71056527	1.99419701	2.35084462	-3.07879758	0.35004729	1.32379317	3.77330494	1.53948712	1.03346002	7.39619923	10.2453527	8.57189178	0.98220218	1.1786266	5.31031551
23	-1.88743091	-1.67029071	7.57208586	9.41315937	8.88598537	12.0555811	12.6451387	10.2606926	10.6656065	10.9822845	13.3810482	12.9089298	12.7280579	8.51977062	12.0794182	14.0559483	9.53724909	10.8239455	4.79277334
24	-12.1597099	-7.55633545	-3.87603951	-0.86219269	-0.94531852	6.12839174	4.54522514	2.63848019	4.93561268	9.45794487	3.78248692	9.44107819	5.71592045	8.65086651	8.02431107	1.13429689	2.44093866	4.16385603	6.22845064
25	-1.82435966	-5.92930937	-6.15255737	-0.18195325	0.63241416	-3.53069091	-2.88048005	-11.809824	-10.0868769	-5.86739206	-12.3232975	3.21895313	-9.80527973	-14.9025774	-13.797739	3.23127937	-5.75060566	-5.89835072	5.91800215
26	-6.47E+00	-7.99E+00	-0.85575294	-0.00695932	1.63E-01	4.58E+00	-0.54866964	-5.73161554	2.24E+00	0.56159008	0.90929455	2.87580204	0.05635054	-0.24996771	-1.8881501	1.36E+00	-0.68654186	0.02469561	3.39612347
27	-6.27233315	-6.24E+00	-0.07551601	-0.70986933	1.14336026	3.62080455	5.11427736	4.65574598	2.9733305	4.14111042	6.34775114	1.43251765	7.85441589	8.58474636	5.14613008	4.50881815	2.63884317	3.88095748	4.32209498
28	-0.80488837	-6.81229925	-8.63229084	-1.54859662	-2.685112	-3.27519584	1.13435757	-3.82959199	0.76116145	-0.08756351	-6.12591887	-4.39549303	-3.94569945	-13.3445101	-19.289917	-15.5248051	-5.52539768	-3.88764572	5.96421588
29	-16.894846	-13.3657598	0.28944337	0.7379421	-0.51290751	3.4395628	7.61659241	5.66912031	4.65125084	4.46445656	8.54692173	10.0509806	8.08504486	4.71634197	6.08682775	8.07174873	2.60329504	4.68379641	7.58933665
30	-4.9942894	-3.33932519	-7.44939661	-1.24170017	-4.8221755	-7.66706657	-10.3299198	-8.28412819	-3.9398582	-0.09737493	-5.43146658	-14.7977657	-5.02954721	-3.69292855	-5.74621344	-7.03423977	-5.86858724	-5.2305069	3.51089568
31	-12.3722095	-11.9782419	-2.81251359	-5.33224821	-1.72186279	4.01051903	-1.23033094	-14.2796021	0.42096552	0.34363517	-0.48890334	-1.91861737	5.03828859	4.00772953	5.75972891	2.61806703	-1.87097475	-0.85961714	6.25590093
32	-5.31331444	-6.12545013	3.66E-01	2.84E+00	3.34E+00	5.46E+00	5.05E+00	-5.61E+00	5.10E+00	4.65E+00	5.029109	2.71951056	3.95E+00	4.09372473	3.19E+00	-7.20E-01	1.75122321	3.26597631	4.05101092
33	-10.2015038	-15.2269716	-9.97010517	-8.25758553	-7.47231865	-5.74958754	-8.77241039	-12.2962408	-7.39828444	-8.8841114	-13.9705791	-15.1362553	-17.0961704	-22.6811409	-23.6505871	2.03773403	-11.5453824	-10.0858045	6.408305
34	-11.4667883	-14.4439564	-0.78411388	-1.26916051	-1.8743391	8.03050137	5.28429174	0.4609454	2.88147855	6.8210907	7.63688517	6.53667307	7.30368185	3.62426543	8.59906673	5.08526039	2.02661139	4.35476291	6.80650551
