# Supplementary material for: Signal Quality Evaluation of Emerging EEG Devices
Source: Front Physiol. 2018 Feb 14;9:98. doi: 10.3389/fphys.2018.00098 (PMC5817086; doi:10.3389/fphys.2018.00098)
Supplement: Supplementary file 1 [file DataSheet1.ZIP › SNR_Jellyfish.pdf]

Jellyfish (all tasks)
SNR [dB]

Vp	AF8	Fp2	Fp1	AF7	mean	median	std
11	-21.3693047	-24.9136009	-24.9284611	-23.1751404	-23.5966268	-24.0443707	1.69772574
12	-8.2053318	-8.87749958	-8.22952652	-8.71175289	-8.5060277	-8.47063971	0.34018874
13	-25.8307114	-26.3821316	-25.3563786	-23.2111168	-25.1950846	-25.593545	1.38747323
14	3.20372462	1.85142219	1.10532498	5.78835106	2.98720571	2.52757341	2.05951248
15	-21.7294865	-8.3134079	-21.648037	-19.5715523	-17.8156209	-20.6097946	6.41303629
16	-15.7193336	-14.1019945	-14.6384621	-28.9997482	-18.3648846	-15.1788979	7.12174419
17	-29.7313633	-32.7218819	-27.8522396	-30.847744	-30.2883072	-30.2895536	2.03959694
18	-10.6748714	-8.95714188	-11.7741919	-9.40203953	-10.2020612	-10.0384555	1.27606206
19	-1.11174512	-1.51312375	-14.8151264	-1.40396941	-4.71099117	-1.45854658	6.73822109
20	-5.11766148	15.857626	-4.0299902	-4.2205019	0.6223681	-4.12524605	10.1679046
21	-24.5206318	-9.16891193	-12.9255934	-24.2030678	-17.7045512	-18.5643306	7.83975442
22	-33.2319946	-10.0209808	-3.48462963	-30.8491421	-19.3966868	-20.4350615	14.8735997
23	-19.8077488	-7.04390764	-12.5379915	-19.3995991	-14.6973118	-15.9687953	6.09549919
24	-18.602993	-20.897543	-23.3006516	-31.0624008	-23.4658971	-22.0990973	5.41536253
25	-22.934433	-3.19864964	-6.43013668	-40.8264465	-18.3474165	-14.6822848	17.2998479
26	-10.123826	-8.8388319	-10.4693298	-10.0035524	-9.85888505	-10.0636892	0.70811337
27	-7.32375288	-5.23224735	-6.78442764	-6.68643999	-6.50671697	-6.73543382	0.89465898
28	-19.1730919	-28.2143726	6.90544271	-12.8042459	-13.3215669	-15.9886689	14.8933491
29	-10.0233555	-10.3910227	-6.80212927	-11.1770668	-9.59839356	-10.2071891	1.92528411
30	-7.34264183	-6.85062551	-18.3771954	-7.19360352	-9.94101655	-7.26812267	5.62789064
31	-36.4749031	-13.5553284	-11.8309212	-14.0514526	-18.9781513	-13.8033905	11.7032558
32	-24.3369942	-10.7541561	-16.2505531	-20.9370747	-18.0696945	-18.5938139	5.89709791
33	-34.9257927	-23.8750401	3.21181107	-17.150507	-18.1848822	-20.5127735	16.0366704
34	-18.8198051	11.6658716	-15.526103	5.36269474	-4.32933545	-5.08170414	15.1120638
